# Secondary Amine Catalysis in Enzyme Design: Broadening Protein Template Diversity through Genetic Code Expansion

**DOI:** 10.1002/anie.202403098

**Published:** 2024-04-19

**Authors:** Thomas L. Williams, Irshad M. Taily, Lewis Hatton, Andrey A Berezin, Yi‐Lin Wu, Vicent Moliner, Katarzyna Świderek, Yu‐Hsuan Tsai, Louis Y. P. Luk

**Affiliations:** ^1^ School of Chemistry and Cardiff Catalysis Institute Cardiff University Main Building, Park Place Cardiff CF10 3AT United Kingdom; ^2^ BioComp Group, Institute of Advanced Materials (INAM) Universitat Jaume I 12071 Castelló Spain; ^3^ Institute of Molecular Physiology Shenzhen Bay Laboratory Gaoke International Innovation Center Guangming District 518132 Shenzhen, Guangdong China

**Keywords:** Artificial Enzyme, Genetic Code Expansion, Organocatalysis, Secondary Amine Catalysis, Protein Engineering

## Abstract

Secondary amines, due to their reactivity, can transform protein templates into catalytically active entities, accelerating the development of artificial enzymes. However, existing methods, predominantly reliant on modified ligands or N‐terminal prolines, impose significant limitations on template selection. In this study, genetic code expansion was used to break this boundary, enabling secondary amines to be incorporated into alternative proteins and positions of choice. Pyrrolysine analogues carrying different secondary amines could be incorporated into superfolder green fluorescent protein (sfGFP), multidrug‐binding LmrR and nucleotide‐binding dihydrofolate reductase (DHFR). Notably, the analogue containing a D‐proline moiety demonstrated both proteolytic stability and catalytic activity, conferring LmrR and DHFR with the desired transfer hydrogenation activity. While the LmrR variants were confined to the biomimetic 1‐benzyl‐1,4‐dihydronicotinamide (BNAH) as the hydride source, the optimal DHFR variant favorably used the pro‐*R* hydride from NADPH for stereoselective reactions (*e.r*. up to 92 : 8), highlighting that a switch of protein template could broaden the nucleophile option for catalysis. Owing to the cofactor compatibility, the DHFR‐based secondary amine catalysis could be integrated into an enzymatic recycling scheme. This established method shows substantial potential in enzyme design, applicable from studies on enzyme evolution to the development of new biocatalysts.

## Graphical abstract

The importance of protein templates in artificial enzyme design is illustrated through genetic code expansion. Incorporation of a secondary amine into the nucleotide‐binding DHFR and multidrug‐binding LmrR resulted in catalytic entities, with the former favoring the use of NADPH as the hydride source for reactions, whereas the latter required biomimetic 1‐benzyl‐1,4‐dihydronicotinamide (BNAH).

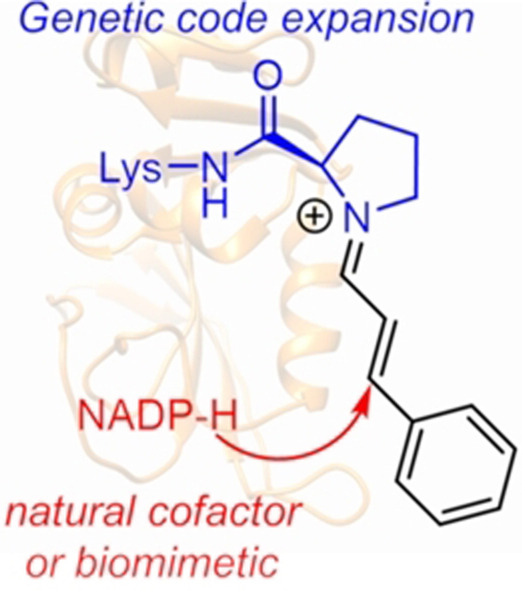

## Introduction

The use of secondary amines as catalytic motifs has tremendous potential in artificial enzyme design. In nature, primary amines including the amino groups of lysine residues and protein *N*‐termini are frequently involved in enzyme catalysis, serving as an acid, a base and/or nucleophile.[^1^, ^2^] In contrast, the use of secondary amines in enzyme catalysis is significantly rarer and their scope has not been fully explored.[^3^, ^4^, ^5^, ^6^, ^7^, ^8^, ^9^] While the basicity of cyclic secondary amines is similar to their primary counterparts, they are noticeably more nucleophilic for reactions with “latent” carbonyl substrates.[^10^, ^11^, ^12^] Furthermore, the iminium ion intermediate derived from secondary amine does not contain a proton on the nitrogen atom, thus prompting reactions with a latent nucleophile in the LUMO‐lowering pathway or a base for enamine formation in the HOMO‐raising pathway, driving the catalytic cycles forward.[^13^, ^14^, ^15^] Indeed, proline and its derivatives have been proposed to play roles in the prebiotic world, catalyzing the formation of crucial building blocks such as carbohydrates and nucleotides.[^16^, ^17^, ^18^] Given the unique reactivity of secondary amines, their incorporation into protein scaffolds can promptly generate catalytically active entities which have strong potentials to be transformed into highly active and selective artificial enzymes.

Currently, only a handful of protein templates have been used to generate protein‐based secondary amines for catalysis. In the first approach, 4‐oxalocrotonate tautomerase (4‐OT) contains an *N*‐terminal proline residue within its cavity, and it can be used to mediate iminium or enamine catalysis.[^19^, ^20^, ^21^, ^22^] In the second approach, pyrrolidines covalently linked to biotin were introduced as ligands to streptavidin (Sav), affording protein‐hosted secondary amine catalytic systems.[^23^, ^24^, ^25^] These systems could catalyze various types of chemical transformations, including conjugate addition,[^21^, ^22^, ^23^] aldol condensation,[^20^, ^24^] transfer hydrogenation^[25]^ and epoxidation.^[26]^ However, since there are only a few proteins that contain an *N*‐terminal proline within their cavity[^3^, ^6^] and also a few that bind biotin with significant affinity,[^27^, ^28^] the choice of protein templates for hosting secondary amine has significant limitation, posing challenges for both enzyme evolution studies and biocatalyst design.

Genetic code expansion has the advantage of fewer restrictions on the choice of protein scaffolds and the site therein.[^29^, ^30^, ^31^, ^32^, ^33^] Previously, an aniline motif has been added to the multidrug binding protein LmrR as an unnatural amino acid (UAA).[^29^, ^30^, ^31^, ^32^] Whilst being less nucleophilic than pyrrolidine,[^10^, ^12^] the aniline in LmrR was able to catalyze various carbon‐ heteroatom and carbon‐carbon ligation reactions.[^29^, ^30^, ^31^, ^32^] Other examples include the use of *N*_δ_‐methylhistidine and 4‐benzoylphenylalanine to mediate acyl cation and triplet energy transfer catalysis, respectively.[^33^, ^34^] Secondary amines have been incorporated into protein scaffolds (e.g., β‐galactosidase) as pyrrolysine analogues, in which proline and thiazolidine derivatives were attached to lysine through isopeptide bonds.^[35]^ Recently, Gran‐Scheuch et al. integrated some of these amino acids as well as a new piperidine analogue into a model protein template LmrR; however, their activity screening based on conjugate addition yielded low conversion with negligible stereoselectivity^[36]^ (the preprint of the current manuscript^[37]^ and a concurrent article by Yu et al also reported testing the activity of some of these UAAs).

**Figure 1:**
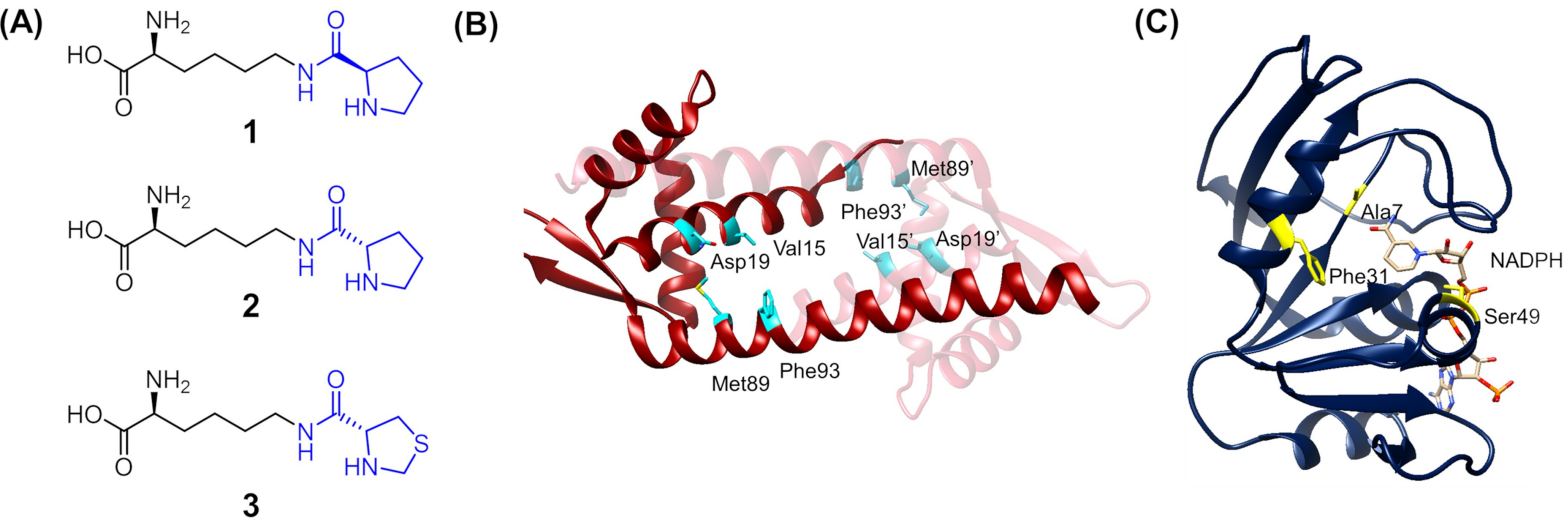
Components used in the design of the protein‐hosted secondary amines. A) The structures of the unnatural amino acids (UAAs) used include D‐prolyl‐L‐lysine (**1**), L‐prolyl‐L‐lysine (**2**) and L‐thiazolidine‐L‐lysine (**3**, thioproline). B) The X‐ray crystal structure of the *Lactococcus* multidrug resistant regulator LmrR (PDB: 3F8F). Two monomers are shown in red and light red. Residues targeted for UAA incorporation are highlighted in cyan. C) The X‐ray crystal structure of *E. coli* dihydrofolate reductase (DHFR) with NADPH bound in the active site (PDB: 1RA1). Residues targeted for UAA incorporation are highlighted in yellow.

Here, through genetic code expansion, UAAs **1**–**3** bearing a cyclic secondary amine (Figure 1A) were site‐specifically introduced into different scaffolds, including the super folder green fluorescent protein (sfGFP), multidrug‐binding protein LmrR and nucleotide‐binding dihydrofolate reductase (DHFR).^[38]^ Hydrolysis of the isopeptide bond was observed for proteins incorporated with **2** (L‐proline) but not detected for proteins incorporated with **1** (D‐proline) or **3** (L‐thioproline). Incorporation of **1** resulted in highly active entities. Importantly, the effect of expanding the protein template option was demonstrated by examining the reactions hosted by the respective LmrR and DHFR variants (Figure 1B,C). Both systems could catalyze the model reduction of various α,β‐unsaturated aldehydes. However, while the LmrR system could only use the biomimetic 1‐benzyl‐1,4‐dihydronicotinamide (BNAH) as a source of hydride, the DHFR system could expand its nucleophile option to NADPH recruiting the pro‐*R* hydride for stereoselective transfer hydrogenation. Modifying the active site residues or implementing a basic condition in the DHFR system, as shown by experimental and computational studies, leads to reactions with an enantiomeric ratio (*e.r*.) of up to 92 : 8. To conclude its dependence on NADPH for catalysis, the DHFR‐hosted reaction was coupled to an enzymatic cofactor regeneration scheme.

## Results and Discussion

UAAs **1**, **2** and **3**, derived from D‐proline, L‐proline and L‐thiazolidine‐4‐carboxylic acid (L‐thioproline), respectively, each contain a secondary amine and were tested for incorporation (Figure 1A). Based on previous reports, **1** and **3** are substrates of the wild‐type *Methanosarcina bakeri* pyrrolysyl‐tRNA synthetase (MbPylRS) and its engineered variant ThzKRS, respectively.[^35^, ^39^] Due to the structural similarity between **2** and **3**, we envisioned that **2** could be a substrate of ThzKRS. Incorporation of UAAs was tested using a version of sfGFP whose gene bears a TAG codon for the 150^th^ amino acid residue (Asn150TAG), and formation of the full‐length protein implied successful incorporation of the UAA as indicated by SDS‐PAGE analysis (Figure S1 and Table S1).^[40]^ However, since the corresponding UAA residue is located at a solvent‐exposed position, rather than the interior of the protein, their stability and catalytic activity were not further examined.

**Figure 2:**
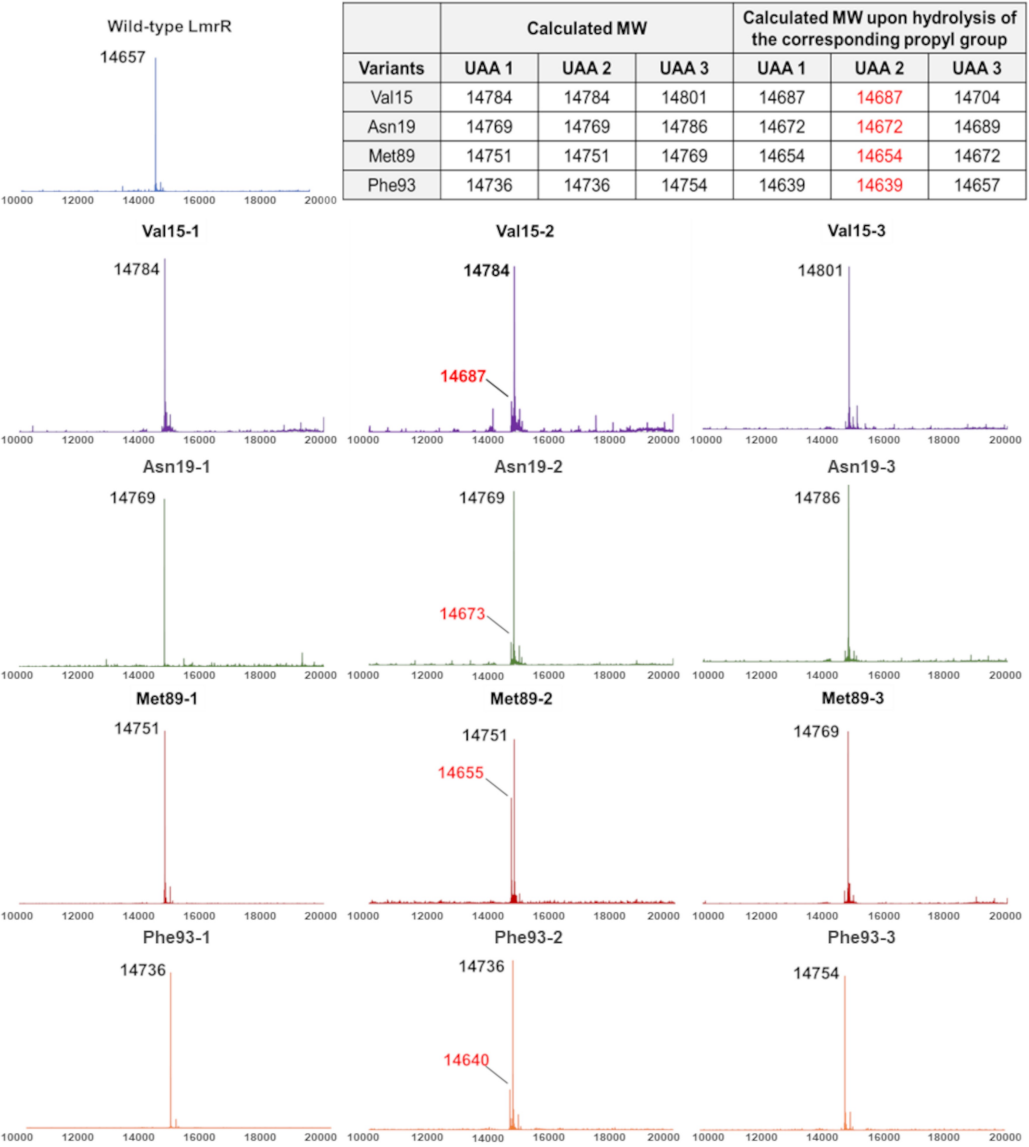
Deconvoluted ESI mass spectra for the wild‐type LmrR (calculated to be 14657 g/mol) and its variants, in which Val15, Asp19, Met89 and Phe93 were individually replaced with UAA **1**, **2** or **3**. All 13 proteins were found to have the N‐terminal methionine removed. For variants incorporated with **2**, profound peaks that match closely to the hydrolysis of the L‐prolyl group were observed (red fonts).

For catalyst design, the UAAs were incorporated into the multidrug binding protein LmrR,^[41]^ a scaffold known to be catalytically competent after modification of its hydrophobic pocket including the insertion of UAAs.[^42^, ^43^, ^44^, ^45^] Hence, we selected four previously reported residues, Val15, Asn19, Met89 and Phe93 (Figure 1B), located in the hydrophobic pocket for substitution with **1**, **2** or **3**. The 12 LmrR variants were successfully produced by *Escherichia coli* as revealed by SDS‐PAGE analysis (Figure S2). The dimeric nature for most of these variants was confirmed by size exclusion chromatography (see SI, Pg S8–10). However, liquid chromatography‐mass spectrometry (LC‐MS) investigation indicated that variants containing residue of UAA **2** were relatively unstable and up to about 35 % hydrolysis of the L‐prolyl group was observed at position 89 (Figure 2). Direct incorporation of lysine was highly unlikely because the full‐length protein could not be obtained without supplementing **2** in the growth cultures (Figure S1). Hence, this observation was attributed to the hydrolysis of the L‐prolyl group during protein production, as *E. coli* contains enzymes such as proline iminopeptidase which can cleave the *N*‐terminal proline in polypeptides (e.g., UniProt: A0A6N7NN15_ECOLX). In contrast, truncation was not observed in proteins incorporated with either D‐proline **1** or L‐thioproline **3**, and hence they were concluded as non‐hydrolyzable during the protein preparation process.

**Figure 3:**
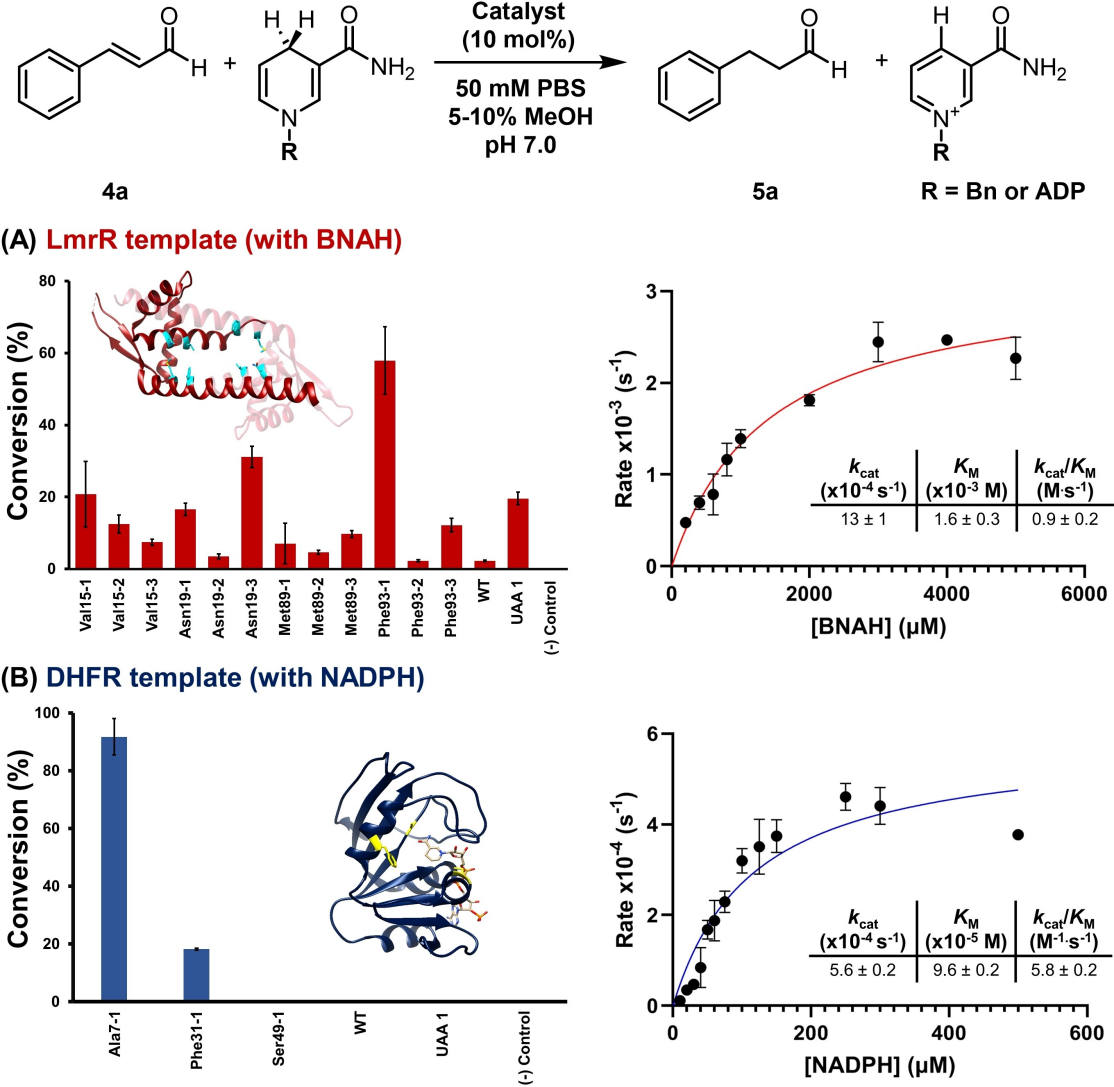
Assessment of the catalytic efficiency in the transfer hydrogenation reaction. A) Conversion of the model reaction catalyzed by the LmrR variants using BNAH as determined by GC‐MS (see SI). The model organocatalytic transfer hydrogenation reaction contained cinnamaldehyde (0.68 mM, 1 equiv.) and LmrR variants (68 μM, 10 mol %), and then BNAH (1.36 mM, 2 equiv.) was added and stirred for 18 h in PBS buffer (pH 7.0, 10 % methanol) at 25 °C. The estimated turnover (*k*_cat_) and Michaelis (*K*_M_) constants at 25 °C for **LmrR‐Phe93‐1** (50 μM) using 1 mM of cinnamaldehyde (**4 a**) and various concentrations of BNAH (200–5000 μM) were reported. B) Conversion of the model reaction catalyzed by the DHFR variants using NADPH as determined by GC‐MS. The model organocatalytic transfer hydrogenation reaction contained cinnamaldehyde (**4 a**, 0.52 mM, 1 equiv.) and DHFR variants (51 μM, 10 mol %), and then NADPH (1.04 mM, 2 equiv.) was added and stirred for 18 h in PBS buffer (pH 7.0, 5 % methanol) at 25 °C. The estimated turnover (*k*_cat_) and Michaelis (*K*_M_) constants at 25 °C for **DHFR‐Ala7‐1** (50 μM) using 1 mM of cinnamaldehyde (**4 a**) and various concentrations of NADPH (40–500 μM) were reported. The template experiments (A) and (B) were tested alongside with the wild‐type (WT) proteins, UAA **1** and a negative (−) control where the reaction was performed without any protein or catalyst. Each reaction was performed in triplicate and the mean value (±standard deviation) is shown.

We chose the conversion of cinnamaldehyde **4 a** to its reduced counterpart **5 a** as the model reaction (Figure 3) because the streptavidin (Sav)‐hosted pyrrolidine was shown to be able to catalyze this reaction.^[25]^ BNAH, unlike other biomimetics such as Hantzsch ester, is soluble in aqueous buffers and thus was tested as the hydride donor.^[25]^ Conversion of the reaction was estimated using a gas chromatography‐mass spectrometry (GC‐MS) setup as previously described^[46]^ (see also Figure S13). Though containing seven lysine residues and a free *N*‐terminal amino group, the wild‐type LmrR could only afford less than 3 % of conversion implying minimal catalytic activity (Figure 3A and Table S2). Substitution of Met89 with **1**, **2** or **3** showed marginal improvement (ca. 5 % conversion). For all the variants incorporated with **2** of which different degrees of truncation was detected, low conversion (<15 %) was observed. Substitution of thioproline **3** at position 19 and D‐proline **1** at position 15 resulted in >20 % product conversion. Nevertheless, the replacement of Phe93 with **1** (**LmrR‐Phe93‐1**) was most promising giving a yield 19‐fold higher than that of the wild‐type enzyme (58 % vs. 3 %). Differed by only one stereocenter (D‐ vs L‐proline), **LmrR‐Phe93‐2** retained a majority of the secondary amine (ca. 80 %, see Figure 2) but its conversion remained low (<2 %). On the other hand, **LmrR‐Phe93‐3**, the thiazolidine equivalent of **LmrR‐Phe93‐2**, was able to provide a higher conversion (12 %). Accordingly, factors including the chirality, amino acid stability, electronic property of the organocatalytic motif as well as the surrounding microenvironment may contribute to the catalytic efficiency.

NADPH is structurally more complex than BNAH but also a hydride donor frequently used in biological contexts. Testing if the protein‐based secondary amine system could use a nucleophile other than small molecule BNAH, the **LmrR‐Phe93‐1** reaction mentioned above was repeated using NADPH as a source of hydride, but less than 4 % of conversion was detected. This observation are consistent with the literature. The secondary amines hosted by Sav could not recruit NADH for reaction under aqueous conditions.^[25]^ Similarly, NADH proved to be a suboptimal hydride donor for small organocatalytic secondary amines.[^47^, ^48^] Indeed, while examples of metal catalysts and artificial metalloenzymes using NAD(P)H or NAD(P)^+^ for reactions have been reported,[^49^, ^50^, ^51^, ^52^, ^53^] the equivalents in organocatalytic systems are much less common.

Seeing if NADPH can be used as a hydride source through a switch of template, the *E. coli* dihydrofolate reductase (DHFR) was tested. DHFR catalyzes the step of hydride transfer from the C4 pro‐*R* hydride of NADPH to C6 of dihydrofolate.[^54^, ^55^, ^56^] Containing a Rossmann fold, DHFR binds nucleotide‐containing cofactor NADPH with an association rate up to 10^5^ M^−1^ ⋅ s^−1^ (Figure 1C).[^55^, ^56^] Additionally, it contains a mobile M20 loop (residue 9–23) which closes the active site upon binding NADPH, thereby generating a shielded environment surrounding the nicotinamide motif.[^57^, ^58^] These features present DHFR as a plausible scaffold for organocatalytic transfer hydrogenation by NADPH. Hence, Ala7, Phe31 and Ser49 that are in proximity to the nicotinamide motif were individually replaced with **1**, the non‐hydrolyzable UAA that gave the highest conversion in the LmrR system. The resulting DHFR secondary amines were verified by SDS‐PAGE (Figure S3) and mass spectrometry (Figure S4). Similarly, hydrolysis of the D‐prolyl group was not detected.

Activity tests revealed that the DHFR variants incorporated with **1** could facilitate NADPH‐dependent transfer hydrogenation (Figure 3B). For the wild‐type DHFR which contains six lysine residues and an *N*‐terminal amino group, the reaction product **5 a** could not be detected by GC‐MS analysis. Replacement of Ser49 with **1** led to considerable protein precipitation with no detectable products. Contrarily, replacement of Ala7 with **1** could effectively catalyze the reaction as evident by significant product conversion (Figure 3B and Table S3). With 10 mol % of **DHFR‐Ala7‐1**, 92 % of starting material **4 a** was converted into product **5 a** in aqueous PBS buffer at pH 7.0 when 2.0 equivalents of NADPH were introduced for 18 h, while 34 % conversion was achieved with BNAH under the same condition (Table S4). Under the same conditions, NADPH could not be used as a hydride donor for secondary amines including pyrrolidine, UAA **1** and the first‐generation MacMillian catalyst (Figure S5).

**Figure 4:**
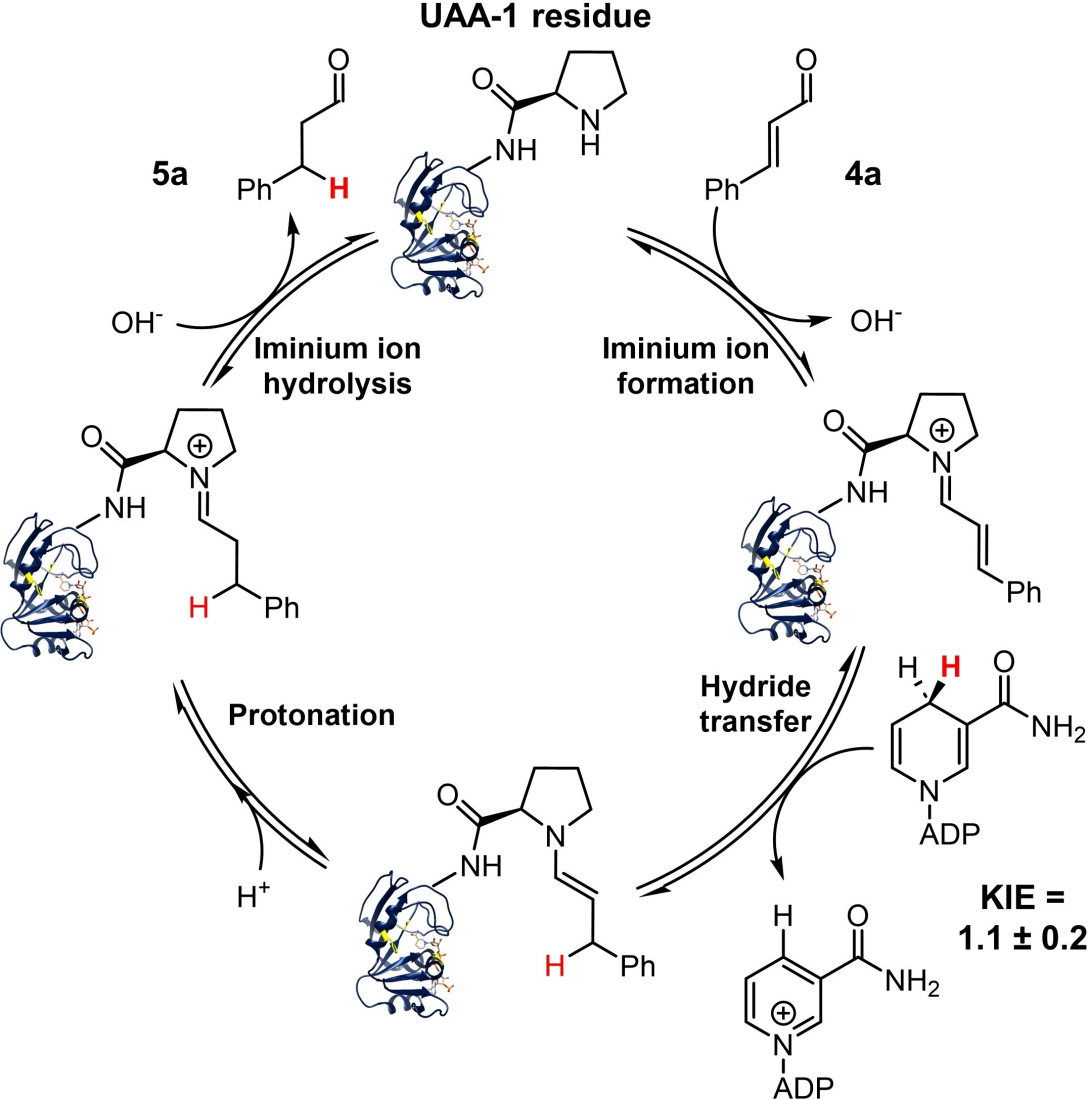
Proposed catalytic cycle of the organocatalytic transfer hydrogenation reaction by **DHFR‐Ala7‐1** based on the iminium trapping and kinetic isotope effect (KIE) experiments (see also Figures S6–S8 and S11). The residue of UAA **1** forms an iminium ion with the α,β‐unsaturated carbonyl substrate, and hydride transfer occurs from the pro‐*R* position of NADPH to C_β_ of the iminium intermediate. A KIE (*k* ^NADPH^/*k* ^NADPD^) of 1.1±0.2 was measured using NADPH and [4*R*‐^2^H]‐NADPH (NADPD) during catalysis.

Similar to many other secondary amine organocatalysts,^[11]^ formation of iminium ion is critical towards carbonyl substrate activation, and hence its transient formation during the catalysis by **LmrR‐Phe93‐1** and **DHFR‐Ala7‐1** was probed. The two variants were incubated with cinnamaldehyde **4 a**, treated with NaCNBH_3_ and subjected to high‐resolution mass spectrometry analysis, similar to previously described (see SI).^[30]^ The **LmrR‐Phe93‐1** sample predominantly yielded one protein species with an observed mass matching the calculated molecular weight of the reduced covalent protein‐substrate intermediate (Figure S6A). Similarly, approximately 30 % of the **DHFR‐Ala7‐1** variant also yielded the reduced covalent intermediate under the same condition. However, there was a noticeable amount of unmodified **DHFR‐Ala7‐1**, consistent with the crystal structure analysis which indicated that the modified site is relatively inaccessible (Figure S6B).[^57^, ^58^] Treatment of these protein species with chymotrypsin revealed digested peptides, whose elemental composition identified by liquid chromatography mass spectrometry (LC‐MS) corresponds to the trapped intermediate (Figures S7 and S8). In contrast, the wild‐type LmrR and DHFR that cannot catalyze the transfer hydrogenation did not yield any evidence of the iminium intermediate formation. Accordingly, these results support a LUMO‐lowering reaction mechanism,^[11]^ in which the secondary amine activates the starting material through iminium ion formation for transfer hydrogenation (Figure 4). Contrary to the previous work which used a LmrR‐hosted aniline for catalysis and 4‐nitrobenzaldehyde as substrate,^[30]^ no lysine modification was detected in our protein scaffolds after the cinnamaldehyde and NaCNBH_3_ treatment. This difference in chemoselectivity suggested that the secondary amine is more reactive than lysine, being able to activate a relatively inert carbonyl substrate such as **4 a**. Furthermore, the catalysis by aniline was found to be most active at position 15 in LmrR,^[30]^ whereas we obtained higher conversions with the secondary amine organocatalyst at position 93, implying that activity test at different positions is an essential step during catalyst design.

The estimated *k*_cat_ constants offer insights into the relative accessibility of the secondary amines in various systems. The highest *k*_cat_ constant was observed in the previously reported streptavidin‐hosted secondary amine system (>20‐fold, ca. 0.01275 s^−1^),^[25]^ followed by LmrR (ca. 2‐fold, 0.00013 s^−1^), and eventually DHFR (0.00056 s^−1^) which recruits NADPH instead of BNAH as hydride donor (Figures 3 and S9; Tables S5 and S6). A plausible explanation is that the Sav‐hosted secondary amine is solvent‐exposed,^[23]^ and thus it can readily react with the carbonyl substrate resulting in a larger *k*_cat_ constant. In contrast, in both **LmrR‐Phe93‐1** and **DHFR‐Ala7‐1**, the secondary amines are relatively inaccessible, located within the protein cavities, and so lower *k*_cat_ constants were observed. Indeed, the lower *k*_cat_ constant for **DHFR‐Ala7‐1** also aligns with the iminium trapping experiment discussed above. However, the estimated Michaelis constant (*K*_M_) value for the corresponding hydride donor was found to be lowest for DHFR (96 μM), resulting in an enhancement in the bimolecular rate constant that is approximately 6‐fold higher than that of the LmrR system (Figure 3; Table S5 and S6). The *K*_M_ constants for cinnamaldehyde **4 a** were found to be too high for determination, indicating a weak affinity between the substrate and protein. Furthermore, the estimated bimolecular rate constants for all three systems were considerably lower than those for natural enzymes (e.g. old yellow enzymes) that recruit BNAH or NADPH for reactions.[^59^, ^60^] Nevertheless, this work has demonstrated that secondary amines, such as **UAA 1**, are useful motifs for converting protein templates into active entities, whereas selecting an appropriate template can broaden the nucleophile options for reactions. Furthermore, performance of these systems can be further enhanced through laboratory evolution.[^29^, ^61^, ^62^]

To examine the stereochemistry of the **DHFR‐Ala7‐1** reaction, we recruited the [4*R*‐^2^H]‐NADPH (NADPD) as a reagent. Only one isotopologue was obtained as product, with the deuteride located at the C_β_ position as illustrated by the GC‐MS analysis (Figure S10). This observation agrees with previous crystal and biochemical analysis of wild‐type DHFR which illustrated stereospecific transfer of the C4 pro‐*R* hydride from NADPH.[^63^, ^64^] Furthermore, this protein catalyst is regioselective favoring 1,4‐ over 1,2‐addition, the latter was observed in the Sav‐hosted secondary amine catalytic system as a side reaction.^[24]^ The turnover rate constants under saturating conditions between the NADPH and NADPD reactions yielded an estimated kinetic isotope effect (KIE) of 1.1±0.2 (Figure S11), implying that the step of hydride transfer is not rate‐limiting. Other chemical step(s) such as iminium ion formation can be rate‐limiting;^[65]^ alternatively, the physical step of releasing the oxidized cofactor can dictate the rate of catalytic turnover, as observed in the natural reaction catalyzed by the wild‐type DHFR.[^55^, ^58^]

**Figure 5:**
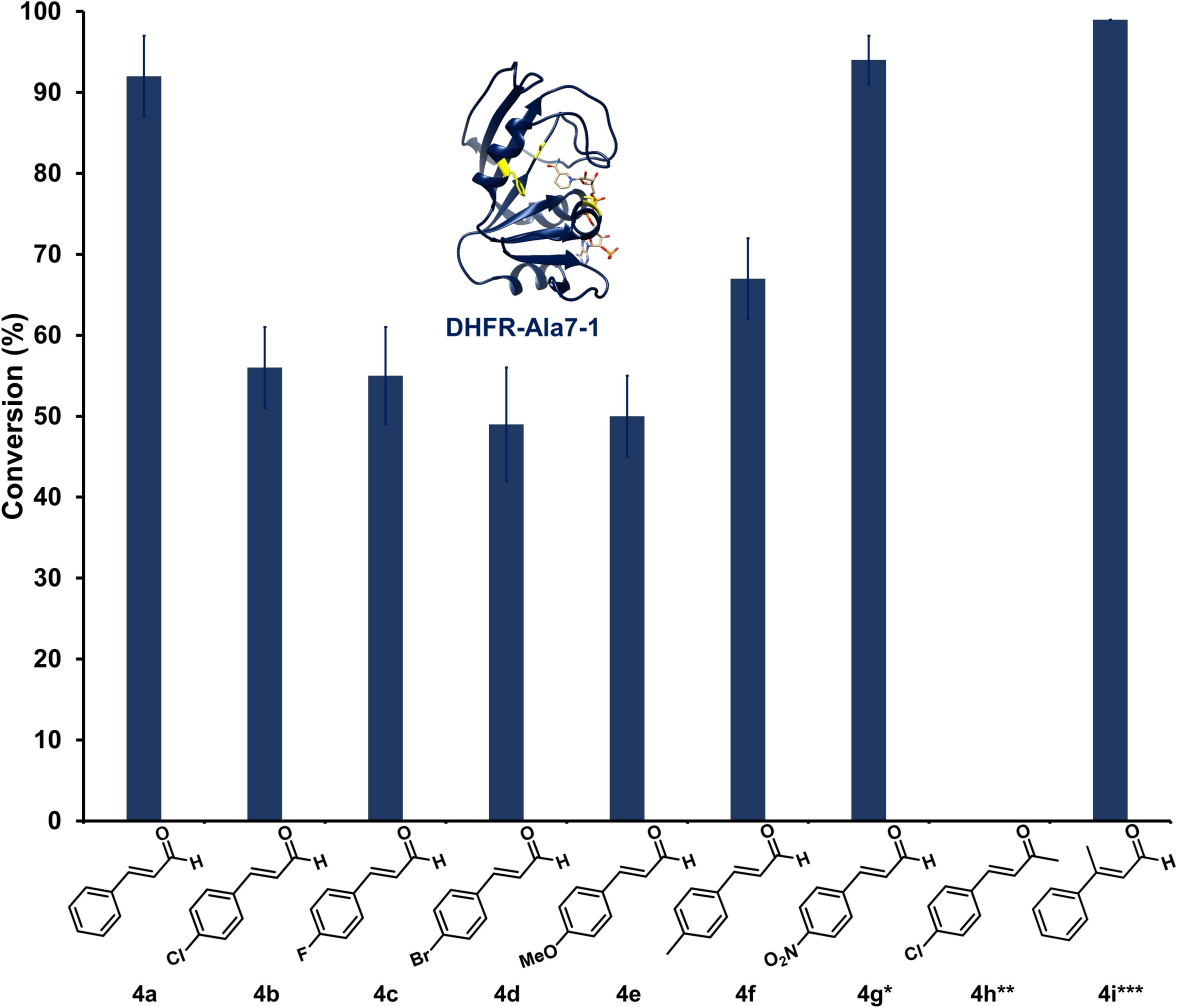
Substrate scope analysis of **DHFR‐Ala7‐1**. The DHFR variant (5.1 μM, 10 mol %) was introduced with the indicated α,β‐unsaturated carbonyl compound (**4 a**–**4 i**, 0.51 mM, 1 equiv.) and NADPH (3.4 mM, 5 equiv.) in PBS buffer at pH 7.0 for 48 h. Each reaction was performed in triplicate and the mean yield (±standard deviation) reported. Conversion (%) was estimated by use of ^1^H NMR spectroscopy as previously described (see Table S7 and Figure S18 in SI).[^23^, ^24^, ^25^] *Over 50 % of the conversion resulted in unidentified byproduct as indicated by ^1^H NMR spectroscopy. **The product was detectable by GC‐MS but not NMR spectroscopy. ***20 % of the substrate was converted into an unidentified product as revealed by ^1^H NMR spectroscopy. See Section 17 in Supporting Information for corresponding data.

To further analyze the reaction stereochemistry, the substrate scope of the DHFR‐hosted organocatalytic system was first examined. Cinnamaldehyde analogues with different substituents at the *para*‐position (Cl, F, Br, OMe, Me; **4 b**–**f**) were found to be viable substrates for transfer hydrogenation, as indicated by ^1^H NMR spectroscopy and GC‐MS analyses (Figure 5, Table S7, Figure S18). This broad substrate scope suggests that the active site of DHFR is capable of accommodating a range of aldehydes and has yet to be optimized for enhanced affinity. However, the introduction of an electron‐withdrawing nitro (NO_2_) group (**4 g**) led to side reactions, as evidenced by the formation of various unidentified byproducts (ca. 50 % of substrate conversion) in the ^1^H NMR spectroscopic analysis. Conversely, the ketone equivalent (**4 h**) exhibited minimal turnover, with the corresponding product detectable only by GC‐MS but not ^1^H NMR spectroscopy. The prochiral substrate (*E*)‐3‐phenylbut‐2‐enal (**4 i**)^[66]^ could also serve as a substrate for **DHFR‐Ala7‐1**, predominantly yielding the dihydro‐product (**5 i**, 80 %) and an unidentified byproduct that likely arises from deprotonation of the methyl group (20 %). Nevertheless, this prochiral substrate allowed us to analyze the stereochemistry of the **DHFR‐Ala7‐1** reaction (see below).

When prochiral substrate **4 i** was used in the **DHFR‐Ala7‐1** reaction at pH 7.0, the enantiomeric ratio (*e.r*.) was measured to be 71 : 29 (*S*‐ to *R*‐enantiomers), indicating moderate stereoselectivity. While both the substrate conversion and *e.r*. decreased under acidic conditions, enantioselectivity was found to be enhanced under basic conditions, favoring formation of the *S* stereoisomer with an *e.r*. of 88 : 12 (Table 1, Figures S12 and S18). Initially, the titratable residue(s) in spatial proximity to the UAA, namely Asp27 and Tyr100, were proposed to be deprotonated under alkaline conditions, leading to a change in the electrostatic environment and hence the stereochemical outcome. Replacement of Asp27 with either asparagine (D27N) or alanine (D27A) did not significantly affect the stereochemical outcome. Similarly, the replacement of Tyr100 with glutamate (Y100E) did not affect the stereochemistry. Contrarily to the initial hypothesis, the **DHFR‐Ala7‐1** variant possessing a Y100F mutation resulted in an enhancement of enantioselectivity at neutral pH, resulting an *e.r*. of 84 : 16, which was further enriched to 92 : 8 at pH 11.0.

**Table 1:** Reduction of (*E*)‐3‐phenylbut‐2‐enal (**4 i**) by **DHFR‐Ala7‐1** and its variants.^[a]^

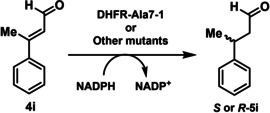			
Variant	pH	Substrate conversion^[b]^ / %	e.r./S : R
**DHFR‐Ala7‐1**	4.0	5	50 : 50
**DHFR‐Ala7‐1**	5.0	9	58 : 42
**DHFR‐Ala7‐1**	6.0	12	62 : 38
**DHFR‐Ala7‐1**	7.0	>99	71 : 29
**DHFR‐Ala7‐1**	8.0	95	72 : 28
**DHFR‐Ala7‐1**	9.0	90	80 : 20
**DHFR‐Ala7‐1**	10.0	87	83 : 17
**DHFR‐Ala7‐1**	11.0	>99	88 : 12
**DHFR‐Ala7‐1 D27N**	7.0	99	73 : 27
**DHFR‐Ala7‐1 D27A**	7.0	96	65 : 35
**DHFR‐Ala7‐1 Y100F**	7.0	95	84 : 16
**DHFR‐Ala7‐1 Y100E**	7.0	93	64 : 36
**DHFR‐Ala7‐1 D27N**	11.0	>98	91 : 9
**DHFR‐Ala7‐1 D27A**	11.0	>98	88 : 12
**DHFR‐Ala7‐1 Y100F**	11.0	>98	92 : 8
**DHFR‐Ala7‐1 Y100E**	11.0	>98	73 : 27

[a] The reaction contained (*E*)‐3‐phenyl‐2‐butenal (**4 i**, 2.7 mM, 1 equiv.) and DHFR‐Ala7‐1 (10 mol %, pre‐equilibrated in buffers at the corresponding pH), and then NADPH (5.4 mM, 3 equiv.) was added and stirred for 18 h at 25 °C. For reactions at pH 4.0–5.0, formate (50 mM formic acid, 150 mM NaCl) was used; for reactions at pH 6.0–8.0, phosphate buffer (50 mM NaPi, 150 mM NaCl) was used; for reactions at pH 9.0–11.0, carbonate‐bicarbonate buffer (50 mM NaHCO_3_; 150 mM NaCl) was used. [b] GC‐MS was used to estimate the conversion of **4 i** but the possibility of byproduct formation, occurring at 20 % at pH 7.0, cannot be ruled out.

Investigating the experimental observations, DHFR with Ala7 replaced with the UAA **1** was computationally prepared based on a crystal structure available in the Protein Data Bank (PDB ID : 1RX2)^[63]^ and equilibrated by running 500 ns of molecular dynamic (MD) simulations in the Isothermal‐Isobaric ensemble, NPT, at 277 K and 1 bar (see Supporting Information for details). Subsequently, (*E*)‐3‐phenylbut‐2‐enal (**4 i**) was covalently attached to the secondary amine and converted into the iminium ion intermediate. This resulted in two DHFR models: one with the C‐3 pro‐*S* face and the other with the pro‐*R* face directed towards the nicotinamide motif of NADPH (see SI). To ensure titratable residues are in their correct protonation state at non‐physiological pH conditions, titratable curves were determined following a protocol similar to ones previously reported.^[67]^ In brief, we employed hybrid non‐equilibrium MD and Monte Carlo (neMD/MC) simulations,[^68^, ^69^] implemented as a *namdcph* plugin designed for integration with NAMD ver. 2.12 (Figure S14 and Tables S11 and S12).^[70]^ Hydrogen atoms were then assigned based on the reaction pH (11.0; see Supporting Information for details). Illustrating reliability of our approach, the p*K*_a_ of Asp27, a residue proven to be crucial for the activity of the natural enzyme reaction, was estimated to be 6.350±0.002, whereas it was experimentally measured to be approximately 6.5.[^71^, ^72^, ^73^]

**Figure 6:**
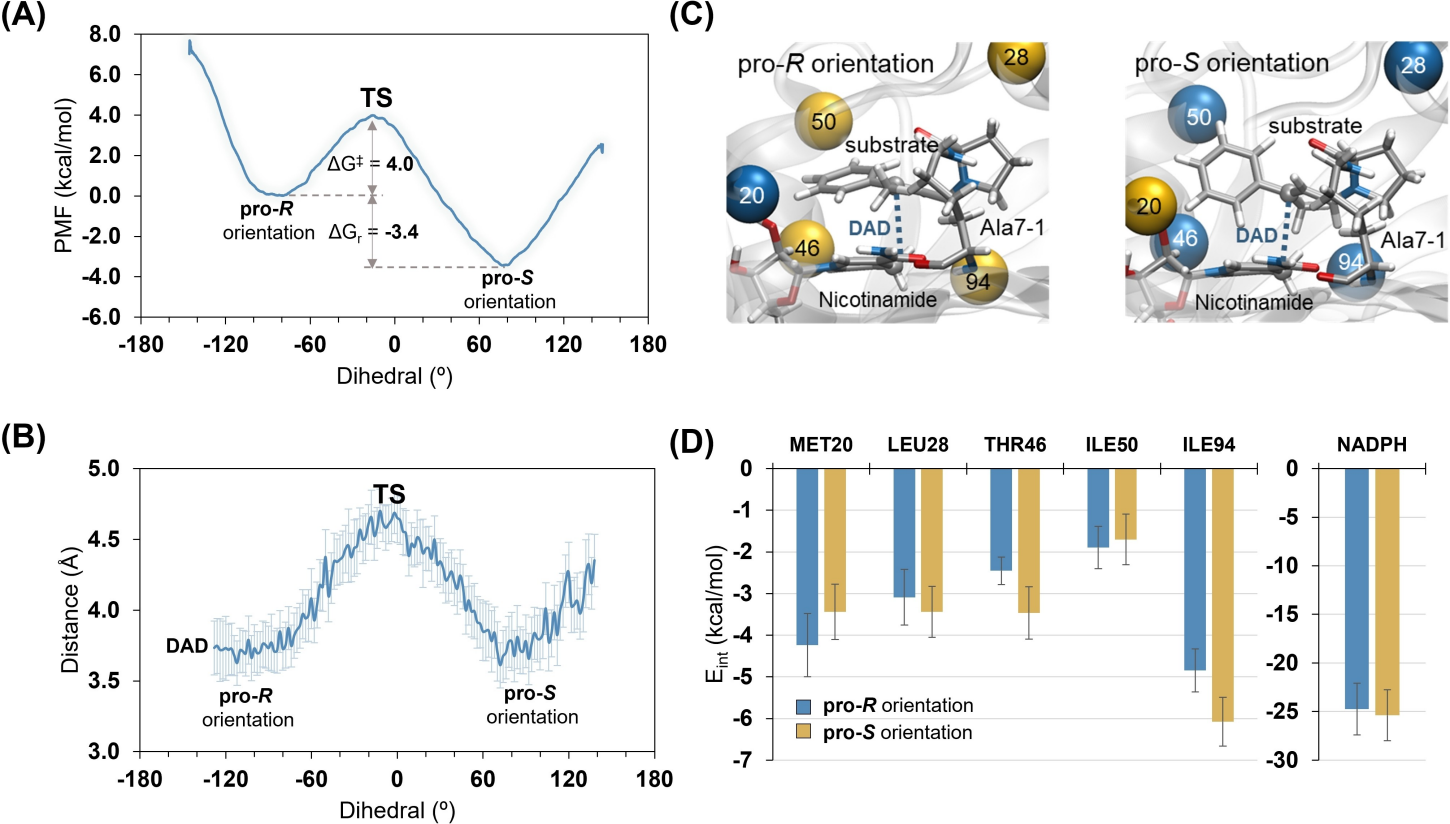
A) Free energy profile (potential of mean force, PMF, in kcal/mol) computed at AM1/MM level of theory for interconversion between the pro‐*R* and pro‐*S* orientation within the active site of **DHFR‐Ala7‐1**. B) Evolution of the donor‐acceptor distance (DAD) during substrate rotation. C) The relative position of NADPH cofactor and iminium intermediate in the active site of **DHFR‐Ala7‐1** illustrating pro‐*R* and pro‐*S* orientation used as initial geometries for 500 ns of unbiased MM MD simulations. D) Substrate‐protein interaction energies (electrostatic plus Lennard‐Jones; in kcal/mol) for residues located within 6 Å distance from the C_β_ of the substrate.

The relative positions of the iminium ion and NADPH during MD simulations were monitored based on the evolution of the dihedral angle that was defined between C4 carbon of the nicotinamide ring of NADPH, C_β_ and C_α_ of the iminium ion, and the carbon atom of the methyl group (CH_3_) attached to C_β_. Due to the nature of unbiased simulations, both the pro‐*R* and ‐*S* facing configurations are oscillating around the local minima (Figure S15). These observations suggested that both orientations are accessible and stable within the active site at pH 11.0, but their conversion requires overcoming a free energy barrier (Δ*G* ^≠^) higher than ~kT. Hence, we explored the interchange of the orientation using a hybrid quantum mechanics/molecular mechanics (QM/MM) scheme with the Umbrella Sampling (US) method (see Supporting Information for details).[^74^, ^75^] The free energy profile was constructed using the Weighted Histogram Analysis Method (WHAM)^[76]^ based on the 141 initial configurations obtained from the 100 ps QM/MM MD, in which values of the dihedral angle (as defined above) were restrained between −140 and 140° with interval inspections at every 2°. Our results showed that the pro‐*S* configuration is thermodynamically more stable than the pro‐*R* counterpart by 3.4 kcal/mol (Figure 6A), whereas the interchange between the pro‐*R* and ‐*S* orientation has an activation barrier of 4 kcal/mol. The donor‐acceptor distance (DAD) between the two reacting atoms (C4 of nicotinamide motif and C‐3 of iminium ion) always returned to its original value (3.6 Å) after rotation, as shown in Figures 6B,C and S15–S17 and the Supplementary movie clip, highlighting the suitability of the restrained dihedral angle.

While the likelihood of finding the pro‐*R* and ‐*S* facing configurations could be considered identical *a priori*, our results suggested that the pro‐*S* state is stabilized at pH 11.0 due to the overall improved energy interactions. In the pro‐*S* state, the iminium ion interacts strongly with Leu28, Thr46, Ile94 and the cofactor NADPH (Figure 6D). However, in the pro‐*R* orientation, only Met20 and Ile50 were found to establish strong interactions, thereby tipping the equilibrium towards the pro‐*S* interface for the hydride transfer reaction. Combined with a low energy barrier for the pro‐*R*→‐*S* conversion, our simulations suggested that the DHFR‐hosted reaction enantiomerically favors the formation of the *S* stereoisomer and hence agrees with the experimental findings.

**Figure 7:**
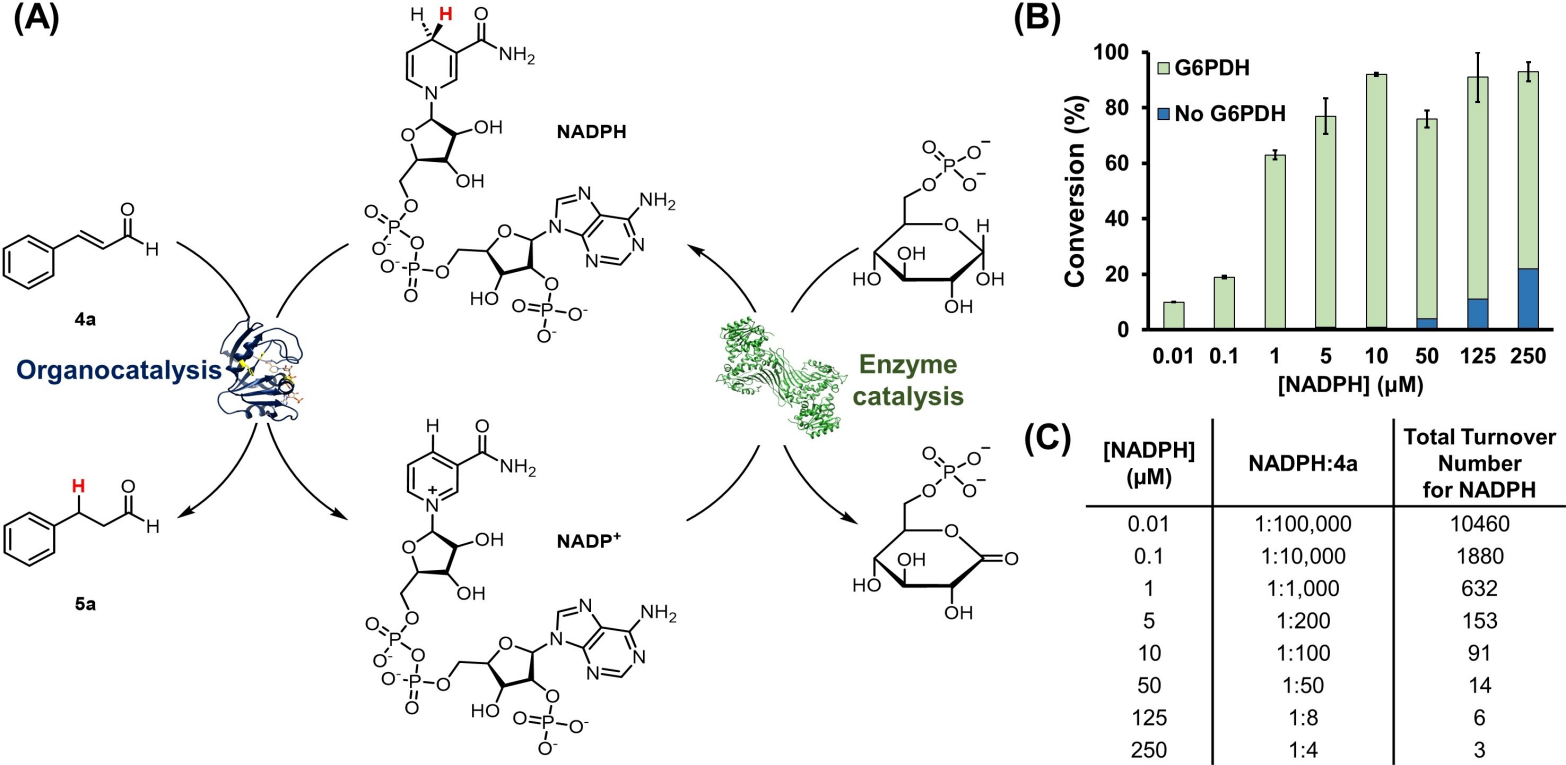
Coupling of the organocatalytic **DHFR‐Ala7‐1** and the enzymatic glucose‐6‐phosphate dehydrogenase (G6PDH) reactions. A) The organocatalytic transfer hydrogenation reaction of cinnamaldehyde **4 a** (1 mM) by **DHFR‐Ala7‐1** (10 mol %) was driven by the enzymatic G6PDH reaction (50 nM), which oxidizes glucose‐6‐phosphate (2 mM) to the corresponding lactone with the associated NADPH regeneration. B) Product conversions were estimated by a GC‐FID assay similar to previously described (see SI).^[79]^ C) The total turnover number for NADPH refers to the ratio of mole of product formed to mole of NADPH used. Each reaction was performed in triplicate and the mean reported.

To definitively illustrate that NADPH is recruited for secondary amine organocatalysis, the reaction catalyzed by **DHFR‐Ala7‐1** was integrated with a cofactor regeneration scheme recruiting glucose 6‐phosphate dehydrogenase (G6PDH), which oxidizes glucose 6‐phosphate by use of NADP^+^ (Figure 7A).^[77]^ Incorporation of this coupling reaction allowed the product conversion to reach up to 90 % with a 1 : 100 ratio of NADPH to **4 a**, whereas in the absence of G6PDH the conversion was barely detectable (<1 %; Figure 7B), strongly implying that the cofactor underwent consumption and regeneration. Indeed, maintaining the turnover number for NADPH (the ratio of product formed to NADPH used) at approximately 600 and below (Figure 7C and Table S8) resulted in significant product conversion (>60 %). However, a significant decrease in conversion (ca. 20 %) was observed when the NADPH:**4 a** ratio was extended to 1 : 10,000 ([NADPH]=0.1 μM), suggesting that the DHFR variant becomes destabilized in the absence of cofactor binding. Nevertheless, this experiment proved the concept that secondary amine organocatalysis, amongst other chemical catalytic systems,^[78]^ can be coupled with a well‐established biocatalysis tool.

## Conclusion

Secondary amines are excellent motifs for transforming protein templates into catalytically active entities, accelerating the creation of artificial enzymes. Traditional approaches are limited to a handful of protein templates including 4‐oxalcorotonate tautomerase (4‐OT) and streptavidin, which are based on the use of N‐terminal proline and modified biotin derivatives, respectively.[^19^, ^20^, ^21^, ^22^, ^23^, ^24^, ^25^] Conversely, the technique of genetic code expansion offers considerable flexibility in template selection and positioning. This approach enabled the introduction of secondary amines into alternative scaffolds, such as LmrR and DHFR, leading to the development of 11 catalytic systems with varying degrees of activity. As a proof of concept, an organocatalytic system with unique natural cofactor dependence has been promptly established. The DHFR‐based system demonstrates potentials for further refinement, such as expanding its substrate scope to include ketones, drawing parallels to the development of small molecule secondary amine organocatalysts.^[11]^ More importantly, considering the vast array of natural and engineered proteins with affinities for different ligands, our approach allows for testing over a wide range of reagents which are virtually unexplored in secondary amine catalysis, through enzyme design.

In nature, very few enzymes use secondary amines as catalytic motifs when compared to those recruiting primary amines (Lys and non‐proline N‐terminus). While 4‐OT employs its N‐terminal proline for acid‐base catalysis,[^3^, ^4^, ^5^] the N‐terminal proline in DNA‐formamidopyrimidine glycosylase acts as a nucleophile for iminium ion formation.[^6^, ^7^] In a similar context, UDP‐galactopyranose mutase and thymidylate synthase recruit the cofactors reduced flavin FADH_2_ and tetrahydrofolate, respectively, with their disubstituted nitrogen atoms serving as nucleophiles in iminium ion formation.[^8^, ^9^] Since secondary amine is measured to be more nucleophilic[^10^, ^11^, ^12^] and proposed to play roles in the prebiotic chemistry,[^16^, ^17^, ^18^] one might anticipate nature to have evolved more enzyme scaffolds with N‐terminal proline at their active sites or more cofactor chemistry based on the use of secondary amines. This scarcity may also attribute to its reactivity, as their prevalence in biological systems could lead to undesired side‐reactions with carbonyl metabolites, disrupting unrelated metabolic pathways. Since secondary amine incorporation could provide insights into natural enzyme evolution and biocatalyst design, the reported technique will be further used in both fundamental and applied research applications.

## Conflict of interests

The authors declare no conflict of interest.

1

## Supporting information

As a service to our authors and readers, this journal provides supporting information supplied by the authors. Such materials are peer reviewed and may be re‐organized for online delivery, but are not copy‐edited or typeset. Technical support issues arising from supporting information (other than missing files) should be addressed to the authors.

Supporting Information

Supporting Information

## Data Availability

The data that support the findings of this study are available in the supplementary material of this article.

## References

[1] M. J. Rodrigues , V. Windeisen , Y. Zhang , G. Guédez , S. Weber , M. Strohmeier , J. W. Hanes , A. Royant , G. Evans , I. Sinning , S. E. Ealick , T. P. Begley , I. Tews , Nat. Chem. Biol. 2017, 13, 290–294. 28092359 10.1038/nchembio.2273 PMC6078385

[2] J. Liang , Q. Han , Y. Tan , H. Ding , J. Li , Front. Mol. Biosci. 2019, 6, Article 4. 30891451 10.3389/fmolb.2019.00004 PMC6411801

[3] L. H. Chen , G. L. Kenyon , F. Curtin , S. Harayama , M. E. Bembenek , G. Hajipour , C. P. Whitman , J. Biol. Chem. 1992, 267, 17716–17721. 1339435

[4] H. S. Subramanya , D. I. Roper , Z. Dauter , E. J. Dodson , G. J. Davies , K. S. Wilson , D. B. Wigley , Biochemistry 1996, 35, 792–802. 8547259 10.1021/bi951732k

[5] C. P. Whitman , Arch. Biochem. Biophys. 2002, 402, 1–13. 12051677 10.1016/S0003-9861(02)00052-8

[6] J. Tchou , A. P. Grollman , J. Biol. Chem. 1995, 270, 11671–11677. 7744806 10.1074/jbc.270.19.11671

[7] O. M. Sidorkina , J. Laval , J. Biol. Chem. 2000, 275, 9924–9929. 10744666 10.1074/jbc.275.14.9924

[8] J. J. Tanner , L. Boechi , J. A. McCammon , P. Sobrado , Arch. Biochem. Biophys. 2014, 544, 128–141. 24096172 10.1016/j.abb.2013.09.017 PMC3946560

[9] M. S. Higgin , E. E. Carlson , T. D. Gruber , L. L. Kiessling , Nat. Struct. Mol. Biol. 2004, 11, 539–543. 15133501 10.1038/nsmb772

[10] E. Chamorro , M. Duque-Noreña , P. Pérez , J. Mol. Struct. 2009, 896, 73–79.

[11] A. Erkkila , I. Majander , P. M. Pihko , Chem. Rev. 2007, 107, 5416–5470. 18072802 10.1021/cr068388p

[12] F. Brotzel , Y. C. Chu , H. Mayr , J. Org. Chem. 2007, 72, 3679–3688. 17411095 10.1021/jo062586z

[13] D. W. MacMillan , Nature 2008, 455, 304–308. 18800128 10.1038/nature07367

[14] Y. Q. Zou , F. M. Hormann , T. Bach , Chem. Soc. Rev. 2018, 47, 278–290. 29155908 10.1039/c7cs00509a PMC5789435

[15] S. H. Xiang , B. Tan , Nat. Commun. 2020, 11, 3786. 32728115 10.1038/s41467-020-17580-z PMC7391739

[16] V. Kubyshkin , N. Budisa , Int. J. Mol. Sci. 2019, 20, 5507. 31694194 10.3390/ijms20215507 PMC6862034

[17] A. B. Northrup , D. W. MacMillan , Science 2004, 305, 1752–1755. 15308765 10.1126/science.1101710

[18] J. E. Hein , D. G. Blackmond , Acc. Chem. Res. 2012, 45, 2045–2054. 22353168 10.1021/ar200316n

[19] E. Zandvoort , B. J. Baas , W. J. Quax , G. J. Poelarends , ChemBioChem 2011, 12, 602–609. 21290551 10.1002/cbic.201000633

[20] E. Zandvoort , E. M. Geertsema , W. J. Quax , G. J. Poelarends , ChemBioChem 2012, 13, 1274–1277. 22615135 10.1002/cbic.201200225

[21] C. Guo , M. Saifuddin , T. Saravanan , M. Sharifi , G. J. Poelarends , ACS Catal. 2019, 9, 4369–4373. 31080691 10.1021/acscatal.9b00780 PMC6503466

[22] L. Biewenga , T. Saravanan , A. Kunzendorf , J. Y. V. D. Meer , T. Pijning , P. G. Tepper , R. V. Merkerk , S. J. Charnock , A. M. W. H. Thunnissen , G. J. Poelarends , ACS Catal. 2019, 9, 1503–1513. 30740262 10.1021/acscatal.8b04299 PMC6366683

[23] A. R. Nödling , K. Świderek , R. Castillo , J. W. Hall , A. Angelastro , L. C. Morrill , Y. Jin , Y.-H. Tsai , V. Moliner , L. Y. P. Luk , Angew. Chem. Int. Ed. 2018, 57, 12478–12482. 10.1002/anie.201806850 PMC6531919 30027571

[24] N. Santi , L. C. Morrill , L. Y. P. Luk , Molecules 2020, 25, 2457. 32466220 10.3390/molecules25102457 PMC7287710

[25] N. Santi , L. C. Morrill , K. Swiderek , V. Moliner , L. Y. P. Luk , Chem. Commun. 2021, 57, 1919–1922. 10.1039/d0cc08142f PMC8330412 33496282

[26] G. Xu , M. Crotti , T. Saravanan , K. M. Kataja , G. J. Poelarends , Angew. Chem. Int. Ed. 2020, 59, 10374–10378. 10.1002/anie.202001373 PMC7317984 32160395

[27] C. M. Dundas , D. Demonte , S. Park , Appl. Microbiol. Biotechnol. 2013, 97, 9343–9353. 24057405 10.1007/s00253-013-5232-z

[28] Q. Le , V. Nguyen , S. Park , Appl. Microbiol. Biotechnol. 2019, 103, 7355–7365. 31372706 10.1007/s00253-019-10036-5

[29] C. Mayer , C. Dulson , E. Reddem , A. W. H. Thunnissen , G. Roelfes , Angew. Chem. Int. Ed. 2019, 58, 2083–2087. 10.1002/anie.201813499 PMC6519144 30575260

[30] I. Drienovska , C. Mayer , C. Dulson , G. Roelfes , Nat. Chem. 2018, 10, 946–952. 29967395 10.1038/s41557-018-0082-z

[31] Z. Zhou , G. Roelfes , Nat. Catal. 2020, 3, 289–294.

[32] R. B. L. Gower , Z. Zhou , I. Drienovská , G. Roelfes , ACS Catal. 2021, 11, 6763–6770. 34168902 10.1021/acscatal.1c00996 PMC8218303

[33] A. J. Burke , S. L. Lovelock , A. Frese , R. Crawshaw , M. Ortmayer , M. Dunstan , C. Levy , A. P. Green , Nature 2019, 570, 219–223. 31132786 10.1038/s41586-019-1262-8

[34] R. Crawshaw , A. E. Crossley , L. Johannissen , A. J. Burke , S. Hay , C. Levy , D. Baker , S. L. Lovelock , A. P. Green , Nat. Chem. 2022, 14, 313. 34916595 10.1038/s41557-021-00833-9 PMC7612480

[35] C. R. Polycarpo , S. Herring , A. Berube , J. L. Wood , D. Soll , A. Ambrogelly , FEBS Lett. 2006, 580, 6695–6700. 17126325 10.1016/j.febslet.2006.11.028 PMC1817836

[36] A. G. Scheuch , E. Bonandi , I. Drienovská , ChemCatChem 2024, 16, e202301004.

[37] T. T. Williams , Y. H. Tsai , L. Y. P. Luk , Research Square 2021, DOI: 10.21203/rs.3.rs-468406/v2.

[38] A. R. Nodling , L. A. Spear , T. L. Williams , L. Y. P. Luk , Y. H. Tsai , Essays Biochem. 2019, 63, 237–266. 31092687 10.1042/EBC20180042 PMC6610526

[39] D. P. Nguyen , T. Elliott , M. Holt , T. W. Muir , J. W. Chin , J. Am. Chem. Soc. 2011, 133, 11418–11421. 21736333 10.1021/ja203111c

[40] T. L. Williams , D. J. Iskandar , A. R. Nödling , Y. Tan , L. Y. P. Luk , Y. H. Tsai , Amino Acids 2021, 53, 89–96. 33331978 10.1007/s00726-020-02927-z PMC7822784

[41] H. Agustiandari , J. Lubelski , H. B. van den Berg van Saparoea , O. P. Kuipers , A. J. M. Driessen , J. Bacteriol. 2008, 190, 759–763. 17993533 10.1128/JB.01151-07 PMC2223683

[42] G. Roelfes , Acc. Chem. Res. 2019, 52, 545–556. 30794372 10.1021/acs.accounts.9b00004 PMC6427492

[43] J. Bos , F. Fusetti , A. J. M. Driessen , G. Roelfes , Angew. Chem. Int. Ed. 2012, 51, 7472–7475. 10.1002/anie.201202070 22707314

[44] I. Drienovska , L. Alonso-Cotchico , P. Vidossich , A. Lledos , J. D. Marechal , G. Roelfes , Chem. Sci. 2017, 8, 7228–7235. 29081955 10.1039/c7sc03477f PMC5633786

[45] R. B. L. Gower , C. Mayer , G. Roelfes , Nat. Chem. Rev. 2019, 3, 687–705.

[46] S. Cattaneo , S. J. Freakley , D. J. Morgan , M. Sankar , N. Dimitratos , G. J. Hutchings , Catal. Sci. Technol. 2018, 8, 1677–1685.

[47] S. G. Ouellet , J. B. Tuttle , D. W. C. MacMillan , J. Am. Chem. Soc. 2005, 127, 32–33. 15631434 10.1021/ja043834g

[48] A. P. Brogan , T. J. Dickerson , K. D. Janda , Chem. Commun. 2007, 4952–4954. 10.1039/b713273e 18361380

[49] S. B. Lara , Z. Liu , A. Habtemariam , A. M. Pizarro , B. Qamar , P. J. Sadler , Angew. Chem. Int. Ed. 2012, 51, 3897–3900. 10.1002/anie.201108175 22415924

[50] Y. Maenaka , T. Suenobu , S. Fukuzumi , J. Am. Chem. Soc. 2012, 134, 9417–9427. 22577897 10.1021/ja302788c

[51] Y. Maenaka , T. Suenobu , S. Fukuzumi , J. Am. Chem. Soc. 2012, 134, 367–374. 22122737 10.1021/ja207785f

[52] Y. Okamoto , V. Köhler , T. R. Ward , J. Am. Chem. Soc. 2016, 138, 5781–5784. 27100673 10.1021/jacs.6b02470

[53] A. D. Liang , J. S. Plana , R. L. Peterson , T. R. Ward , Acc. Chem. Res. 2019, 52, 585–595. 30735358 10.1021/acs.accounts.8b00618 PMC6427477

[54] L. Y. P. Luk , J. Ruiz-Pernía , W. M. Dawson , M. Roca , E. J. Loveridge , D. R. Glowacki , J. N. Harvey , A. J. Mulholland , I. Tuñón , V. Moliner , R. K. Allemann , Proc. Natl. Acad. Sci. USA 2013, 110, 16344–16349. 24065822 10.1073/pnas.1312437110 PMC3799346

[55] C. A. Fierke , K. A. Johnson , S. J. Benkovic , Biochemistry 1987, 26, 4085–4092. 3307916 10.1021/bi00387a052

[56] Z. Zhang , P. T. Rajagopalan , T. Selzer , S. J. Benkovic , G. G. Hammes , Proc. Natl. Acad. Sci. USA 2004, 101, 2764–2769. 14978269 10.1073/pnas.0400091101 PMC371724

[57] G. P. Miller , S. J. Benkovic , Biochemistry 1998, 37, 6336–6342. 9572848 10.1021/bi973065w

[58] L. Y. P. Luk , E. J. Loveridge , R. K. Allemann , Phys. Chem. Chem. Phys. 2015, 17, 30817–30827. 25854702 10.1039/c5cp00794a

[59] T. Knaus , C. E. Paul , C. W. Levy , S. de Vries , F. G. Mutti , F. Hollmann , N. S. Scrutton , J. Am. Chem. Soc. 2016, 138, 1033–1039. 26727612 10.1021/jacs.5b12252 PMC4731831

[60] A. Guarneri , A. H. Westphal , J. Leertouwer , J. Lunsonga , M. C. R. Franssen , D. J. Opperman , F. Hollmann , W. J. H. van Berkel , C. E. Paul , ChemCatChem 2020, 12, 1368–1375.

[61] M. Jeschek , R. Reuter , T. Heinisch , C. Trindler , J. Klehr , S. Panke , T. R. Ward , Nature 2016, 537, 661–665. 27571282 10.1038/nature19114

[62] F. Schwizer , Y. Okamoto , T. Heinisch , Y. Gu , M. M. Pellizzoni , V. Lebrun , R. Reuter , V. Köhler , J. C. Lewis , T. R. Ward , Chem. Rev. 2018, 118, 142–231. 28714313 10.1021/acs.chemrev.7b00014

[63] M. R. Sawaya , J. Kraut , Biochemistry 1997, 36, 586–603. 9012674 10.1021/bi962337c

[64] V. Stojkovic , L. L. Perissinotti , D. Willmer , S. J. Benkovic , A. Kohen , J. Am. Chem. Soc. 2012, 134, 1738–1745. 22171795 10.1021/ja209425w PMC4341912

[65] K. Swiderek , A. R. Nodling , Y. H. Tsai , L. Y. P. Luk , V. Moliner , J. Phys. Chem. A 2018, 122, 451–459. 29256614 10.1021/acs.jpca.7b11803 PMC5785706

[66] D. Sokolova , K. Tiefenbacher , RSC Adv. 2021, 11, 24607–24612. 34354825 10.1039/d1ra04333a PMC8278068

[67] K. Świderek , S. Velasco-Lozano , M. À. Galmés , I. Olazabal , H. Sardon , F. López-Gallego , V. Moliner , Nat. Commun. 2023, 14, 3556. 37321996 10.1038/s41467-023-39201-1 PMC10272158

[68] J. Mongan , D. A. Case , J. A. McCammon , J. Comput. Chem. 2004, 25, 2038–2048. 15481090 10.1002/jcc.20139

[69] B. K. Radak , C. Chipot , D. Suh , S. Jo , W. Jiang , J. C. Phillips , K. Schulten , B. Roux , J. Chem. Theory Comput. 2017, 13, 5933–5944. 29111720 10.1021/acs.jctc.7b00875 PMC5726918

[70] J. C. Phillips , R. Braun , W. Wang , J. Gumbart , E. Tajkhorshid , E. Villa , C. Chipot , R. D. Skeel , L. Kalé , K. Schulten , J. Comput. Chem. 2005, 26, 1781–1802. 16222654 10.1002/jcc.20289 PMC2486339

[71] Q. Wan , B. C. Bennett , M. A. Wilson , A. Kovalevsky , P. Langan , E. E. Howell , C. Dealwis , Proc. Natl. Acad. Sci. USA 2014, 111, 18225–18230. 25453083 10.1073/pnas.1415856111 PMC4280638

[72] R. L. Blakley , J. R. Appleman , J. H. Freisheim , M. J. Jablonsky , Arch. Biochem. Biophys. 1993, 306, 501–509. 8105754 10.1006/abbi.1993.1543

[73] E. E. Howell , J. E. Villafranca , M. S. Warren , S. J. Oatley , J. Kraut , Science 1986, 231, 1123–1128. 3511529 10.1126/science.3511529

[74] B. Roux , Comput. Phys. Commun. 1995, 91, 275–282.

[75] G. M. Torrie , J. P. Valleau , J. Comput. Phys. 1977, 23, 187–199.

[76] S. Kumar , J. M. Rosenberg , D. Bouzida , R. H. Swendsen , P. A. Kollman , J. Comput. Chem. 1992, 13, 1011–1021.

[77] C. H. Wong , G. M. Whitesides , J. Am. Chem. Soc. 2002, 103, 4890–4899.

[78] V. Köhler , Y. M. Wilson , M. Dürrenberger , D. Ghislieri , E. Churakova , T. Quinto , L. Knörr , D. Häussinger , F. Hollmann , N. J. Turner , T. R. Ward , Nat. Chem. 2013, 5, 93–99. 23344429 10.1038/nchem.1498

[79] D. Sandner , U. Krings , R. G. Berger , Z. Naturforsch. C .J. Biosci. 2018, 73, 67–75. 29145172 10.1515/znc-2017-0087

