# Supplementary material for: Secondary Amine Catalysis in Enzyme Design: Broadening Protein Template Diversity through Genetic Code Expansion
Source: Angew Chem Int Ed Engl. 2024 Apr 19;63(22):e202403098. doi: 10.1002/anie.202403098 (PMC11497281; doi:10.1002/anie.202403098)
Supplement: Supplementary file 1 — Supporting Information [file ANIE-63-e202403098-s002.pdf]

Angewandte Chemie
Eine Zeitschrift der Gesellschaft Deutscher Chemiker

Supporting Information

Secondary Amine Catalysis in Enzyme Design: Broadening Protein Template Diversity through Genetic Code Expansion

T. L. Williams, I. M. Taily, L. Hatton, A. A Berezin, Y.-L. Wu, V. Moliner, K. Świderek, Y.-H. Tsai*, L. Y. P. Luk**

Supplementary Information

Secondary Amine Catalysis in Enzyme Design: Broadening Protein Template Diversity through Genetic Code Expansion

Thomas L Williams,[a], # Irshad M Taily,[a], # Lewis Hatton,[a] Andrey A Berezin,[a] Yi-Lin Wu,[a] Vicent Moliner,[b] Katarzyna Świderek,*[b] Yu-Hsuan Tsai,*[c] Louis Y P Luk.*[a]

[a] School of Chemistry and Cardiff Catalysis Institute
Cardiff University, Main Building, Park Place
Cardiff, CF10 3AT
E-mail: lukly@cardiff.ac.uk

[b] Prof. Vicent Moliner, Dr Katarzyna Świderek
BioComp Group, Institute of Advanced Materials (INAM),
Universitat Jaume I
12071 Castelló, Spain
E-mail: swiderek@uji.es

[c] Dr Yu-Hsuan Tsai
Institute of Molecular Physiology
Shenzhen Bay Laboratory
Gaoke International Innovation Center
Guangming District, 518132 Shenzhen
Guangdong, China
E-mail: tsai.y-h@outlook.com

[#] These authors contributed equally to this work.

Table of Contents

1. Reagents and materials 2
2. Molecular cloning 3
3. Protein expression and purification 5
4. Protein mass spectra 8
5. Analytical size exclusion chromatography of LmrR variants (y-axis is A_{210} and x-axis is retention time in minutes) 8
6. Catalytic activity screening reactions 10
7. Iminium ion intermediate characterization 14
8. Kinetic evaluations 17
9. NADPH hydride transfer selectivity characterization 18
10. Kinetic isotope effect study 20
11. Substrate scope 21
12. Enantioselectivity Assay 23
13. Cofactor recycling assay 27
14. Analytical chemistry 28
15. Nucleotide and amino acid sequences 29
16. Computational Studies 33
17. NMR spectroscopy and Mass spectrometry 41
18. References 44

1. Reagents and materials

(*E*)-3-Phenylbut-2-enal **4i** was synthesized according to the reported method;[1] the other α,β-unsaturated aldehyde and ketone substrates, as well as compound **5a**, were purchased from commercial retailers [*trans*-cinnamaldehyde **4a** (Fluorochem, Catalog #F224150), *trans*-*p*-chlorocinnamaldehyde **4b** (Fluorochem, Catalog #F324046), *trans*-4-fluorocinnamaldehyde **4c** (Merck, Catalog #683027), *trans*-4-bromocinnamaldehyde **4d** (Merck, Catalog #683019), *trans*-4-methoxycinnamaldehyde **4e** (Acros Organics, Catalog #AC351710250), (*E*)-3-(p-tolyl)acrylaldehyde **4f** (Fluorochem, Catalog #F224820), *trans*-4-nitrocinnamaldehyde **4g** (Merck, Catalog #281670), (3*E*)-4-(4-chlorophenyl)but-3-en-2-one **4h** (Fluorochem, Catalog #F096130) and 3-phenylbutyraldehyde **5a** (Fluorochem, Catalog #F520893)]. NADPH tetrasodium salt was purchased from Apollo Scientific (Catalog #BIB3014). $NADP^+$ disodium salt was purchased from Santa Cruz Biotechnology (Catalog #205763). Glucose-6-phosphate dehydrogenase (G6PDH) from *L. mentroides* (Catalog #G8259) and glucose-6-phosphate (Catalog #10127647001) were purchased from Merck. Isopropanol-D8 was purchased from Acros Organics (Catalog #174850250). Synthesis of amino acids **1**, **2**[2] and, **3**,[3] BNAH[4] and [4*R*-^{2}H]-NADPH (NADPD)[5] were performed as previously reported. Gibson Assembly reactions were performed using NEBuilder HiFi DNA assembly (Catalog #E2621) following the manufacturer's protocol. Site-directed mutagenesis reactions were performed following the QuikChange Site-Directed Mutagenesis manufacturer's protocol (Catalog #200519). PCR reactions were performed using PrimeSTAR Max from Takara Bio (Catalog #R045A). Oligonucleotide primers were purchased from Merck, and their sequences are shown in **Table S1**. Protein extinction coefficients were estimated using ProtParam (Expasy).

2. Molecular cloning

M. bakeri PylRS/tRNA plasmids

The plasmid encoding MbPylRS and Pyl-tRNA was a kind gift from Jason Chin.[6] The ThzKRS plasmid was constructed through introducing four mutations by PCR into the MbPylRS plasmid. Introduction of the mutation Asp267Ser was made using primers P1 and P2, and the mutations Cys313Val, Met315Phe and Asp344Gly were made using primers P3 and P4.[3]

Expression plasmids

The sfGFP Asn150TAG plasmid is available from Addgene (#133455).[7]

The gene encoding the LmrR protein was purchased as a double stranded DNA fragment from GeneArt (Life Technologies). The gene was codon optimized for expression in *E. coli* and contained two mutations, Lys55Asn and Lys59Asp, from the wild-type sequence. The gene was synthesized with an additional C-terminus hexa-histidine tag and 20 base pair overhangs at the 5' and 3' ends complimentary to NcoI and BamHI of a linearized pET-28a vector. The gene was integrated into the linearized vector using the Gibson Assembly. Each TAG mutant was generated through QuikChange PCR reactions. For the Val15TAG mutant, primers used were P5 and P6. For the Asp19TAG mutant, primers P7 and P8 were used. For the Met89TAG mutant, primers P9 and P10 were used. For the Phe93TAG mutant, primers P11 and P12 were used.

The gene encoding the wild-type *E. coli* DHFR was a kind gift from Rudolf Allemann.[8] The gene was amplified by PCR using primers P13 and P14 and simultaneously a C-terminal hexa-histidine tag and 20 base pair overhangs complimentary to NcoI and BamHI of a linearized pET28a vector were added. The gene was cloned into the linearized vector using the Gibson Assembly. Introduction of the TAG mutations was performed using QuikChange PCR reactions. For the Ala7TAG mutant, primers P15 and P16 were used. For the Phe31TAG mutant, primers P17 and P18 were used. For the Ser49TAG mutant, primers P19 and P20 were used. For Ala7TAG Y100F, Ala7 Y100E, Ala7 D27N and Ala7 D27A mutants, primers (P21, 22), (P23, 22), (P24, 25) and (P26, 25) were used respectively.

The expression plasmid for the enzyme TbADH used in the synthesis of [4*R*-^{2}H]-NADPH was a kind gift from Rudolf Allemann.[5]

Table S1. Oligonucleotide primers

**P1**	CCGACCCTGTATAACTATCTGCGTAAACTGGATCG
**P2**	GTTATACAGGGTCGGGCTCAGCATCGGACGCAGG
**P3**	GCGCTGATCAAAGAATTCCTGGATTATCTGGAAATCGACTTCGAAATTGTGGGCGGTAGCTGCATGGTGTATGG
**P4**	AGGAATTCTTTGATCAGCGCTTCCAGGTTTTCACGGGTGCAGCCGCTGCCAAATTGCACAAAGTTAACCATGGTGAATTCTTCCAG
**P5**	TGCGTGCTCAAACCAATTAGATCCTGCTGAATGTCCTGAAAC
**P6**	AACCAATGTCATCCTGCTGTAGGTCCTGAAACAAGGCGATAAC
**P7**	TGCCATTACCACATAGTTATCGCCTTGTTTTCAGG
**P8**	TCGCCTTGTTTCAGGACATTCAGCAGG
**P9**	ATGAAAACTAGCGCCTGGCGTTCGAATCC
**P10**	ACTCCCAGGATTCGAACGCCAGG
**P11**	TGCGCCTGGCGTAGGAATCCTGGAGTCGTGTGG
**P12**	TCAATGATTTTGTCCACACGACTCCAGG
**P13**	GTTTAACTTTAAGAAGGAGATATACATATGATCAGTCTGATTGCGGCGTTAGC
**P14**	CGGAGCTCGAATTCGTTAGCCGCTGCTGTGATGATGATGATGATGGCTGCTGCCCCGCCGCTCCAGAATCTCAAAGC
**P15**	TACATATGATCAGTCTGATTGCGTAGTTAGCGGTAG
**P16**	CGCAATCAGACTGATCATATGTATATCTCC
**P17**	TCGCCTGGTAGAAACGCAACACCTTAAATAAACC
**P18**	CCATAATCACGGGTTTATTTAAGGTGTTGC
**P19**	TTATGGGCCGCCATACCTGGGAATAGATCGGTCGTCC
**P20**	AGGTATGGCGGCCCATAATCACGG
**P21**	TGGTGATTGGCGGCGGTCGCGTTTTCGAACAGTTCTTG
**P22**	ACCGCCGCCAATCACCATGATTTCTGGTAC
**P23**	TGGTGATTGGCGGCGGTCGCGTTGAAGAACAGTTCTTG
**P24**	ATGCCGTGGAACCTGCCTGCCAACCTCGCCTGGTTTAAAC
**P25**	AGGTTCCACGGCATGGCGTTTTCCATG
**P26**	ATGCCGTGGAACCTGCCTGCCGCCCTCGCCTGGTTTAAAC

3. Protein expression and purification

To test the incorporation of the unnatural amino acids (**1**, **2** and **3**), the sfGFP Asn150TAG plasmid was co-transformed with either the MbPylRS or ThzKRS plasmid into chemically competent *E. coli* BL21(DE3) cells and recovered for 1 hour at 37 °C in 1 mL of LB media with constant agitation (200 rpm). Following previously reported procedure,[6] 200 µL of the culture was used to inoculate a 10 mL starter culture of LB media supplemented with kanamycin (37.5 µg/mL) and spectinomycin (50 µg/mL). The starter culture was incubated overnight at 37 °C with constant agitation. On the following day, 100 µL of the starter culture was used to inoculate 10 mL of LB media supplemented with kanamycin (37.5 µg/mL) and spectinomycin (50 µg/mL); this culture was incubated at 37 °C until it reached an OD_{600} of 0.6-0.8. IPTG was added to reach a final concentration 0.5 mM and the culture was then split into two 5 mL aliquots, with one aliquot added with 1.0 mM of the required unnatural amino acid, whereas the other served as a negative control and was left without the addition of the amino acid. The cultures were then incubated at 20 °C overnight. The following day the cultures were normalized to an OD_{600} of 1.0 and unnatural amino acid incorporation was determined by SDS-PAGE analysis (**Fig. S1**).

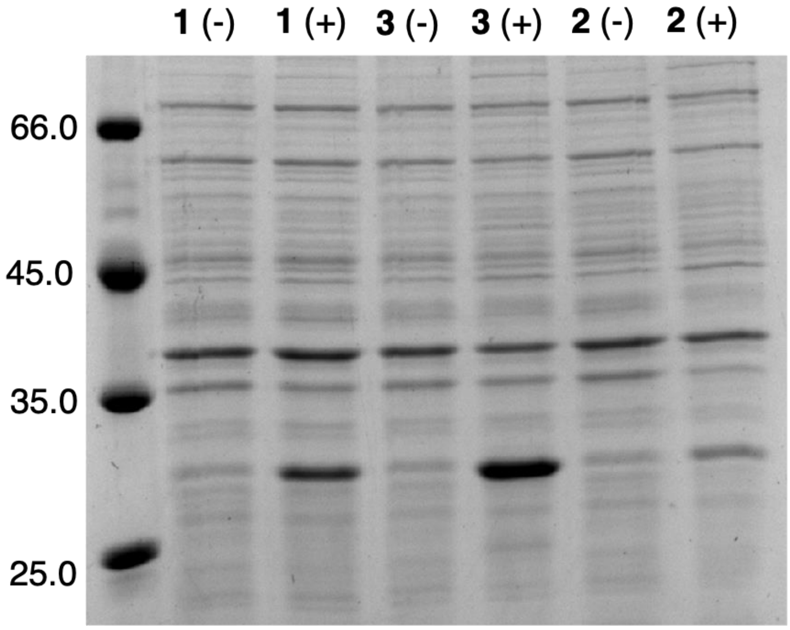

Figure S1. SDS-PAGE analysis of cell lysates containing an orthogonal synthetase/tRNA plasmid and plasmid sfGFP Asp150TAG cultured in the presence (+) or absence (-) of 1 mM UAA **1**, **2** or **3**. UAA **1** was incorporated using plasmid MbPylRS, **2** and **3** using the plasmid ThzKRS. Following overnight expression samples were normalized to OD_{600} of 1.0. Protein ladder in kDa. Mass of full length sfGFP is approximately 27 kDa.

For LmrR, the plasmid was transformed into chemically competent *E. coli* BL21(DE3). The cells were recovered for 1 hour at 37 °C in 1 mL of LB media with constant shaking (200 rpm) and then 200 µL was used to inoculate 10 mL of LB media supplemented with kanamycin (37.5 µg/mL). The starter culture was incubated overnight at 37 °C with constant shaking. On the following day, the starter culture was diluted into 1 L of fresh LB media supplemented with kanamycin (37.5 µg/mL). The culture was incubated at 37 °C until it reached an OD_{600} of 0.8-1.0 at which point gene expression was induced by the addition of 0.5 mM of IPTG. The cells were then cultured at 30 °C overnight and harvested by centrifugation (20,000 rcf, 4 °C, 30 min). The pellet was resuspended in 25 mL phosphate buffer (50 mM NaP_i, 150 mM NaCl, pH 8.0) and added with 1.0 µg/mL DNase, 100 µg/mL $MgCl_2$ and 5.0 µg/mL PMSF. The resuspended cells were lysed by sonication on ice. The lysed cells were centrifuged to separate the pellet (27,000 rcf, 4 °C, 30 min) and the supernatant passed through 0.22 µm syringe filters. The filtered supernatant was applied to a Ni-NTA column equilibrated in the above buffer, which was washed twice with 30 mL wash buffer (50 mM NaP_i, 150 mM NaCl, 20 mM imidazole, pH 8.0). The

protein was eluted in elution buffer (50 mM NaP_i, 150 mM NaCl, 300 mM imidazole, pH 8.0), and the eluent was dialyzed overnight into the reaction buffer (50 mM NaP_i, 150 mM NaCl, pH 7.0) at 4 °C. The protein was then concentrated with 10 kDa cut-off centrifugal concentrators and its concentration was determined using a NanoDrop instrument by measuring the UV absorbance 280 nm with the extinction coefficient of 19940 M^{-1} cm^{-1} for one LmrR monomer. Purified proteins were stored in the reaction buffer at -80 °C.

To prepare LmrR variants incorporated with the unnatural amino acid, chemically competent *E. coli* BL21(DE3) cells were co-transformed with the relevant LmrR plasmid and either the MbPylRS plasmid or ThzKRS plasmid. In addition to kanamycin, spectinomycin (50 µg/mL) was added to each starter and expression culture. Upon induction with IPTG, the relevant unnatural amino acid was added to a final concentration of 1 mM. The variants were purified as the wild-type protein. Each protein was analyzed by SDS-PAGE (**Fig. S2**) and unnatural amino acid incorporation determined by mass spectrometry (**Fig. 2**). Dimer formation was analyzed by size exclusion chromatography (SI, Pg S8-S10).

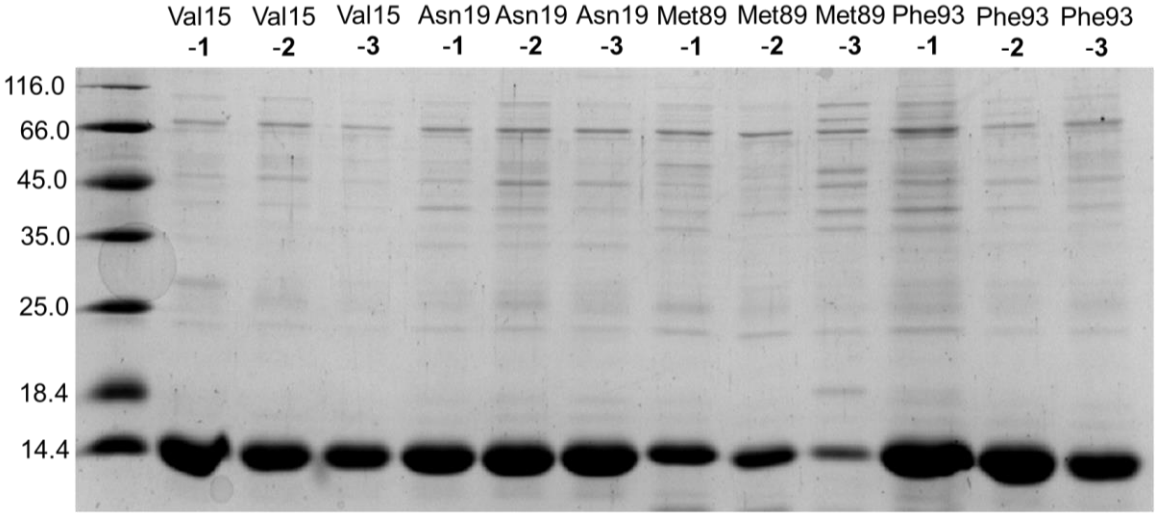

Figure S2. SDS-PAGE analysis of LmrR TAG variants isolated by Ni-NTA chromatography. Protein ladder is shown in kDa. Mass of a LmrR monomer is approximately 14 kDa.

For DHFR, the plasmid was transformed into chemically competent *E. coli* BL21(DE3) cells. The cells were recovered by incubating for 1 hour at 37 °C in 1 mL of LB media with constant shaking (200 rpm) and then 200 µL was used to inoculate 10 mL of LB media supplemented with kanamycin (37.5 µg/mL). The starter culture was incubated overnight at 37 °C with constant shaking. The following day the starter culture was diluted into 1 L of fresh LB media supplemented with kanamycin (37.5 µg/mL). The culture was incubated at 37 °C until it reached an OD_{600} of 0.6-0.8 at which point gene expression was induced by adding 0.5 mM of IPTG. After shaking at 20 °C 200 rpm overnight, the cells were harvested by centrifugation (5,000 rcf, 4 °C, 30 min). The dry pellet was resuspended in 25 mL phosphate buffer (50 mM NaP_i, 150 mM NaCl, pH 8.0) and added with 5 µg/mL PMSF. The resuspended cells were lysed by sonication on ice and centrifuged at 27,000 rcf (4 °C, 30 min) yielding the supernatant which was then passed through 0.22 µm syringe filters. The filtered supernatant was applied to a Ni-NTA column equilibrated in the above phosphate buffer. The column was washed twice with 30 mL of wash buffer (50 mM NaP_i, 150 mM NaCl, 20 mM imidazole, pH 8.0) and then the protein was eluted in elution buffer (50 mM NaP_i, 150 mM NaCl, 300 mM imidazole, pH 8.0). After dialysis at 4 °C overnight in the reaction buffer (50 mM NaP_i, 150 mM NaCl, pH 7.0), the protein was concentrated with 10 kDa cut-off centrifugal concentrators. The protein concentration was determined with a NanoDrop instrument by using an extinction coefficient of 33585 M^{-1} cm^{-1} at 280 nm. Purified proteins were stored in the reaction buffer at -80 °C.

For the DHFR TAG mutants, the transformation was achieved by co-transforming the relevant DHFR plasmid with the MbPylRS plasmid. In addition to kanamycin, spectinomycin (50 µg/mL) was added to each starter and expression culture

and, upon induction with IPTG, unnatural amino acid **1** was added to a final concentration of 1 mM. The variants were purified as the wild-type protein. Each protein was analyzed by SDS-PAGE (**Fig. S3**) and unnatural amino acid incorporation determined by mass spectrometry (**Fig. S4**).

Following the preliminary screening reactions, **LmrR-Phe93-1** and **DHFR-Ala7-1** were further purified using a size exclusion fast protein liquid chromatography (FPLC) setup. The FPLC was performed on an ÄKTA purifier (GE Healthcare) system at room temperature using a GE Healthcare BSD75 10/300 SEC column in PBS (50 mM NaP_i, 150 mM NaCl, pH 7.0). Protein elution was monitored by UV absorbance at 280 nm. Fractions containing **DHFR-Ala7-1** were combined and concentrated using a 10 kDa cut off centrifugal concentrator (Millipore). Protein concentration was determined using nanodrop. Purified proteins were stored in the reaction buffer at -80 °C.

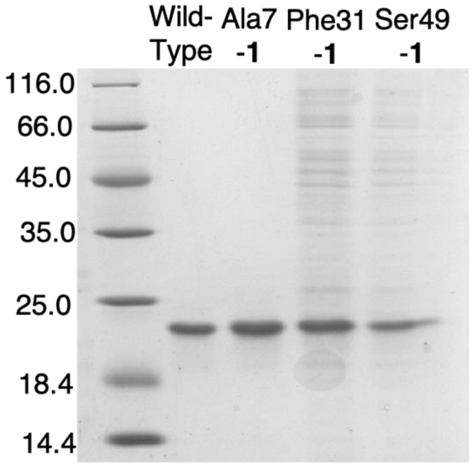

Figure S3. SDS-PAGE analysis of DHFR and variants isolated by Ni-NTA chromatography. Protein ladder is shown in kDa. Mass of DHFR is approximately 19 kDa.

Expression and purification of TbADH was performed as previously reported and was confirmed by SDS-PAGE analysis.[5]

4. Protein mass spectra

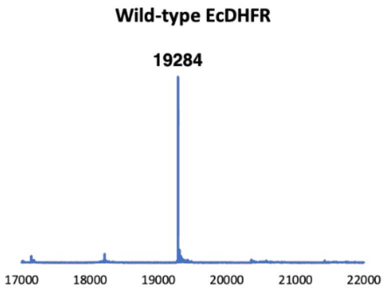

Mutant	Calculated mass (g/mol)
Ala7-1	19439
Phe31-1	19363
Ser49-1	19424

Calculated mass of wild-type EcDHFR – N-terminal Met = 19284 g/mol

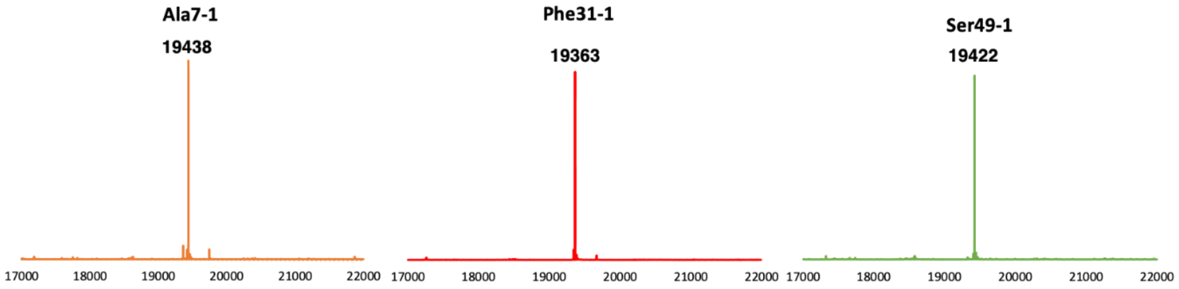

Figure S4. Deconvoluted ESI mass spectra for each DHFR variant (with methionine included). There were no profound peaks that correspond to the hydrolysis of the D-proline.

5. Analytical size exclusion chromatography of LmrR variants (y-axis is A_{210} and x-axis is retention time in minutes)

WT-LmrR

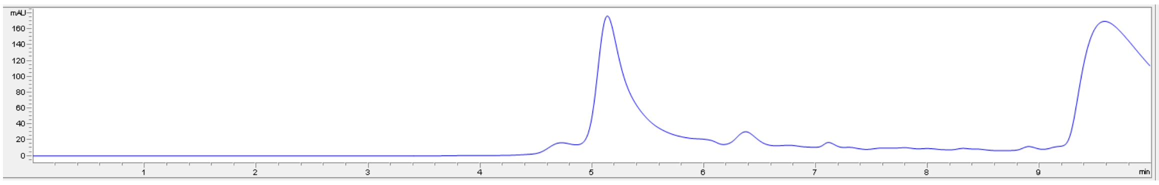

Val15-1

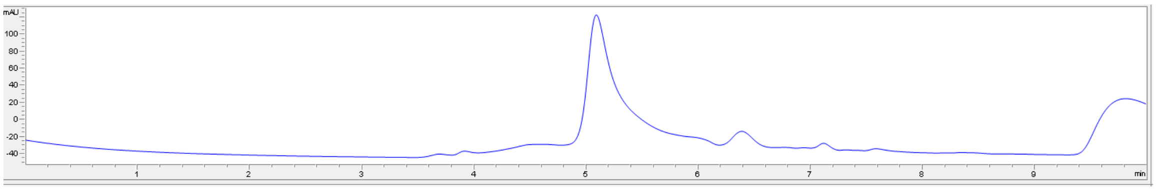

Val15-2

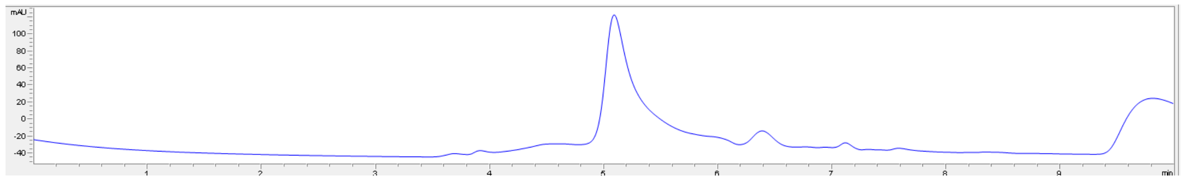

Val15-3

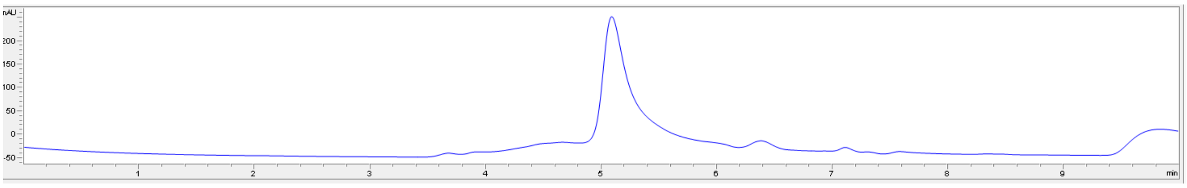

Asp19-1

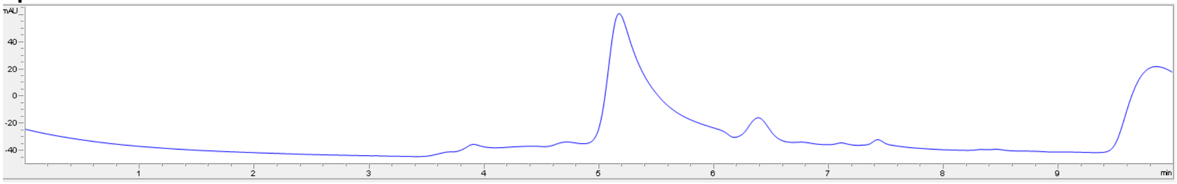

Asp19-2

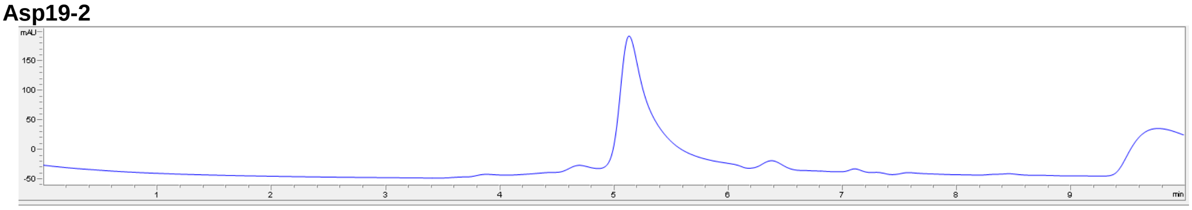

Asp19-3

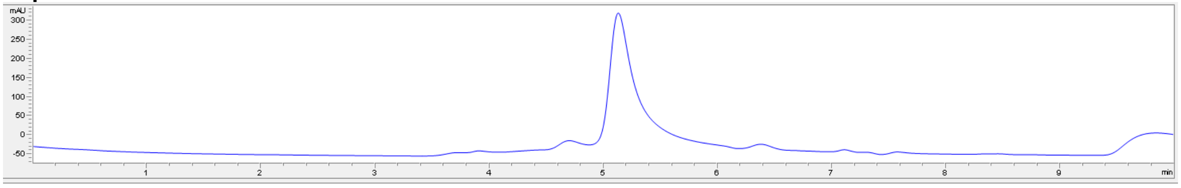

Met89-1

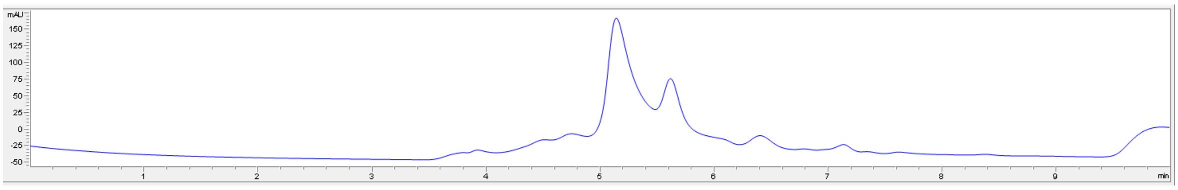

Met89-2 (found to be unstable and prone to precipitation)

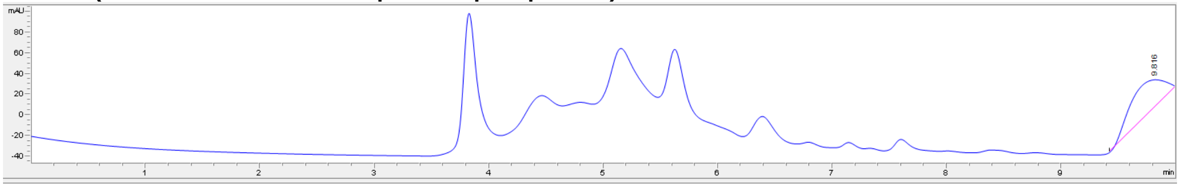

Met89 3

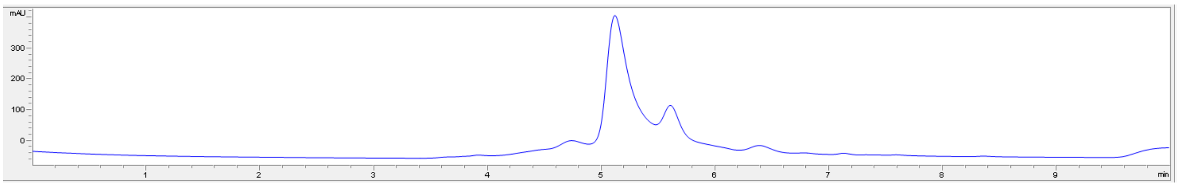

Phe93-**1**

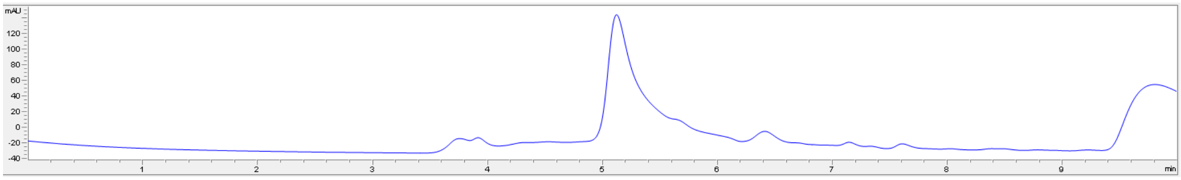

Phe93-**2**

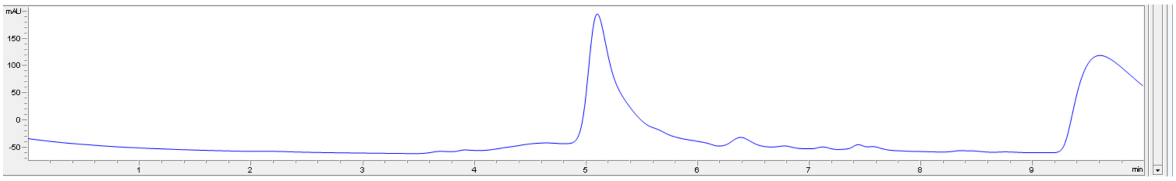

Phe93-**3**

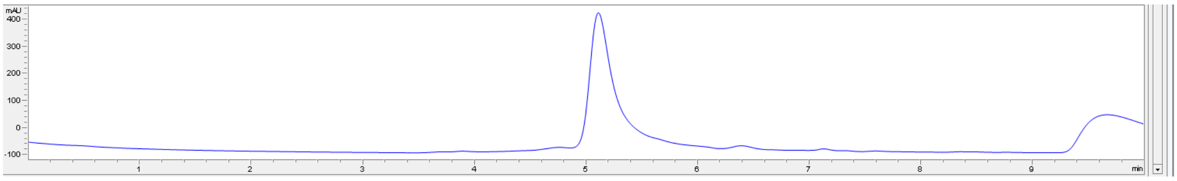

6. Catalytic activity screening reactions

Activity screening for the LmrR variants (>95% purity by SDS-PAGE analysis) were performed in total reaction volumes of 100 µL. To 50 µL (100 µg, 6.8 nmol) of protein solution in the reaction buffer (50 mM NaP_i, 150 mM NaCl, pH 7.0) in a microcentrifuge tube was added 5.0 µL of cinnamaldehyde **4a** (8.9 µg, 68 nmol, 1 equiv., stored in methanol), followed by 5 µL of BNAH (28 µg, 136 nmol, 2 equiv., stored in methanol). All reactions were adjusted to reach 100 µL volume containing 10% methanol.

Activity screening for the DHFR variants (>95% purity by SDS-PAGE analysis) were performed in total reaction volume of 100 µL. The concentration of NADPH was determined by measuring the UV absorbance at 340 nm using an extinction coefficient of 6200 M^{-1} cm^{-1}.[9] To 50 µL (100 µg, 5.12 nmol) of protein solution in the reaction buffer (50 mM NaP_i, 150 mM NaCl, pH 7.0) in a microcentrifuge tube was added 5.0 µL solution of cinnamaldehyde **4a** (8.9 µg, 52 nmol, 1 equiv., stored in methanol), followed by 5.0 µL solution of NADPH (77 µg, 104 nmol, 2 equiv., stored in reaction buffer) or BNAH (28 µg, 136 nmol, 2 equiv., stored in methanol). All reactions were adjusted to reach 100 µL volume containing 5% methanol.

The control experiments for pyrrolidine, UAA **1** and MacMillan organocatalyst were performed in total reaction volume of 150 µL. To a microcentrifuge tube, was added 50 µL of a stock solution of cinnamaldehyde (512 nmol, 1 equiv., stock in methanol), followed by 50 µL of an aqueous stock solution of NADPH (2.56 µmol, 5 equiv.) and 50 µL of the stock solution of catalyst (0.5 equiv., stock in water (for **1** and MacMillan catalyst) and methanol (for pyrrolidine)).

The reactions were placed in a thermomixer at 25 °C and 500 rpm for 18 hours. They were halted by adding 200 µL of DCM, vortexed vigorously and centrifuged to separate the layers (20,000 rcf, 3 min, rt); 100 µL of the organic layer was removed and subjected to GC-MS analysis following previously described procedure (see Pg S28 for setup).[10] In addition to the protein variants, catalyst free controls and reactions with the wild-type protein were also performed. Unnatural amino acid **1** was also used with the same loading as the protein catalyst. Each reaction was performed in triplicate (**Tables S2**, **S3** & **S4**).

Table S2. Conversion of cinnamaldehyde **4a** to the product dihydro-cinnamaldehyde **5a** by **LmrR** variants using BNAH as the hydride donor after 18 h of incubation. Conversion was estimated by comparing the peak area of **5a** against the standards measured by GC-MS (see Pg S28 for setup and Pg S10 for reaction conditions).

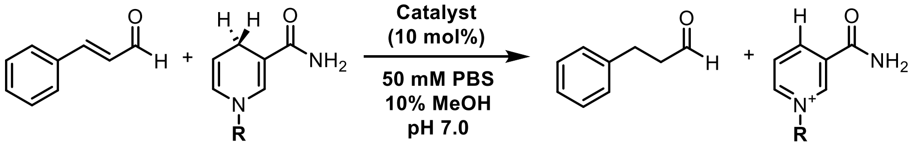

4a **BNAH** **5a** **BNA⁺**

Catalyst	Conversion 1 (%)	Conversion 2 (%)	Conversion 3 (%)	Mean (%) ± standard derivation
**Val15-1**	31	14	17	21 ± 9
**Val15-2**	15	11	11	12 ± 3
**Val15-3**	7	8	8	7 ± 1
**Asp19-1**	18	17	15	17 ± 2
**Asp19-2**	4	3	3	4 ± 1
**Asp19-3**	33	33	28	31 ± 3
**Met89-1**	12	8	1	7 ± 6
**Met89-2**	5	4	5	5 ± 1
**Met89-3**	9	11	9	10 ± 1
**Phe93-1**	68	57	49	58 ± 9
**Phe93-1[a]**	4	3	3	4 ± 0
**Phe93-2**	3	2	2	2 ± 0
**Phe93-3**	13	10	13	12 ± 2
**Wt**	2	2	2	2 ± 0
**UAA 1**	20	21	18	20 ± 2
**(-) control**	0	0	0	0

a) BNAH was replaced with the same amount of NADPH.

Table S3. Conversion of cinnamaldehyde **4a** to the product dihydro-cinnamaldehyde **5a** by **DHFR** variants using NADPH as the hydride donor after 18 h of incubation. Conversion was estimated by comparing the peak area of **5a** against the standards measured by GC-MS (see Pg S28 for setup and Pg S10 for reaction conditions).

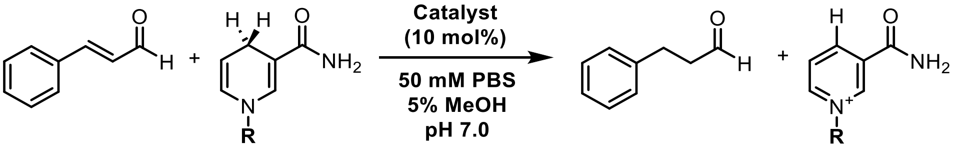

4a **NADPH** **5a** **NADP⁺**

Catalyst	Conversion 1 (%)	Conversion 2 (%)	Conversion 3 (%)	Mean (%) ± standard derivation
**Ala7-1**	89	88	99	92 ± 6
**Phe31-1**	18	18	18	18 ± 0
**Ser49-1**	0	0	0	0
**Wt**	0	0	0	0
**UAA 1**	0	0	0	0
**(-) control**	0	0	0	0

Table S4. Conversion of cinnamaldehyde to the product hydro-cinnamaldehyde **5a** by **DHFR** variants using BNAH as the hydride donor after 18 h of incubation. Conversion was estimated by comparing the peak area of **5a** against the standards measured by GC-MS (see Pg S28 for setup and Pg S10 for reaction conditions).

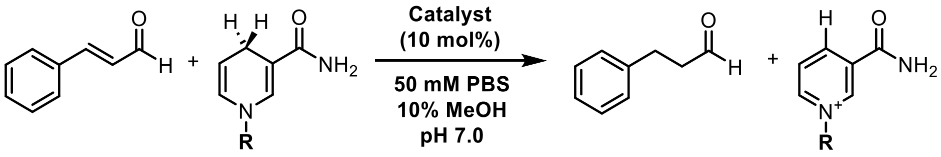

4a **BNAH** **5a** **BNA$^+$**

Catalyst	Conversion 1 (%)	Conversion 2 (%)	Mean (%) ± standard derivation
**Ala7-1**	31	37	34 ± 4
**Phe31-1**	15	23	19 ± 4
**Ser49-1**	0	0	0
**(-) control**	0	0	0

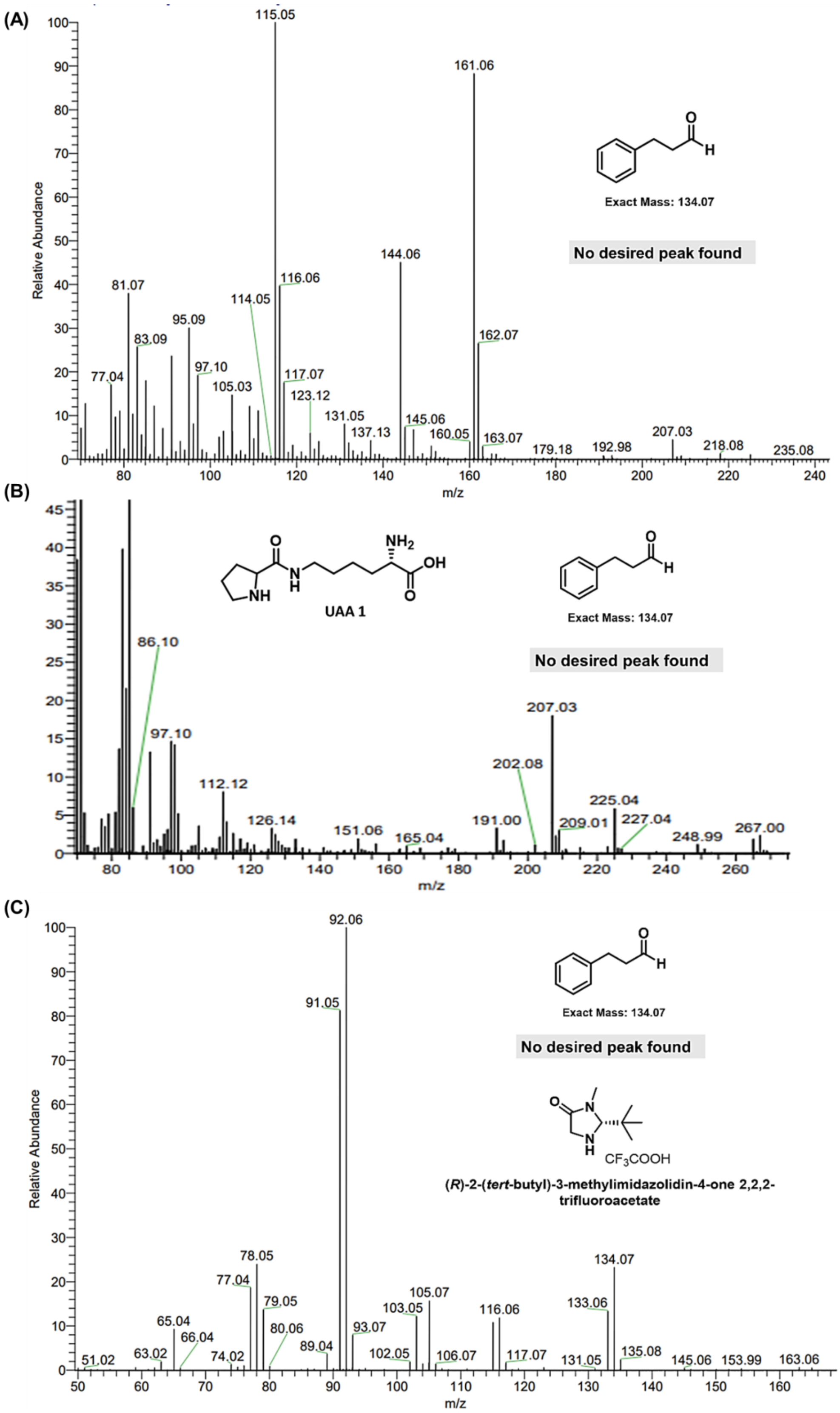

Figure S5. Control experiments for testing the formation of **5a** using secondary amine organocatalysts (**A**) pyrrolidine, (**B**) UAA 1 and (**C**) First-generation MacMillan catalyst and NADPH as hydride donor. No product formation was observed in any of these reactions.

7. Iminium ion intermediate characterization

The iminium ion intermediate was trapped by reduction to a tertiary amine and subsequently analyzed by mass spectrometry. To a microcentrifuge tube, **LmrR-Phe93-1** or **DHFR-Ala7-1** (100 µg, 0.51 nmol and 0.67 nmol, respectively) in PBS buffer (50 mM NaP_i, 150 mM NaCl, pH 7.0) was added and mixed with 20 equiv. of cinnamaldehyde (stock in methanol). All reactions were adjusted to reach 100 µL volume of PBS buffer containing 10% methanol. The reactions were placed in a thermoshaker for 2 hours at 25 °C with agitation at 500 rpm. Subsequently, 50 equiv. of $NaCNBH_3$ (in methanol) was added to the reactions which were left overnight under the above reaction conditions. The protein samples were subjected to micro-centrifuge ultrafiltration (10 kDa cut-off columns, Millipore), removing the small molecules and exchanging the samples into Tris buffer (50 mM, pH 8.0). 10.0 µL of the sample was removed and analyzed by protein mass spectrometry. The remaining samples were then digested with 1:20 (chymotrypsin:protein substrate) overnight at 25 °C. The digested samples were analyzed by LC-MS analysis (**Fig. S6-S8**).

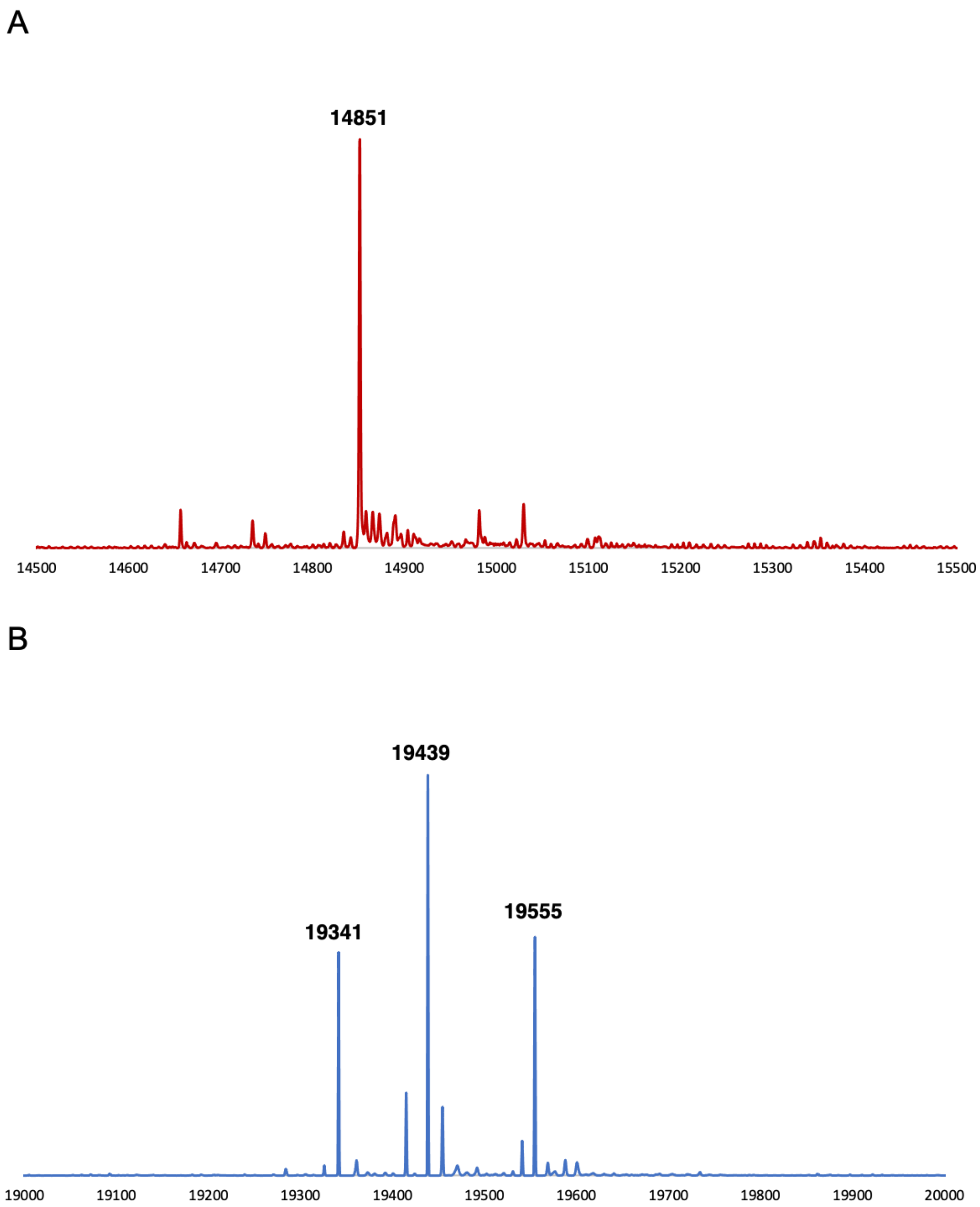

Figure S6. Deconvoluted ESI mass spectra for protein variants treated with cinnamaldehyde and sodium cyanoborohydride. (**A**) **LmrR-Phe93-1** cinnamaldehyde adduct mass was calculated to be 14851 Da and observed to be 14851 Da. (**B**) **DHFR-Ala7-1** cinnamaldehyde adduct mass was calculated to be 19555 Da and observed to be 19555 Da. Mass of 19439 Da equates to the unreacted parent **DHFR-Ala7-1**. Mass of 19341 Da equates to the removal of the prolyl-group of unnatural amino acid **1**, a mass not observed in the protein prior to the sodium cyanoborohydride reaction.

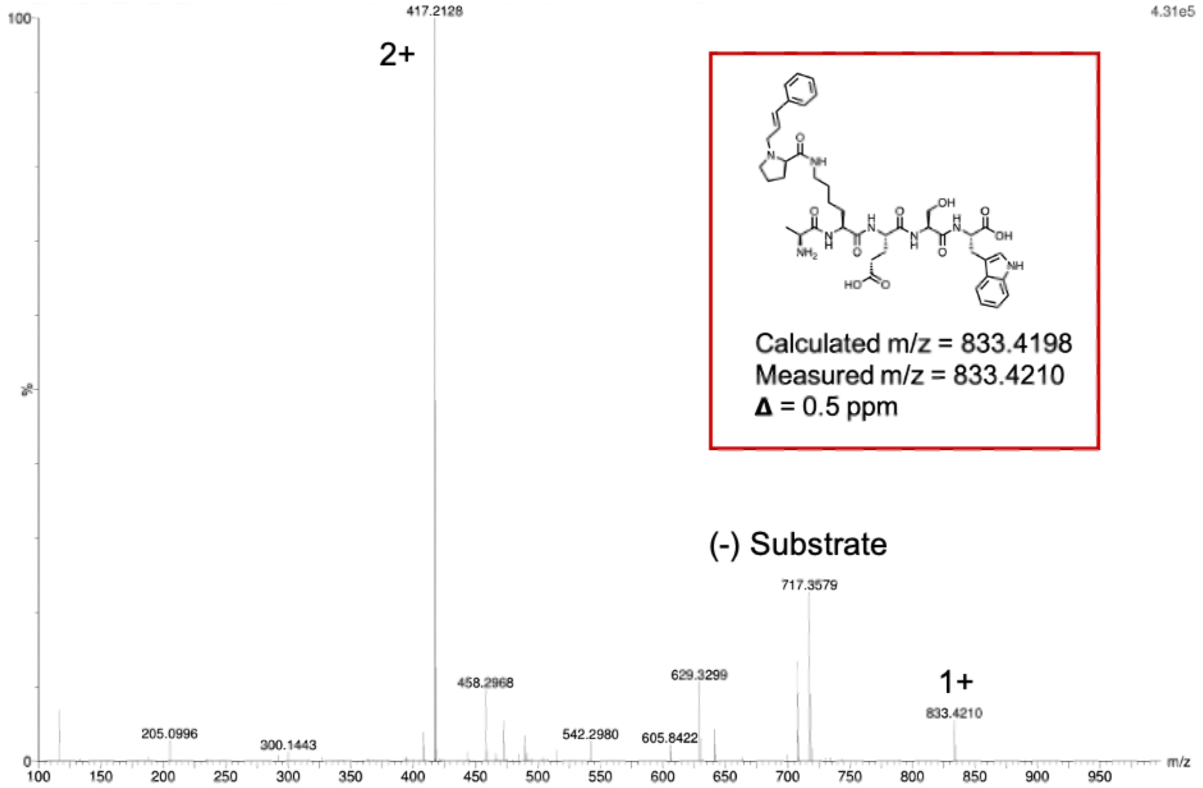

Figure S7. High-resolution mass analysis of the peptide fragment containing UAA **1** covalently linked to the substrate cinnamaldehyde **4a** produced from the chymotrypsin digest of **LmrR-Phe93-1.**

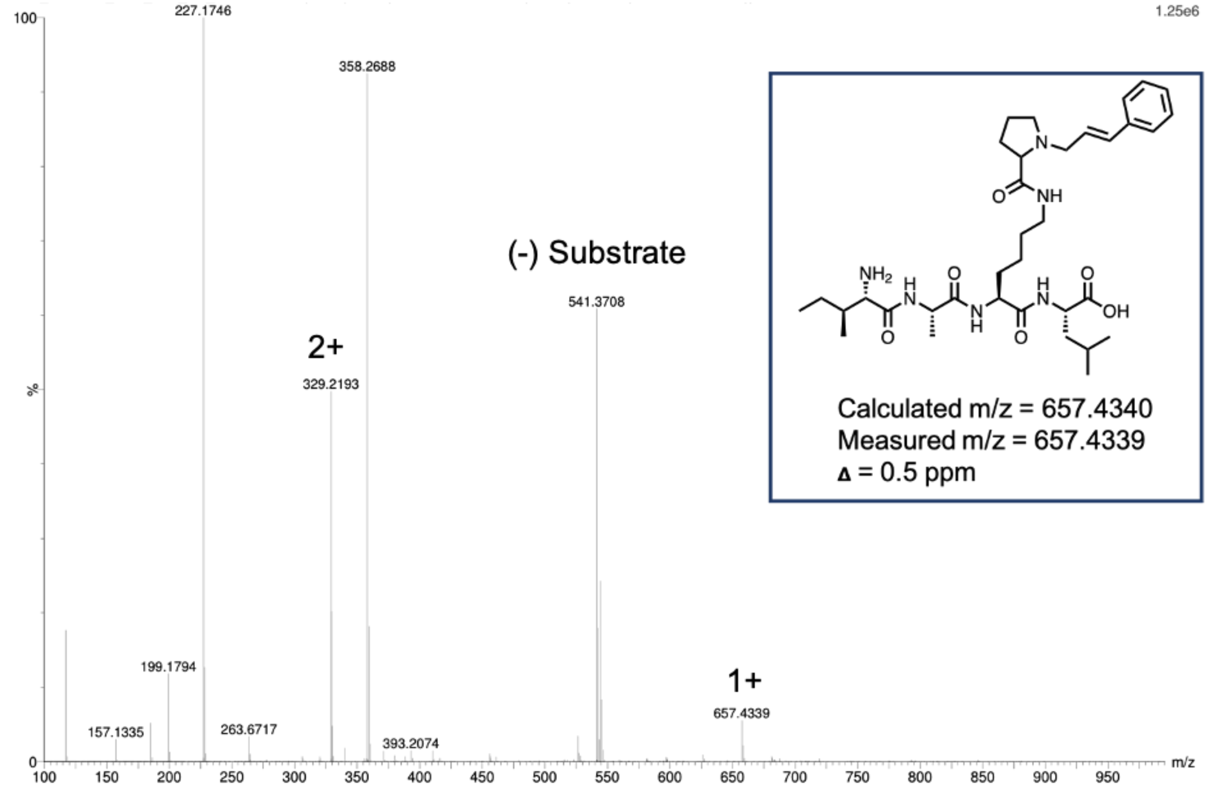

Figure S8. High-resolution mass analysis of the peptide fragment containing UAA **1** covalently linked to the substrate cinnamaldehyde **4a** produced from the chymotrypsin digest of **DHFR-Ala7-1.**

8. Kinetic evaluations

To assess the linear phase of the transfer hydrogenation reaction, its formation over time was evaluated by GC-FID following a procedure similar to previously described (see also Pg S28).[11] The saturating concentrations of the substrate for catalysis were first assessed. The reaction vessel contained 50 µM of the protein catalyst and various concentrations of cinnamaldehyde **4a** (prepared as stock in methanol), and the reactions were initiated by adding 5 mM of the hydride donor and placed in a thermomixer at 25 °C and 500 rpm. All the reactions were adjusted to contain 10% and 5% methanol for LmrR and DHFR reactions, respectively. The reactions were halted by adding 200 µL of DCM, vortexed vigorously, and centrifuged to separate the layers (20,000 rcf, 3 min, rt). 100 µL of the organic layer was removed and analyzed by GC-FID. Conversely, to determine the saturating concentration of the hydride donor, 1 mM cinnamaldehyde and various concentrations of the hydride source were used. We observed that the turnover rate constants remained largely unchanged when cinnamaldehyde concentration is ≥1 mM but it is prone to precipitation. Reactions were performed in triplicate. For the **LmrR-Phe93-1** reactions at least 3 mM of BNAH was needed to reach kinetic saturation, whereas for the **DHFR-Ala7-1** reactions only 150 µM of NADPH was sufficient. Accordingly, values of the catalytic turnover constant (k_{cat}) were estimated in PBS buffer by using saturating concentrations of cinnamaldehyde and the respective donors (**Table S5** & **S6**).

For initial velocity approximation, two hours was determined to be the end of the linear phases (**Fig. S9**) and so further kinetic characterizations were performed at this time point. BNAH of different concentrations (prepared as a stock in methanol) was added to centrifuge tubes containing 50 µM of the **LmrR-Phe93-1** and 1 mM of cinnamaldehyde **4a** (prepared as stock in methanol). They were placed in a thermomixer at 25 °C and 500 rpm for 2 hours. All reactions were adjusted to contain 10% of methanol. The reactions were halted by adding 200 µL of DCM, vortexed vigorously and then centrifuged to separate the layers (20,000 rcf, 3 min, rt); 100 µL of the organic layer was removed and analyzed by GC-FID. Reactions were performed in triplicate (**Table S5** & **S6**). At lower concentrations of BNAH (200-800 µM), attempts to sample the reactions at earlier time points (e.g., 15, 30, 60 mins) were made. However, conversion was too low to accurately determine the product concentration, and hence the K_M reported in **Figure 3** and **Table S5** was an approximation. Analysis was performed using the software GraphPad Prism with the non-linear regression Michaelis-Menten output.

Similarly, 20 µM of the **DHFR-Ala7-1** catalyst and 1 mM of cinnamaldehyde **4a** (prepared as stock in methanol) were added with various concentrations of NADPH (prepared as a stock in reaction buffer) and were placed in a thermomixer at 25 °C and 500 rpm for 2 hours. All reactions were adjusted to 100 µL volume containing 5% methanol. They were halted as described above for GC-FID analysis. At lower NADPH concentrations (40-100 µM) attempts to sample the reactions at earlier time points were made, but conversion was too low to accurately determine product concentration. Attempts to address this issue by use of UV-Vis spectroscopy was made, but the reaction rate constants were too slow to be accurately determined. Hence, the stopped assay was used, and the K_M reported in **Figure 3** and **Table S6** was an approximation. At high concentration (> 1 mM), cinnamaldehyde is prone to precipitation and hydrate formation within an aqueous environment. Furthermore, unlike Sav, both the **LmrR-Phe93-1** and **DHFR-Ala7-1** reactions were measured in lower amount of organic solvent (10% vs 25% methanol). Accordingly, the K_M constants for cinnamaldehyde were not measured.

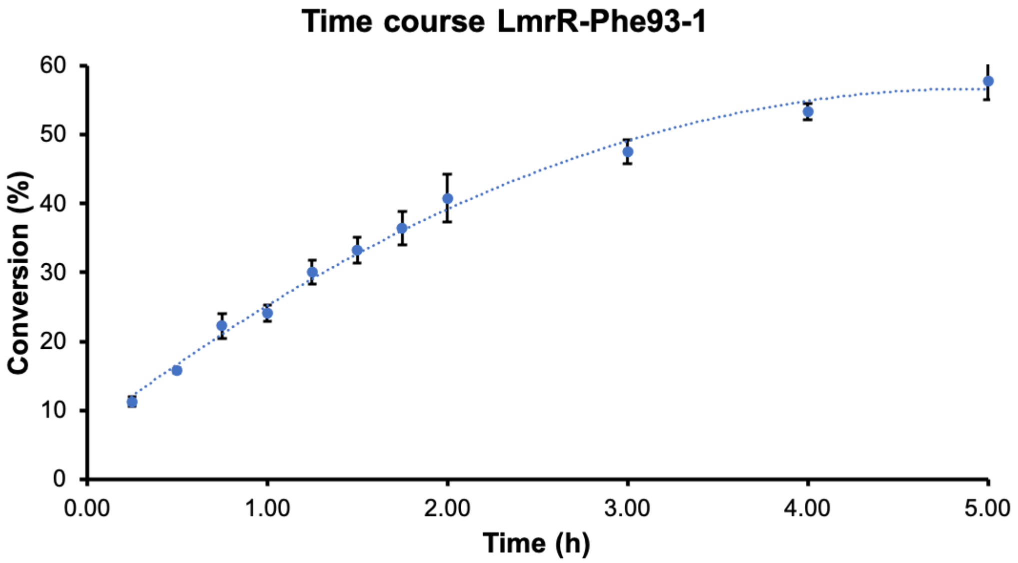

Figure S9. Time dependence for the conversion of cinnamaldehyde (1 mM) to its reduced product in the presence of BNAH (5 mM) and 50 µM of **LmrR-Phe93-1.**

Table S5. The estimated turnover (k_{cat}) and Michaelis (K_M) constants for **LmrR-Phe93-1**

[BNAH] (µM)	Mean Rate ± standard error ($x10^{-4}$ s^{-1})		
**200**	1.7	±	0.2
**400**	2.6	±	0.3
**600**	2.9	±	0.9
**800**	4.6	±	0.8
**1000**	5.5	±	0.4
**2000**	7.3	±	0.3
**3000**	10.0	±	0.9
**4000**	10.1	±	0.1
**5000**	9.2	±	1.0

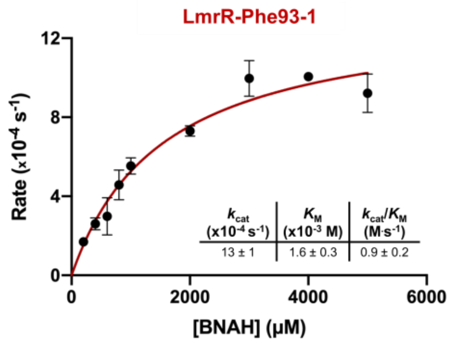

Table S6. The estimated turnover (k_{cat}) and Michaelis (K_M) constants for **DHFR-Ala7-1**

[NADPH] (µM)	Mean Rate ± standard error ($x10^{-4}$ s^{-1})		
**40**	0.8	±	0.4
**50**	1.7	±	0.2
**60**	1.9	±	0.5
**75**	2.3	±	0.2
**100**	3.2	±	0.3
**125**	3.5	±	0.6
**150**	3.7	±	0.4
**250**	4.6	±	0.3
**300**	4.4	±	0.4
**500**	3.8	±	0.1

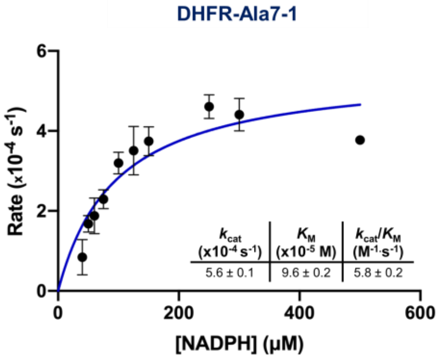

9. NADPH hydride transfer selectivity characterization

NADPD concentration was determined by measuring the UV absorbance at 340 nm using the extinction coefficient of 6200 M^{-1} cm^{-1}.[9] To 50 µL (100 µg, 6.8 nmol) of **DHFR-Ala7-1** in the reaction buffer (50 mM NaP_i, 150 mM NaCl, pH 7.0) in a microcentrifuge tube was added a 5.0 µL solution of cinnamaldehyde **4a** (8.9 µg, 68 nmol, 1 equiv., stock in methanol), followed by 5.0 µL solution of NADPD (28 µg, 136 nmol, 2 equiv., stock in the reaction buffer), resulting in a total volume

of 60 µL. The reactions were placed in a thermomixer at 25 °C and 500 rpm for 18 hours. The reactions were halted by adding 200 µL of DCM, vortexed vigorously and then centrifuged to separate the layers (20,000 rcf, 3 min, rt). 100 µL of the organic layer was removed and subjected to analysis by GC-MS following a procedure similar to previously described.[10] Control reactions with NADPH were performed alongside. Each reaction was performed in triplicate (**Fig. S10**).

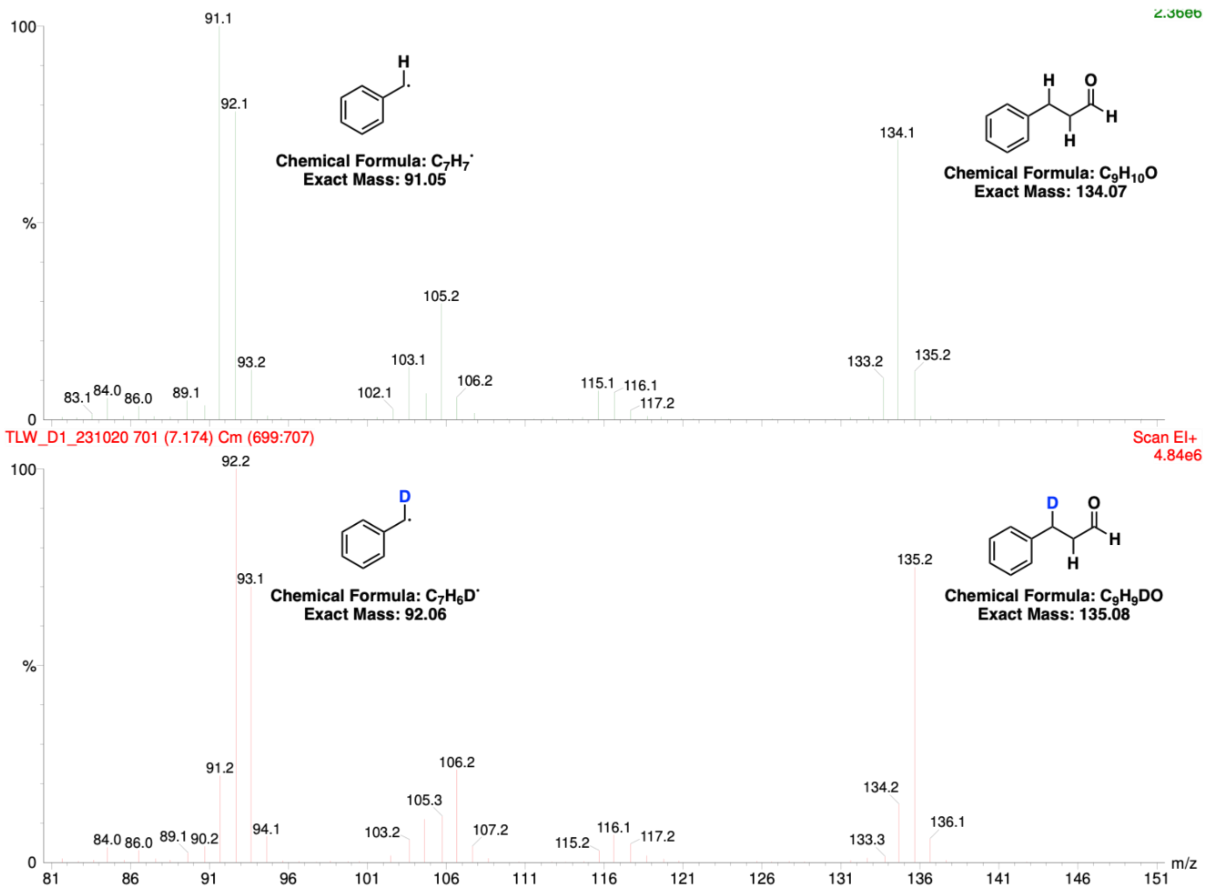

Figure S10. ESI-MS analysis of the product generated from the reactions between cinnamaldehyde **4a** and NADPH (top) and NADPD (bottom) catalyzed by **DHFR-Ala7-1**.

10. Kinetic isotope effect study

The oxidation of NADPH and NADPD under saturating conditions was measured comparatively using a UV plate reader assay. To 90 µL of the protein catalyst solution (final concentration 25 µM in 50 mM NaP_i, 150 mM NaCl at pH 7.0) on a 96 well plate, 5.0 µL of NADPH or NADPD dissolved in the same buffer (final concentration 250 µM) was added. The samples were placed in the plate reader (BMG Labtech FLUOstar OPTIMA microplate reader) at a fixed temperature of 25 °C for five minutes. After the allotted time the plate was ejected and 5.0 µL of cinnamaldehyde (final concentration 1 mM, stock in methanol) was added to each reaction. The oxidation of NADPH/D was monitored at 340 nm for two hours. The reactions were performed in triplicate. Measurements were taken every minute over 2 hours (**Fig. S11**). Following completion of the reaction the slope was determined for both NADPH and NADPD. The kinetic isotope effect, if any, was determined using the following equation:

$$KIE = \frac{k^{NADPH}}{k^{NADPD}}$$

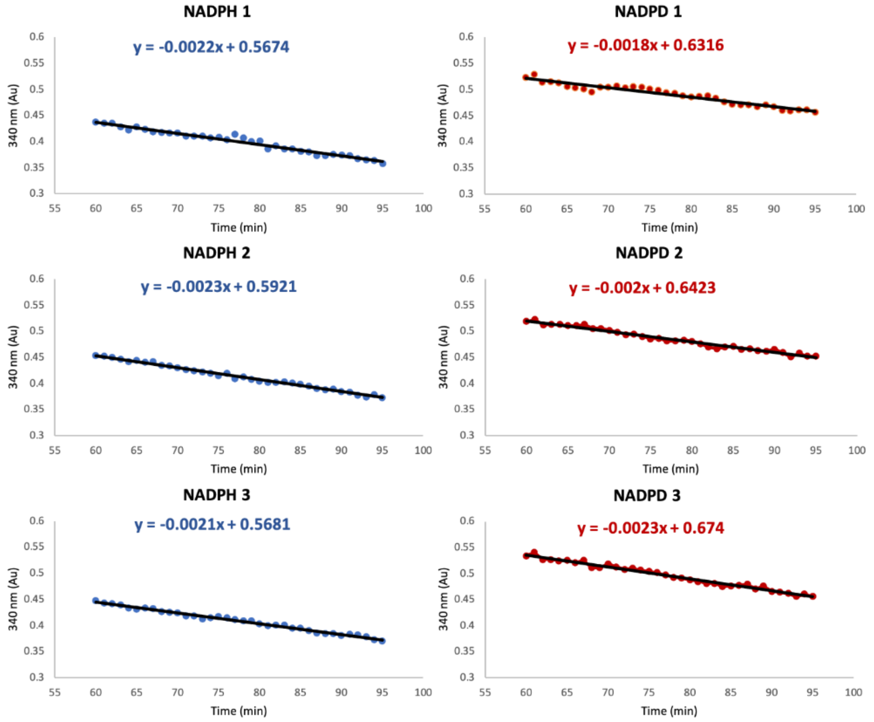

	Exp 1.	Exp. 2	Exp. 3	Average	Std. Deviation
**NADPH (Slope)**	0.0022	0.0023	0.0021	0.0022	0.0001
**NADPD (Slope)**	0.0018	0.002	0.0023	0.0020	0.0003
**KIE**	1.22	1.15	0.92	1.10	0.16

Figure S11. Kinetic isotope effect (KIE) determined by UV-Vis spectroscopic assay.

11. Substrate scope

Conversion of the substates was estimated by a ^{1}H NMR spectroscopic assay following the change in the peaks corresponded to the aldehyde proton as previously described.[12, 13] A Bruker Advance 500 MHz NMR system equipped with a He cooled cryoprobe was used and each reaction was characterized with 256 scans. Each reaction was performed in triplicate and the mean yield (± standard deviation) reported. The yield was determined by ^{1}H NMR using nitrobenzene as an internal standard and was added after the completion of reaction.

To a microcentrifuge tube, 500 µL of **DHFR-Ala7-1** (5.1 nmol) in PBS buffer (50 mM NaP_i, 150 mM NaCl, pH 7.0), 400 µL PBS were added followed by 50 µL of a stock solution of the α,β-unsaturated carbonyl substrate (**4a-4i**, 512 nmol, 1 equiv., stock in methanol) and 50 µL of a stock solution of NADPH (3.4 µmol, 5 equiv., stock in the PBS buffer). The reaction was adjusted to 1000 µL PBS buffer containing 5% methanol. The reactions were placed in a thermoshaker and shook at 500 rpm for 48 hours at 25 °C. Following the completion of the reactions, DCM was added (500 µL) and the sample vortexed vigorously. The phases were separated by centrifugation (20,000 rcf, 3 min, rt). The organic phase was removed and placed in a new microcentrifuge tube. This was repeated and the two extractions combined. The solvent was removed under nitrogen and the remaining residue re-dissolved in $CDCl_3$ (600 µL). Each sample was then subjected to ^{1}H NMR and the conversion determined by integration of the aldehyde and internal standard protons (Table S7).[14, 15] The conversion of starting material was performed in triplicate and calculated using the below mentioned formula:

$$\text{Conver}sion\ (\%) = 100 - \left(\frac{N_{rs}}{N_{SM}}\ x\ 100\right)$$

and, N_{rs} is calculated using the formula:

$$\frac{N_{rs}}{N_{is}} = \frac{\left(\frac{I_{RS}}{N_{RS}}\right)}{\left(\frac{I_{IS}}{N_{IS}}\right)}$$

where I_{RS} and N_{RS} denote the integration value and number of corresponding protons respectively of the residual starting material, and I_{IS} and N_{IS} denote the integration value and number of corresponding protons respectively of the internal standard and N_{SM} denotes the initial moles of substrate. For the nitro analogue **4g**, the presence of various doublets around 8.1 ppm in the ^{1}H NMR spectrum (~50% conversion) suggested the formation of multiple unidentified side-products. In the case of the ketone analogue **4h**, although a peak corresponding to the reduced product was detected in ESI-MS, it was not observed in ^{1}H NMR spectroscopy. Regarding the prochiral analogue **4i**, around 20% of an unidentified side product was indicated by a distinct doublet at 10.1 ppm in the ^{1}H NMR spectrum (Figure S18). This could be attributed to the formation of methyl stabilized benzylic carbocation, susceptible to either attack by various nucleophiles in the reaction medium or deprotonation of the methyl group which resulted in rearrangement of cation. Nevertheless, the use of **4i** facilitated the enantioselectivity assay that follows.

Table S7. Substrate scope analysis of **DHFR-Ala7-1.** See Pg S21 for reaction conditions.

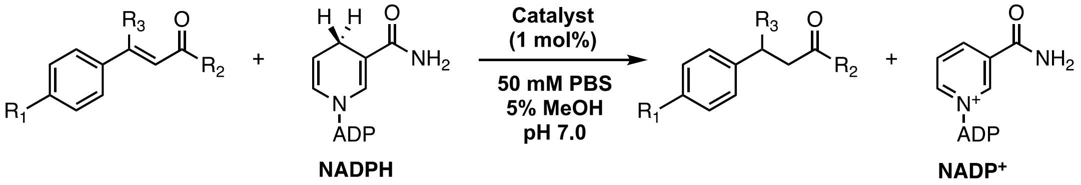

**Substrate**		**Substrate conversion (%) (R_1, R_2, R_3)**			**Mean substrate conversion (%) ± standard derivation**
**4a**	R_1 = H, R_2 = H, R_3 = H	86	96	93	92 ± 5
**4b**	R_1 = Cl, R_2 = H, R_3 = H	50	60	58	56 ± 5
**4c**	R_1 = F, R_2 = H, R_3 = H	48	60	50	53 ± 6
**4d**	R_1 = Br, R_2 = H, R_3 = H	43	49	58	50 ± 7
**4e**	R_1 = OMe, R_2 = H, R_3 = H	45	52	55	50 ± 5
**4f**	R_1 = Me, R_2 = H, R_3 = H	63	65	74	67 ± 5
**4g**	R_1 = NO_2, R_2 = H, R_3 = H	91	95	97	94[a] ± 3
**4h**	R_1 = Cl, R_2 = CH_3, R_3 = H	-			Detected by GC-MS
**4i**	R_1 = H, R_2 = H, R_3 = CH_3	>99,	>99	>99	>99[b] ± 0

[a] Various unidentified byproducts were observed as indicated by ^{1}H NMR spectroscopy accounting for ~50% of substrate conversion (see Section 17);

[b] A byproduct was observed accounting for ~20% of substrate conversion as revealed by ^{1}H NMR spectroscopy (see also Section 17).

12. Enantioselectivity Assay

The protein catalyst **DHFR-Ala7-1** and its mutants, Asp27Ala, Asp27Asn, Tyr100Phe and Tyr100Glu, (2.0 mg/mL) was prepared in phosphate buffer (50 mM NaP_i, 150 mM NaCl, pH 7.0). For reaction test in different pHs, they were exchanged into formate (50 mM formic acid, 150 mM NaCl; for pH range 4.0-5.0), phosphate buffer (50 mM NaP_i, 150 mM NaCl, pH 7.0; for pH range 6.0-8.0) or carbonate-bicarbonate buffer (50 mM $NaHCO_3$; for pH range 9.0-11.0). Further adjustments were made by adding solutions of 0.01 M NaOH or 0.01 M HCl dropwise.

a) **Reference.** For generating the racemic product equal quantities of D- and L-proline were used as catalysts, whereas for assigning the respective enantiomers ***S*-** and ***R*-5i** (*R*)-2-tert-butyl-3-methylimidazolidin-4-one was recruited.[16] For the respective reactions, equal quantities of D- and L- proline (0.6 mg each, 10.9 µmol in total, 0.2 equiv.) or (*R*)-2-tert-butyl-3-methylimidazolidin-4-one (3 mg, 10.9 µmol, 0.2 equiv.) were weighed into a GC vial. After addition of water-saturated $CDCl_3$ (345 µL), (*E*)-3-phenylbut-2-enal (**4i**, 8.2 µL, 54.7 µmol, 1 equiv.) and n-decane (5.33 µL, (27.4 µmol, 0.5 equiv.) were added. A small sample (10 µL) was diluted in hexane (1 mL) to observe the initial ratio of substrate and n-decane. Hantzsch ester (20.8 mg, 82.1 µmol, 1.5 equiv.) was added. The reaction progress was monitored by GC, a small sample (10 µL) was diluted in hexane (1 mL) and analyzed by chiral GC.

b) **DHFR-Ala7-1 and variants analysis**. In a PCR tube, 10 µL of (*E*)-3-phenyl-2-butenal (**4i**) stock solution (416 nmol, 1 equiv., stock solution in methanol) and 10 µL of internal standard n-decane (208 nmol, 0.5 equiv) were added. 10 µL of this mixture was taken and diluted to 100 µL using hexane for GC-MS analysis, assessing the initial ratios of the starting material to internal standard. To conduct the protein catalyst reaction, the respective protein (DHFR-Ala7-1 and its variants) was added in a PCR tube, followed by **4i** (416 nmol, 1 equiv., stock solution in methanol), internal standard n-decane (208 nmol, 0.5 equiv) and 20 µL of NADPH (616 µg, 838 nmol, 2 eq in the corresponding reaction buffer). The reaction was made up to 150 µL using respective reaction buffer and placed into a mixer at 4 °C, 1000 rpm and incubated for 24 hours. The reactions were halted by adding 200 µL of DCM and vortexed vigorously. The layers were separated through centrifugation (13,000 rpm, RT, 2 min) and 150 µL of the bottom organic layer was removed and subject to chiral and achiral GC analysis. Reaction conversion was determined in duplicates using the relative response factor between substrate **4i** and internal standard n-decane. The ratio of *S* and *R*-**5i** was assessed by chiral GC analysis, following previously established protocol based on the development of MacMillian catalyst.[16]

GC-MS was performed on a Clarus 680 (Perkin Elmer) fitted with an Elite-1 (Perkin Elmer) column (30 m × 0.25 mm internal diameter) and Clarus SQ 8 C mass spectrometer (Perkin Elmer) using an injection port ODP S7 temperature of 100 °C, a split ratio of 19:1 (**Fig. S12**). The oven was held at an initial temperature of 80 °C for 2 min, followed by a ramp of 8 °C/min to a final temperature of 280 °C which was held for 3 min. Chiral GC was performed on an Agilent 7890A GC system, using a Bodman chiraldex β-DM column (30 x0.25mm)[16] using an injection port temperature of 200 °C. The oven was held at an initial temperature of 80 °C for 4 min, followed by a ramp of 2 °C/min to a final temperature of 130 °C which was held for 4 min. The carrier gas used was helium.

(A) Chiral GC analysis of reactions catalyzed by (*R*)-(−)-2-(*tert*-Butyl)-3-methyl-4-imidazolidinone (MacMillian catalyst) and racemic proline

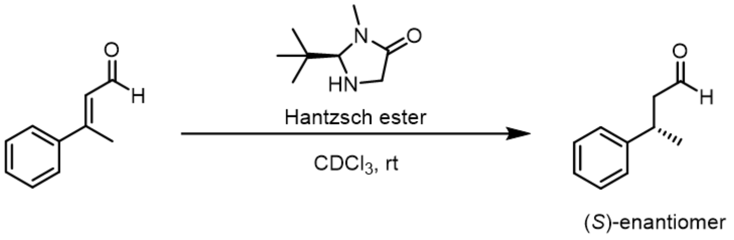

R- catalyst => S enantiomer

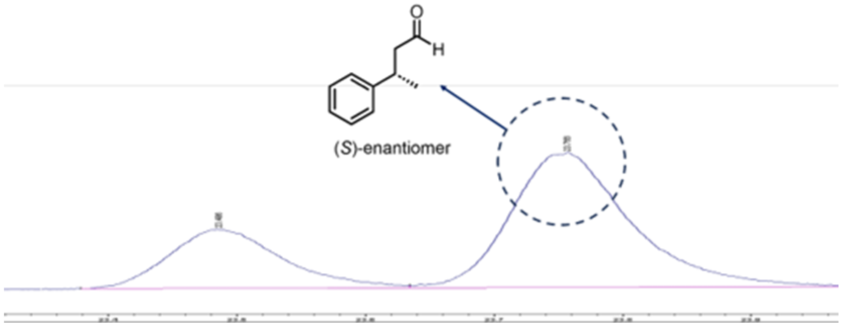

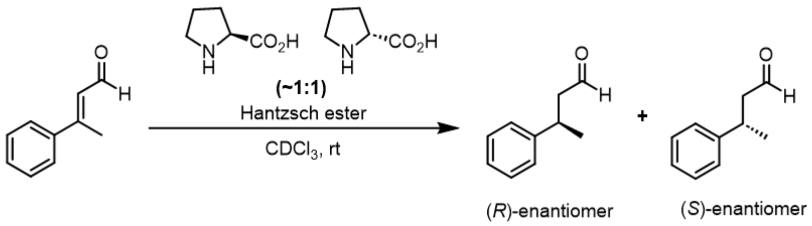

Racemic catalyst => *S* and *R* enantiomers

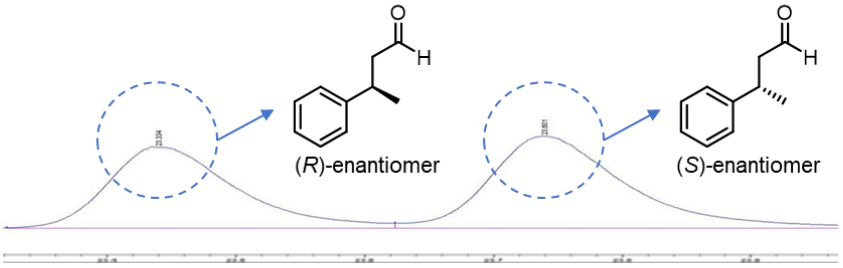

(B) DHFR-Ala7-1 at different pH's

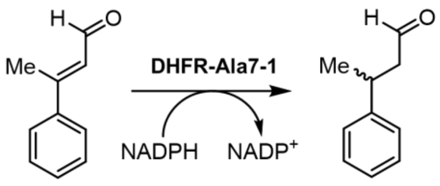

4i **S or R-5i**

Full-length trace of EcDHFR-Ala7-1 reaction at pH 7.0

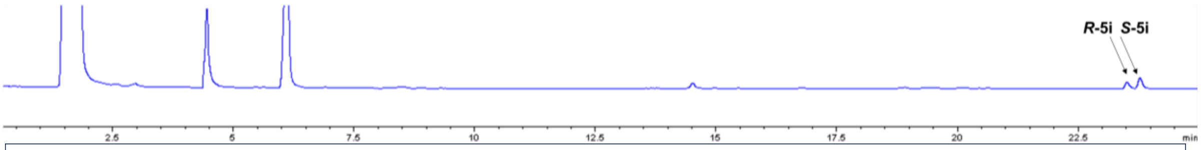

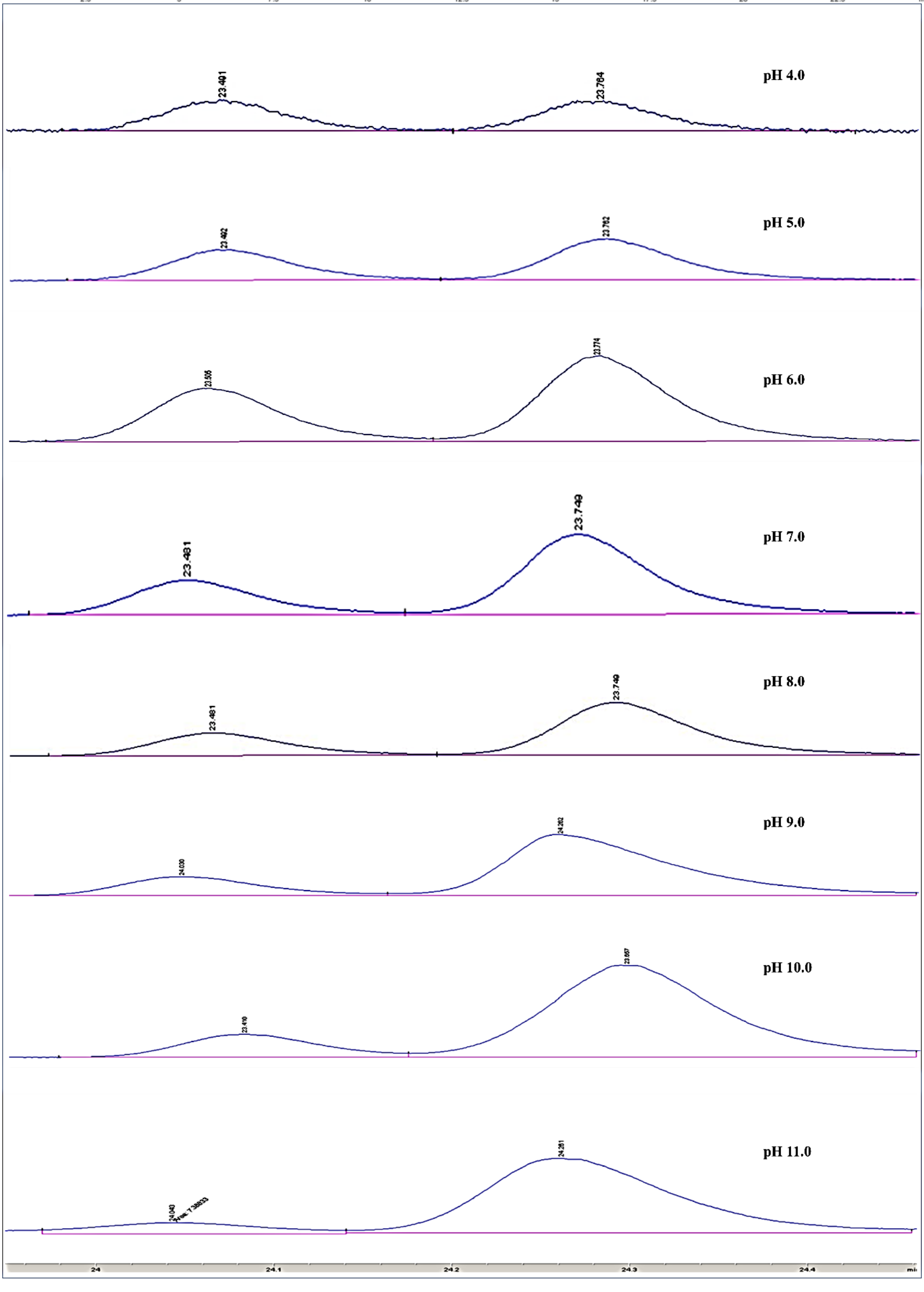

(C) (i) DHFR-Ala7-1 variants including D27N, D27A, Y100F and Y100E (pH 7.0)

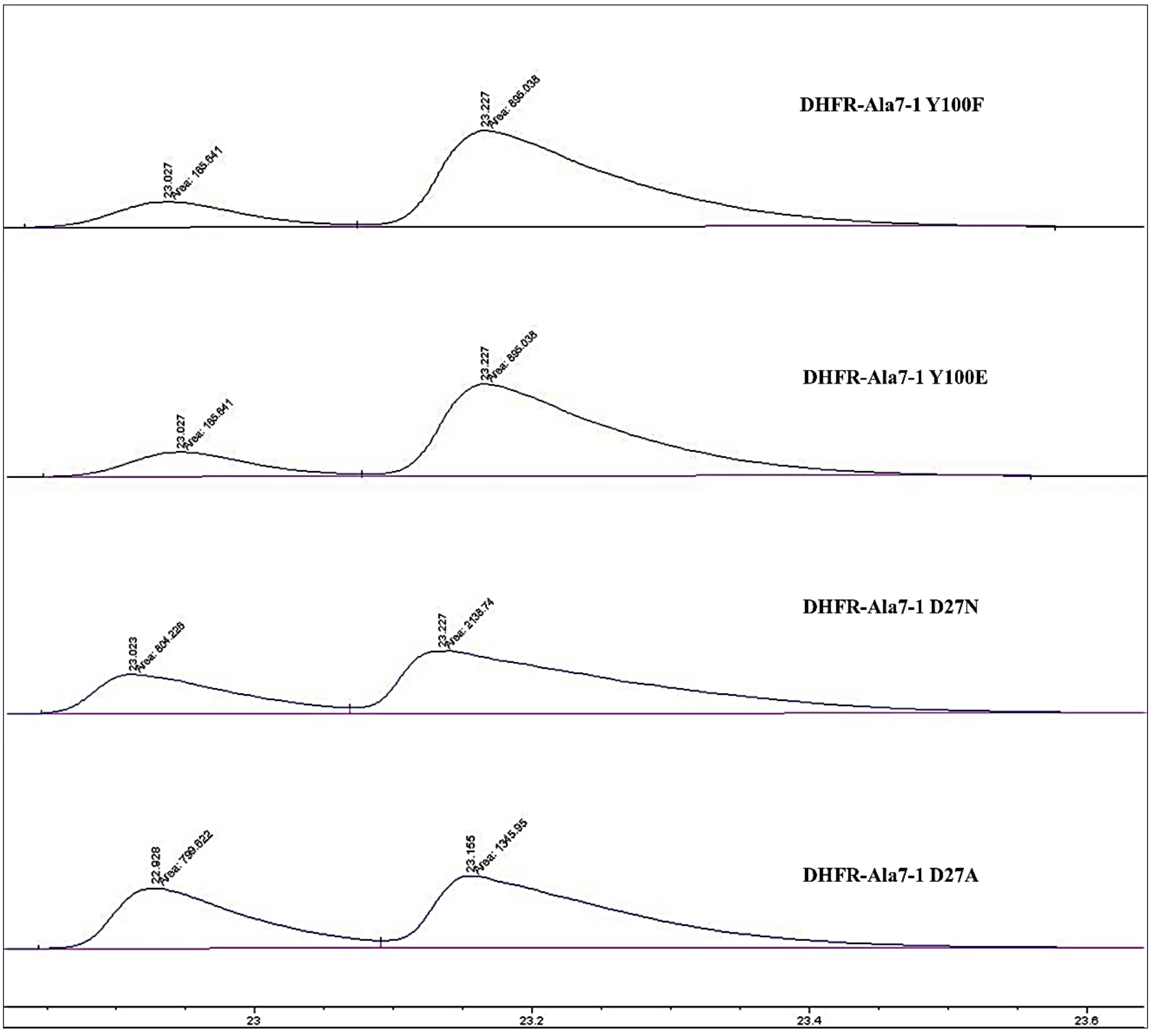

(ii) DHFR-Ala7-1 variants including D27N, D27A, Y100F and Y100E (pH 11.0)

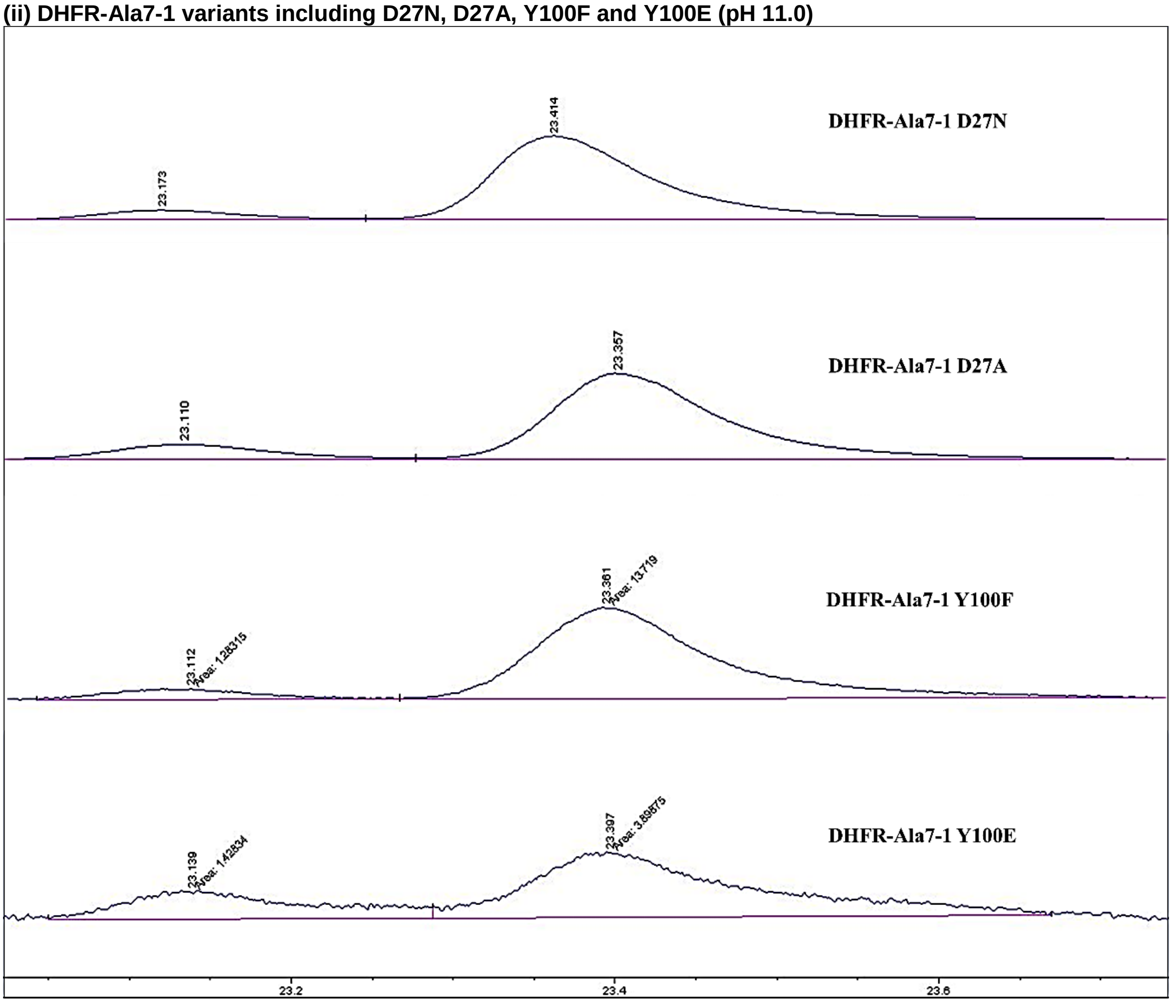

Figure S12. Chiral GC analysis for the formation of **5i** catalyzed by (**A**) MacMillian catalyst (*R*)-(−)-2-(*tert*-Butyl)-3-methyl-4-imidazolidinone and racemic proline, and (**B, C**) **DHFR-Ala7-1** and its variants under different pH's. Assignment was made based on previously reported protocol.[16]

13. Cofactor recycling assay

To a microcentrifuge tube was added 100 µM of the protein catalyst in reaction buffer (50 mM NaP_i, 150 mM NaCl, pH 7.0), 50 nM of glucose-6-phosphate dehydrogenase, 2 mM of glucose-6-phosphate and 1 mM of cinnamaldehyde (stock in methanol). Subsequently, NADPH (in reaction buffer) was added in varying concentrations. The final reaction volume was adjusted to 100 µL reaction buffer containing 5% methanol. The reactions were placed in a thermomixer at 25 °C and 500 rpm for 18 hours. The reactions were halted by adding 200 µL of DCM, vortexed vigorously, centrifuged to separate the layers (20,000 rcf, 3 min, rt) and 100 µL of the organic layer was removed for GC-FID analysis following a procedure similar to previously described.[11] Substrate to product conversion and total turnover number for NADPH (moles of product formed divided by moles of NADPH added) were determined from an average of three repeats (**Table S8**).

Table S8. Conversion of cinnamaldehyde (**4a**) to the product dihydro-cinnamaldehyde (**5a**) with and without glucose-6-phosphate dehydrogenase as the recycling enzyme. The total turnover number (TTN) for the cofactor NADPH refers to mole of product formed over mole of NADPH used.

[NADPH] (µM)	Conversion to product (%)		TTN (NADPH)
	(+) G6PDH	(-) G6PDH	
0.01	10 ± 0	0	10460
0.1	19 ± 0	0	1880
1	63 ± 2	0	632
5	76 ± 6	1 ± 0	153
10	91 ± 1	1 ± 0	91
50	72 ± 3	4 ± 0	14
125	80 ± 9	11 ± 1	6
250	71 ± 3	22 ± 1	3

14. Analytical chemistry

Protein liquid chromatography-mass spectrometry

Protein liquid chromatography mass spectrometry was acquired on a Waters Acquity H-Class UPLC system coupled to a Waters Synapt G2-Si quadrupole time of flight mass spectrometer. The column used was a Waters Acquity UPLC Protein C4 BEH column (300 Å, 1.7 mm, 2.1 x 100 mm) held at 60 °C. The flow rate was 0.2 mL/min and the gradient employed is highlighted in the **Table S9** below. Mass spectrometry data was collected in positive electrospray ionization mode and the data analyzed using Waters MassLynx 4.1. Deconvoluted mass spectra were generated using the maximum entropy 1 (MaxEnt1) software.

Table S9. Protein liquid chromatography-mass spectrometry chromatography parameters

Time (min)	H_2O (0.1% CHOOH)	ACN (0.1% CHOOH)
0	95	5
3	95	5
50	35	65
52	3	97
54	3	97
56	95	5
60	95	5

Analytical size exclusion chromatography

Analytical size exclusion chromatography was performed on an Agilent infinity 1260 HPLC. The column used was an Agilent Bio SEC-3 (150 Å, 3 mm, 4.6 x 300 mm) held at 20 °C. The elution was isocratic using PBS buffer (50 mM NaP_i, 150 mM NaCl, pH 7.0) and the flow rate was 1.0 mL/min. Detection was performed at 210 nm.

Gas chromatography-mass spectrometry (GC-MS)

Similar to previously described procedure,[10] the GC-MS assay for dihydro-cinnamaldehyde (**5a**) quantification was performed on a Perkin Elmer Clarus 680 GC system coupled to a Perking Elmer Clarus SQ 8C quadrupole mass spectrometer in electron impact ionization mode. The column used was a Perkin Elmer Elite-1 30 m (0.25 mm x 0.25 mm). The inlet temperature was set to 150 °C with a split ratio of 19:1. An injection volume of 1.0 µL was used. The temperature program started at 50 °C and held for 1 minute, then ramped up to 220 °C at 15 °C/min and held for 2 minutes. Data was analyzed using Perkin and Elmer TurboMass software. Conversion of substrate to product was determined from a linear standard calibration curve using various concentrations of dihydro-cinnamaldehyde **5a** (1-100 µM, **Fig S13**). Peak area integration was used for the quantitation.

Gas chromatography-flame ionization detection (GC-FID)

Similar to previously described procedure,[11] the GC-FID assay for dihydro-cinnamaldehyde (**5a**) quantification was established to measure product conversion of cinnamaldehyde (**4a**) to dihydro-cinnamaldehyde (**5a**) using an Agilent 7890A GC system equipped with a flame ionization detector and a Restek RE-bDEXsm 30 m column (0.32 mm x 0.25 mm). The inlet temperature was set to 200 °C with a split ratio of 10:1. An injection volume of 10 µL was used. The temperature program started at 80 °C and held for 2 minutes, then ramped up to 200 °C at 15 °C/min and held for 2 minutes. Data was analyzed using Agilent ChemStation software. Conversion of substrate to product was determined from a linear standard calibration curve built from various concentrations of dihydro-cinnamaldehyde **5a** (1-100 µM, **Fig S13**). Peak area integration was used for the quantitation.

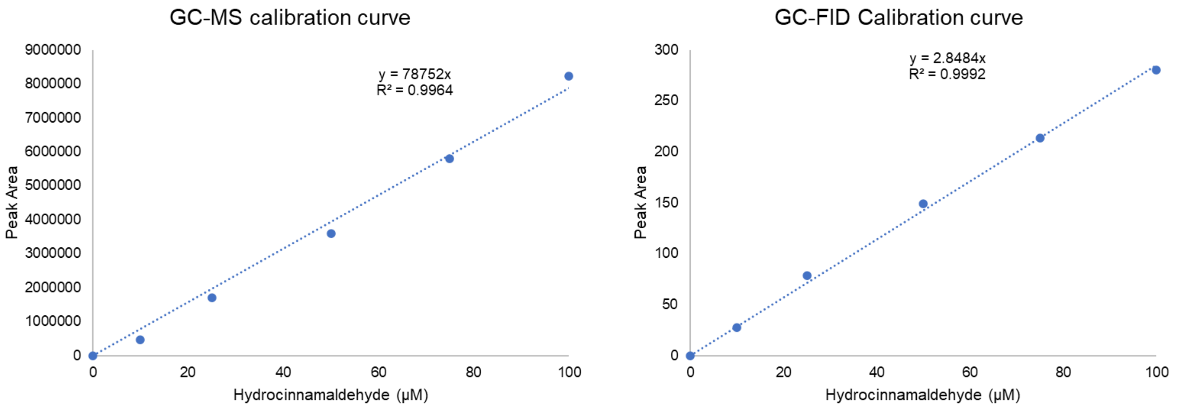

Figure S13. GC-MS and GC-FID calibration curve of dihydro-cinnamaldehyde (**5a**) which was used for estimation of product conversion.

Liquid chromatography-mass spectrometry (LC-MS)

Liquid chromatography-mass spectrometry (LC-MS) was acquired on a Waters Acquity H-Class UPLC system coupled to a Waters Synapt G2-Si quadrupole time of flight mass spectrometer. The column used was a Waters Acquity UPLC C18 BEH column 75 Å, 1.7 µm (2.1 x 100 mm) held at 40 °C. The flow rate was 0.3 mL/min and the gradient employed is highlighted in the **Table S10** below. Mass spectrometry data was collected in positive electrospray ionization mode and the data analyzed using Waters MassLynx 4.1.

Table S10. Liquid chromatography-mass spectrometry chromatography parameters.

Time (min)	H_2O (0.1% CHOOH)	ACN (0.1% CHOOH)
0	97	3
1	97	3
30	40	60
31	5	95
33	5	95
34	97	3
40	97	3

15. Nucleotide and amino acid sequences

MbPylRS

Nucleotide Sequence:

ATGGATAAAAAACCGCTGGATGTGCTGATTAGCGCGACCGGCCTGTGGATGAGCCGTACCGGCACCCTGCATAAAATCAAACATCATG
AAGTGAGCCGCAGCAAAATCTATATTGAAATGGCGTGCGGCGATCATCTGGTGGTGAACAACAGCCGTAGCTGCCGTACCGCGCGTGC
GTTTCGTCATCATAAATACCGCAAAACCTGCAAACGTTGCCGTGTGAGCGATGAAGATATCAACAACTTTCTGACCCGTAGCACCGAA
AGCAAAAACAGCGTGAAAGTGCGTGTGGTGAGCGCGCCGAAAGTGAAAAAAGCGATGCCGAAAAGCGTGAGCCGTGCGCCGAAACCGC
TGGAAAATAGCGTGAGCGCGAAAGCGAGCACCAACACCAGCCGTAGCGTTCCGAGCCCGGCGAAAAGCACCCCGAACAGCAGCGTTCC
GGCGTCTGCGCCGGCACCGAGCCTGACCCGCAGCCAGCTGGATCGTGTGGAAGCGCTGCTGTCTCCGGAAGATAAAATTAGCCTGAAC
ATGGCGAAACCGTTTCGTGAACTGGAACCGGAACTGGTGACCCGTCGTAAAAACGATTTTCAGCGCCTGTATACCAACGATCGTGAAG
ATTATCTGGGCAAACTGGAACGTGATATCACCAAATTTTTTGTGGATCGCGGCTTTCTGGAAATTAAAAGCCCGATTCTGATTCCGGC
GGAATATGTGGAACGTATGGGCATTAACAACGACACCGAACTGAGCAAACAAATTTTCCGCGTGGATAAAAACCTGTGCCTGCGTCCG
ATGCTGGCCCCGACCCTGTATAACTATCTGCGTAAACTGGATCGTATTCTGCCGGGTCCGATCAAAATTTTTGAAGTGGGCCCGTGCT
ATCGCAAAGAAAGCGATGGCAAAGAACACCTGGAAGAATTCACCATGGTTAACTTTTGCCAAATGGGCAGCGGCTGCACCCGTGAAAA
CCTGGAAGCGCTGATCAAAGAATTCCTGGATTATCTGGAAATCGACTTCGAAATTGTGGGCGATAGCTGCATGGTGTATGGCGATACC
CTGGATATTATGCATGGCGATCTGGAACTGAGCAGCGCGGTGGTGGGTCCGGTTAGCCTGGATCGTGAATGGGGCATTGATAAACCGT
GGATTGGCGCGGGTTTTGGCCTGGAACGTCTGCTGAAAGTGATGCATGGCTTCAAAAACATTAAACGTGCGAGCCGTAGCGAAAGCTA
CTATAACGGCATTAGCACGAACCTGTAA

Amino Acid Sequence:

MDKKPLDVLISATGLWMSRTGTLHKIKHHEVSRSKIYIEMACGDHLVVNNSRSCRTARAFRHHKYRKTCKRCRVSDEDINNFLTRSTE
SKNSVKVRVVSAPKVKKAMPKSVSRAPKPLENSVSAKASTNTSRSVPSPAKSTPNSSVPASAPAPSLTRSQLDRVEALLSPEDKISLN
MAKPFRELEPELVTRRKNDFQRLYTNDREDYLGKLERDITKFFVDRGFLEIKSPILIPAEYVERMGINNDTELSKQIFRVDKNLCLRP
MLAPTLYNYLRKLDRILPGPIKIFEVGPCYRKESDGKEHLEEFTMVNFCQMGSGCTRENLEALIKEFLDYLEIDFEIVGDSCMVYGDT
LDIMHGDLELSSAVVGPVSLDREWGIDKPWIGAGFGLERLLKVMHGFKNIKRASRSESYYNGISTNL

ThzKRS

Nucleotide Sequence:
ATGGATAAAAAACCGCTGGATGTGCTGATTAGCGCGACCGGCCTGTGGATGAGCCGTACCGGCACCCTGCATAAAATCAAACATCATG
AAGTGAGCCGCAGCAAAATCTATATTGAAATGGCGTGCGGCGATCATCTGGTGGTGAACAACAGCCGTAGCTGCCGTACCGCGCGTGC
GTTTCGTCATCATAAATACCGCAAAACCTGCAAACGTTGCCGTGTGAGCGGTGAAGATATCAACAACTTTCTGACCCGTAGCACCGAA
AGCAAAAACAGCGTGAAAGTGCGTGTGGTGAGCGCGCCGAAAGTGAAAAAAGCGATGCCGAAAAGCGTGAGCCGTGCGCCGAAACCGC
TGGAAAATAGCGTGGGCGCGAAAGCGAGCACCAACACCAGCCGTAGCGTTCCGAGCCCGGCGAAAAGCACCCCGAACAGCAGCGTTCC
GGCGTCTGCGCCGGCACCGAGCCTGACCCGCAGCCAGCTGGATCGTGTGGAAGCGCTGCTGTCTCCGGAAGATAAAATTAGCCTGAAC
ATGGCGAAACCGTTTCGTGAACTGGAACCGGAACTGGTGACCCGTCGTAAAAACGATTTTCAGCGCCTGTATACCAACGATCGTGAAG
ATTATCTGGGCAAACTGGAACGTGATATCACCAAATTTTTTGTGGATCGCGGCTTTCTGGAAATTAAAAGCCCGATTCTGATTCCGGC
GGAATATGTGGAACGTATGGGCATTAACAACGACACCGAACTGAGCAAACAAATTTTCCGCGTGGATAAAAACCTGTGCCTGCGTCCG
ATGCTGAGCCCGACCCTGTATAACTATCTGCGTAAACTGGATCGTATTCTGCCGGGTCCGATCAAAATTTTTGAAGTGGGCCCGTGCT
ATCGCAAAGAAAGCGATGGCAAAGAACACCTGGAAGAATTCACCATGGTTAACTTTGTGCAATTTGGCAGCGGCTGCACCCGTGAAAA
CCTGGAAGCGCTGATCAAAGAATTCCTGGATTATCTGGAAATCGACTTCGAAATTGTGGGCGGTAGCTGCATGGTGTATGGCGATACC
CTGGATATTATGCATGGCGATCTGGAACTGAGCAGCGCGGTGGTGGGTCCGGTTAGCCTGGATCGTGAATGGGGCATTGATAAACCGT
GGATTGGCGCGGGTTTTGGCCTGGAACGTCTGCTGAAAGTGATGCATGGCTTCAAAAACATTAAACGTGCGAGCCGTAGCGAAAGCTA
CTATAACGGCATTAGCACGAACCTGTAA

Amino Acid Sequence:
MDKKPLDVLISATGLWMSRTGTLHKIKHHEVSRSKIYIEMACGDHLVVNNSRSCRTARAFRHHKYRKTCKRCRVSGEDINNFLTRSTE
SKNSVKVRVVSAPKVKKAMPKSVSRAPKPLENSVGAKASTNTSRSVPSPAKSTPNSSVPASAPAPSLTRSQLDRVEALLSPEDKISLN
MAKPFRELEPELVTRRKNDFQRLYTNDREDYLGKLERDITKFFVDRGFLEIKSPILIPAEYVERMGINNDTELSKQIFRVDKNLCLRP
MLSPTLYNYLRKLDRILPGPIKIFEVGPCYRKESDGKEHLEEFTMVNFVQFGSGCTRENLEALIKEFLDYLEIDFEIVGGSCMVYGDT
LDIMHGDLELSSAVVGPVSLDREWGIDKPWIGAGFGLERLLKVMHGFKNIKRASRSESYYNGISTNL

MbPyl-tRNA

GGGAACCTGATCATGTAGATCGAATGGACTCTAAATCCGTTCAGCCGGGTTAGATTCCCGGGGTTTCCGCCA

Wild-type LmrR

Nucleotide Sequence:
ATGGGTGCCGAAATCCCGAAAGAAATGCTGCGTGCTCAAACCAATGTCATCCTGCTGAATGTCCTGAAACAAGGCGATAACTATGTGT
ATGGCATTATCAAACAGGTGAAAGAAGCGAGCAACGGTGAAATGGAACTGAATGAAGCCACCCTGTATACGATTTTTGATCGTCTGGA
ACAGGACGGCATTATCAGCTCTTACTGGGGTGATGAAAGTCAAGGCGGTCGTCGCAAATATTACCGTCTGACCGAAATCGGCCATGAA
AACATGCGCCTGGCGTTCGAATCCTGGAGTCGTGTGGACAAAATCATTGAAAATCTGGAAGCAAACAAAAAATCTGAAGCGATCAAAG
GCAGCAGCCATCATCATCATCATCACAGCAGCGGCTAA

Amino Acid Sequence:
MGAEIPKEMLRAQTNVILLNVLKQGDNYVYGIIKQVKEASNGEMELNEATLYTIFDRLEQDGIISSYWGDESQGGRRKYYRLTEIGHE
NMRLAFESWSRVDKIIENLEANKKSEAIKGSSHHHHHHSSG

LmrR-Val15-TAG

Nucleotide Sequence:
ATGGGTGCCGAAATCCCGAAAGAAATGCTGCGTGCTCAAACCAATTAGATCCTGCTGAATGTCCTGAAACAAGGCGATAACTATGTGT
ATGGCATTATCAAACAGGTGAAAGAAGCGAGCAACGGTGAAATGGAACTGAATGAAGCCACCCTGTATACGATTTTTGATCGTCTGGA
ACAGGACGGCATTATCAGCTCTTACTGGGGTGATGAAAGTCAAGGCGGTCGTCGCAAATATTACCGTCTGACCGAAATCGGCCATGAA
AACATGCGCCTGGCGTTCGAATCCTGGAGTCGTGTGGACAAAATCATTGAAAATCTGGAAGCAAACAAAAAATCTGAAGCGATCAAAG
GCAGCAGCCATCATCATCATCATCACAGCAGCGGCTAA

Amino Acid Sequence:
MGAEIPKEMLRAQTN*ILLNVLKQGDNYVYGIIKQVKEASNGEMELNEATLYTIFDRLEQDGIISSYWGDESQGGRRKYYRLTEIGHE
NMRLAFESWSRVDKIIENLEANKKSEAIKGSSHHHHHHSSG

LmrR-Asp19-TAG

Nucleotide Sequence:
ATGGGTGCCGAAATCCCGAAAGAAATGCTGCGTGCTCAAACCAATGTCATCCTGCTGTAGGTCCTGAAACAAGGCGATAACTATGTGT
ATGGCATTATCAAACAGGTGAAAGAAGCGAGCAACGGTGAAATGGAACTGAATGAAGCCACCCTGTATACGATTTTTGATCGTCTGGA
ACAGGACGGCATTATCAGCTCTTACTGGGGTGATGAAAGTCAAGGCGGTCGTCGCAAATATTACCGTCTGACCGAAATCGGCCATGAA
AACATGCGCCTGGCGTTCGAATCCTGGAGTCGTGTGGACAAAATCATTGAAAATCTGGAAGCAAACAAAAAATCTGAAGCGATCAAAG
GCAGCAGCCATCATCATCATCATCACAGCAGCGGCTAA

Amino Acid Sequence:
MGAEIPKEMLRAQTNVILL*VLKQGDNYVYGIIKQVKEASNGEMELNEATLYTIFDRLEQDGIISSYWGDESQGGRRKYYRLTEIGHE
NMRLAFESWSRVDKIIENLEANKKSEAIKGSSHHHHHHSSG

LmrR-Met89-TAG

Nucleotide Sequence:
ATGGGTGCCGAAATCCCGAAAGAAATGCTGCGTGCTCAAACCAATGTCATCCTGCTGAATGTCCTGAAACAAGGCGATAACTATGTGT
ATGGCATTATCAAACAGGTGAAAGAAGCGAGCAACGGTGAAATGGAACTGAATGAAGCCACCCTGTATACGATTTTTGATCGTCTGGA
ACAGGACGGCATTATCAGCTCTTACTGGGGTGATGAAAGTCAAGGCGGTCGTCGCAAATATTACCGTCTGACCGAAATCGGCCATGAA
AACTAGCGCCTGGCGTTCGAATCCTGGAGTCGTGTGGACAAAATCATTGAAAATCTGGAAGCAAACAAAAAATCTGAAGCGATCAAAG
GCAGCAGCCATCATCATCATCATCACAGCAGCGGCTAA

Amino Acid Sequence:
MGAEIPKEMLRAQTNVILLNVLKQGDNYVYGIIKQVKEASNGEMELNEATLYTIFDRLEQDGIISSYWGDESQGGRRKYYRLTEIGHE
N*RLAFESWSRVDKIIENLEANKKSEAIKGSSHHHHHHSSG

LmrR-Phe93-TAG

Nucleotide Sequence:
GGTGCCGAAATCCCGAAAGAAATGCTGCGTGCTCAAACCAATGTCATCCTGCTGAATGTCCTGAAACAAGGCGATAACTATGTGTATG
GCATTATCAAACAGGTGAAAGAAGCGAGCAACGGTGAAATGGAACTGAATGAAGCCACCCTGTATACGATTTTTGATCGTCTGGAACA
GGACGGCATTATCAGCTCTTACTGGGGTGATGAAAGTCAAGGCGGTCGTCGCAAATATTACCGTCTGACCGAAATCGGCCATGAAAAC
ATGCGCCTGGCGTAGGAATCCTGGAGTCGTGTGGACAAAATCATTGAAAATCTGGAAGCAAACAAAAAATCTGAAGCGATCAAAGGCA
GCAGCCATCATCATCATCATCACAGCAGCGGCTAA

Amino Acid Sequence:
MGAEIPKEMLRAQTNVILLNVLKQGDNYVYGIIKQVKEASNGEMELNEATLYTIFDRLEQDGIISSYWGDESQGGRRKYYRLTEIGHE
NMRLA*ESWSRVDKIIENLEANKKSEAIKGSSHHHHHHSSG

Wild-type DHFR

Nucleotide Sequence:
ATGATCAGTCTGATTGCGGCGTTAGCGGTAGATCGCGTTATCGGCATGGAAAACGCCATGCCGTGGAACCTGCCTGCCGATCTCGCCT
GGTTTAAACGCAACACCTTAAATAAACCCGTGATTATGGGCCGCCATACCTGGGAATCAATCGGTCGTCCGTTGCCAGGACGCAAAAA
TATTATCCTCAGCAGTCAACCGGGTACGGACGATCGCGTAACGTGGGTGAAGTCGGTGGATGAAGCCATCGCGGCGTGTGGTGACGTA
CCAGAAATCATGGTGATTGGCGGCGGTCGCGTTTATGAACAGTTCTTGCCAAAAGCGCAAAAACTGTATCTGACGCATATCGACGCAG
AAGTGGAAGGCGACACCCATTTCCCGGATTACGAGCCGGATGACTGGGAATCGGTATTCAGCGAATTCCACGATGCTGATGCGCAGAA
CTCTCACAGCTATTGCTTTGAGATTCTGGAGCGGCGGGGCAGCAGCCATCATCATCATCATCACAGCAGCGGCTAA

Amino Acid Sequence:
MISLIAALAVDRVIGMENAMPWNLPADLAWFKRNTLNKPVIMGRHTWESIGRPLPGRKNIILSSQPGTDDRVTWVKSVDEAIAACGDV
PEIMVIGGGRVYEQFLPKAQKLYLTHIDAEVEGDTHFPDYEPDDWESVFSEFHDADAQNSHSYCFEILERRGSSHHHHHHSSG

DHFR-Ala7-TAG

Nucleotide Sequence:
ATGATCAGTCTGATTGCGTAGTTAGCGGTAGATCGCGTTATCGGCATGGAAAACGCCATGCCGTGGAACCTGCCTGCCGATCTCGCCT
GGTTTAAACGCAACACCTTAAATAAACCCGTGATTATGGGCCGCCATACCTGGGAATCAATCGGTCGTCCGTTGCCAGGACGCAAAAA
TATTATCCTCAGCAGTCAACCGGGTACGGACGATCGCGTAACGTGGGTGAAGTCGGTGGATGAAGCCATCGCGGCGTGTGGTGACGTA
CCAGAAATCATGGTGATTGGCGGCGGTCGCGTTTATGAACAGTTCTTGCCAAAAGCGCAAAAACTGTATCTGACGCATATCGACGCAG
AAGTGGAAGGCGACACCCATTTCCCGGATTACGAGCCGGATGACTGGGAATCGGTATTCAGCGAATTCCACGATGCTGATGCGCAGAA
CTCTCACAGCTATTGCTTTGAGATTCTGGAGCGGCGGGGCAGCAGCCATCATCATCATCATCACAGCAGCGGCTAA

Amino Acid Sequence:
MISLIA*LAVDRVIGMENAMPWNLPADLAWFKRNTLNKPVIMGRHTWESIGRPLPGRKNIILSSQPGTDDRVTWVKSVDEAIAACGDV
PEIMVIGGGRVYEQFLPKAQKLYLTHIDAEVEGDTHFPDYEPDDWESVFSEFHDADAQNSHSYCFEILERRGSSHHHHHHSSG

DHFR-Phe31-TAG

Nucleotide Sequence:
ATGATCAGTCTGATTGCGGCGTTAGCGGTAGATCGCGTTATCGGCATGGAAAACGCCATGCCGTGGAACCTGCCTGCCGATCTCGCCT
GGTAGAAACGCAACACCTTAAATAAACCCGTGATTATGGGCCGCCATACCTGGGAATCAATCGGTCGTCCGTTGCCAGGACGCAAAAA
TATTATCCTCAGCAGTCAACCGGGTACGGACGATCGCGTAACGTGGGTGAAGTCGGTGGATGAAGCCATCGCGGCGTGTGGTGACGTA
CCAGAAATCATGGTGATTGGCGGCGGTCGCGTTTATGAACAGTTCTTGCCAAAAGCGCAAAAACTGTATCTGACGCATATCGACGCAG
AAGTGGAAGGCGACACCCATTTCCCGGATTACGAGCCGGATGACTGGGAATCGGTATTCAGCGAATTCCACGATGCTGATGCGCAGAA
CTCTCACAGCTATTGCTTTGAGATTCTGGAGCGGCGGGGCAGCAGCCATCATCATCATCATCACAGCAGCGGCTAA

Amino Acid Sequence:
MISLIAALAVDRVIGMENAMPWNLPADLAW*KRNTLNKPVIMGRHTWESIGRPLPGRKNIILSSQPGTDDRVTWVKSVDEAIAACGDV
PEIMVIGGGRVYEQFLPKAQKLYLTHIDAEVEGDTHFPDYEPDDWESVFSEFHDADAQNSHSYCFEILERRGSSHHHHHHSSG

DHFR-Ser49-TAG

Nucleotide Sequence:

ATGATCAGTCTGATTGCGGCGTTAGCGGTAGATCGCGTTATCGGCATGGAAAACGCCATGCCGTGGAACCTGCCTGCCGATCTCGCCT
GGTTTAAACGCAACACCTTAAATAAACCCGTGATTATGGGCCGCCATACCTGGGAATAGATCGGTCGTCCGTTGCCAGGACGCAAAAA
TATTATCCTCAGCAGTCAACCGGGTACGGACGATCGCGTAACGTGGGTGAAGTCGGTGGATGAAGCCATCGCGGCGTGTGGTGACGTA
CCAGAAATCATGGTGATTGGCGGCGGTCGCGTTTATGAACAGTTCTTGCCAAAAGCGCAAAAACTGTATCTGACGCATATCGACGCAG
AAGTGGAAGGCGACACCCATTTCCCGGATTACGAGCCGGATGACTGGGAATCGGTATTCAGCGAATTCCACGATGCTGATGCGCAGAA
CTCTCACAGCTATTGCTTTGAGATTCTGGAGCGGCGGGGCAGCAGCCATCATCATCATCATCACAGCAGCGGCTAA

Amino Acid Sequence:

MISLIAALAVDRVIGMENAMPWNLPADLAWFKRNTLNKPVIMGRHTWE*IGRPLPGRKNIILSSQPGTDDRVTWVKSVDEAIAACGDV
PEIMVIGGGRVYEQFLPKAQKLYLTHIDAEVEGDTHFPDYEPDDWESVFSEFHDADAQNSHSYCFEILERRGSSHHHHHHSSG

16. Computational Studies

System setup.

A model of *Escherichia coli* dihydrofolate reductase (DHFR) with folate (FOL) and oxidized form of nicotinamide adenine dinucleotide phosphate ($NADP^+$) was built based on the crystal structure available in the Protein Data Bank (PDB ID:1RX2).[17] Subsequently, the folate molecule (FOL) was removed from the structure, and cofactor $NADP^+$ was modified to its reduced form (NADPH). Ala7 in proximity to the nicotinamide motif was replaced with the non-hydrolyzable unnatural amino acid L-prolyl-L-lysine (**1**), yielding **DHFR-Ala7-1**. The carbon atom of (*E*)-3-phenyl-2-butenal (**4i**) was covalently attached to secondary amine of the residue of **1**, in its iminium ion form (**Table S11**). Amber force field(FF)[18] parameters for NADPH were adapted from the work of Bulow and co-workers.[19] Missing FF parameters for iminium ion were generated employing the Generalized Amber Force Field (GAFF)[20] and the atomic charges were computed using the AM1 method with bond charge corrections (AM1-BCC)[21] using the Antechamber software.[22] All newly produced parameters are provided in **Table S11**. Since stereoselectivity was experimentally found to be enhanced with increasing pH whilst showing no enantiomeric excess under acidic conditions (pH 4.0), the **DHFR-Ala7-1** was modelled at pH 11.0. The pK_a shift of all titratable residues was determined with PropKa software ver. 3.1.[23, 24] Titratable curves were derived under hybrid non-equilibrium molecular dynamic and Monte Carlo (*ne*MD/MC) simulations[25] with Tcl plugin, namdcph, used in conjunction with NAMD ver. 2.12.[26] All *ne*MD/MC simulations were carried out with the CHARMM36 force field.[27] Constant-pH MD assays of the titration curves were performed on 74 pH values between 0.2 and 14.8 (0.2 unit interval) and repeated five times. All simulations attempted protonation moves every 10 ps over 50 ns with switch times of 20 ps (i.e., 5000 neMD/MC cycles). The efficiency of sampling was improved by assigning inherent pK_a values using the ones originally predicted by PropKa software. The protonation states of Asp, Glu, His, Cys, and Lys were explored during these simulations yielding complete titratable curves **(Fig. S14** and **Table S12**). Since neMD/MC method is not yet capable of predicting pK_a's of Tyr residues, the values of those were estimated using propKa software. The pK_a values for all tyrosine residues present in DHFR protein were found to be higher than 11 (Tyr100 (14.05), Tyr111 (13.5), Tyr128 (11.56), and Tyr151 (11.23)), and hence they were not deprotonated in the final model. However, according to computed pK_a values, all Cys and Lys residues presented in the system were deprotonated at pH 11.0. All remaining residues were found in their natural protonated state. After geometrical inspection, His45 and His149 were protonated in δ, while His114, His124, and His141 were in ε-position. The missing hydrogen atoms in the enzyme, cofactor and substrate were replaced with 22 positively charged sodium (Na^+) counterions to create an electrostatically neutral and stable complex. Hydrogen and counterions were added using the tLEAP[28] module of the AmberTools package. Subsequently, the system was soaked within an orthorhombic box of TIP3P[29] water molecules, with an average size of 70 × 74 × 68 Å^3. To describe the protein and water molecules, the AMBER ff03.r1 and TIP3P force fields were employed, respectively, and the NAMD software was used as a molecular dynamic (MD) engine.

Table S11. Atom types, AM1-bcc charges (in a.u.), and parameters obtained for (*E*)-3-phenyl-2-butenal (**4i**) covalently attached to an unnatural amino acid, 1, in its iminium ion based on GAFF force field.

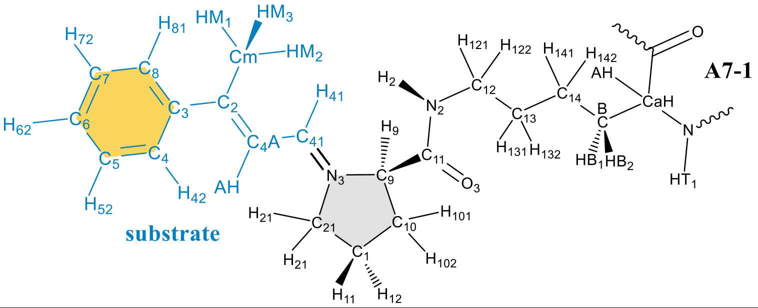

Atom name	Atom type	Charge	Atom name	Atom type	Charge
N	n3	-0.854550	C7	ca	-0.126575
HT1	hn	0.374313	C8	ca	-0.077004
CA	c3	0.058616	H81	ha	0.145243
CB	c3	-0.073539	H72	ha	0.161324
C14	c3	-0.076426	H62	ha	0.162880
C13	c3	-0.099527	H52	ha	0.161324
C12	c3	0.079884	H42	ha	0.145243
N2	n	-0.512942	CM	c3	-0.119260
C11	c	0.673410	HM1	hc	0.087180
C9	c3	-0.066416	HM2	hc	0.087180
N3	nh	-0.145441	HM3	hc	0.087180
C21	c3	-0.007412	H3	ha	0.168067
C1	c3	-0.089902	H41	h4	0.080714
C10	c3	-0.089902	H9	h1	0.112771
H101	hc	0.086835	O3	o	-0.594951
H102	hc	0.086835	H2	hn	0.332504
H11	hc	0.089428	H121	h1	0.079573
H12	hc	0.089428	H122	h1	0.079573
H211	h1	0.093578	H131	hc	0.053118
H212	h1	0.093578	H132	hc	0.053118
C41	ce	0.466335	H141	hc	0.050524
C4A	ce	-0.394259	H142	hc	0.050524
C2	cf	0.200851	HB1	hc	0.061417
C3	ca	-0.143227	HB2	hc	0.061417
C4	ca	-0.077004	HA	h1	0.083722
C5	ca	-0.126575	C	c	0.540409
C6	ca	-0.068341	O	o	-0.494846

**MASS**			**DIHEDRAL**				
n3	14.010	0.530	c3-c3-c3-n3	9	1.400	0.000	3.000
hn	1.008	0.161	hc-c3-c3-n3	9	1.400	0.000	3.000
c3	12.010	0.878	o -c -c3-n3	6	0.000	180.000	2.000
n	14.010	0.530	c3-c3-n3-hn	6	1.800	0.000	3.000
c	12.010	0.616	h1-c3-n3-hn	6	1.800	0.000	3.000
nh	14.010	0.530	c -c3-n3-hn	6	1.800	0.000	3.000
hc	1.008	0.135	c3-c3-c3-c3	1	0.180	0.000	-3.000
h1	1.008	0.135	c3-c3-c3-c3	1	0.250	180.000	-2.000
ce	12.010	0.360	c3-c3-c3-c3	1	0.200	180.000	1.000
cf	12.010	0.360	c3-c3-c3-hc	1	0.160	0.000	3.000
ca	12.010	0.360	o -c -c3-c3	6	0.000	180.000	2.000
ha	1.008	0.135	c3-c3-c3-n	9	1.400	0.000	3.000
h4	1.008	0.135	c3-c3-c3-h1	9	1.400	0.000	3.000
o	16.000	0.434	c3-c3-n -c	1	0.500	180.000	-4.000
**BOND**			c3-c3-n -c	1	0.150	180.000	-3.000
hn-n3	392.40	1.019	c3-c3-n -c	1	0.000	0.000	-2.000
c3-n3	325.90	1.465	c3-c3-n -c	1	0.530	0.000	1.000
c3-c3	300.90	1.538	c3-c3-n -hn	6	0.000	0.000	2.000
c3-h1	330.60	1.097	c3-c -n -c3	1	0.000	0.000	-2.000
c -c3	313.00	1.524	c3-c -n -c3	1	1.500	180.000	1.000
c3-hc	330.60	1.097	o -c -n -c3	4	10.000	180.000	2.000
c3-n	328.70	1.462	n -c -c3-nh	6	0.000	180.000	2.000
c -n	427.60	1.379	n -c -c3-c3	1	0.100	0.000	-4.000
hn-n	403.20	1.013	n -c -c3-c3	1	0.070	0.000	2.000

c -o	637.70	1.218	n -c -c3-h1	6	0.000	180.000	2.000
c3-nh	326.60	1.464	c -c3-nh-c3	6	0.000	0.000	2.000
ce-nh	412.30	1.390	c -c3-nh-ce	6	0.000	0.000	2.000
ce-ce	382.80	1.457	c -c3-c3-c3	9	1.400	0.000	3.000
ce-h4	337.80	1.092	c -c3-c3-hc	9	1.400	0.000	3.000
ce-cf	538.60	1.351	c3-c3-nh-c3	6	0.000	0.000	2.000
ca-ca	342.50	1.088	h1-c3-nh-c3	6	0.000	0.000	2.000
ca-ha	361.30	1.476	ce-ce-nh-c3	4	4.200	180.000	2.000
c -N	320.90	1.516	h4-ce-nh-c3	4	4.200	180.000	2.000
C -n3	461.10	1.398	c3-c3-c3-nh	9	1.400	0.000	3.000
**ANGLE**			hc-c3-c3-nh	9	1.400	0.000	3.000
c3-c3-n3	66.000	111.040	cf-ce-ce-nh	4	4.000	180.000	2.000
h1-c3-n3	49.500	109.880	ha-ce-ce-nh	4	4.000	180.000	2.000
c -c3-n3	66.300	111.140	c3-c3-nh-ce	6	0.000	0.000	2.000
c3-n3-hn	47.400	109.290	hc-c3-c3-hc	1	0.150	0.000	3.000
c3-c3-c3	62.900	111.510	h1-c3-c3-hc	9	1.400	0.000	3.000
c3-c3-hc	46.300	109.800	h1-c3-nh-ce	6	0.000	0.000	2.000
c3-c -o	67.400	123.200	ce-ce-cf-ca	4	26.600	180.000	2.000
c3-c3-h1	46.400	109.560	ce-ce-cf-c3	4	26.600	180.000	2.000
c -c3-c3	63.300	111.040	ca-ca-cf-ce	4	2.800	180.000	2.000
c3-c3-n	65.900	111.610	hc-c3-cf-ce	1	0.380	180.000	-3.000
c -n -c3	63.400	120.690	hc-c3-cf-ce	1	0.000	0.000	-2.000
c3-n -hn	45.800	117.680	hc-c3-cf-ce	1	1.150	0.000	1.000
h1-c3-n	49.800	108.880	ca-ca-ca-cf	4	14.500	180.000	2.000
c3-c -n	66.800	115.180	cf-ca-ca-ha	4	14.500	180.000	2.000
n -c -o	74.200	123.050	hc-c3-cf-ca	1	0.380	180.000	-3.000
c -n -hn	48.300	117.550	hc-c3-cf-ca	1	0.000	0.000	-2.000
c -c3-nh	66.900	109.350	hc-c3-cf-ca	1	1.150	0.000	1.000
c -c3-h1	47.000	108.220	ca-ca-ca-ca	4	14.500	180.000	2.000
c3-nh-c3	63.200	114.510	ca-ca-ca-ha	4	14.500	180.000	2.000
c3-nh-ce	63.300	120.120	ha-ca-ca-ha	4	14.500	180.000	2.000
c3-c3-nh	66.200	110.460	ca-ca-cf-c3	4	2.800	180.000	2.000
h1-c3-nh	49.600	109.790	ha-ce-cf-ca	4	26.600	180.000	2.000
ce-ce-nh	68.000	116.410	ha-ce-cf-c3	4	26.600	180.000	2.000
h4-ce-nh	50.500	115.580	cf-ce-ce-h4	4	4.000	180.000	2.000
hc-c3-hc	39.400	107.580	h4-ce-ce-ha	4	4.000	180.000	2.000
h1-c3-h1	39.200	108.460	o -c -c3-nh	6	0.000	180.000	2.000
ce-ce-cf	65.000	124.240	o -c -c3-h1	1	0.800	0.000	-1.000
ce-ce-ha	47.200	116.650	o -c -c3-h1	1	0.000	0.000	-2.000
ce-ce-h4	46.900	118.130	o -c -c3-h1	1	0.080	180.000	3.000
ca-cf-ce	63.700	127.520	c3-c -n -hn	4	10.000	180.000	2.000
c3-cf-ce	63.900	122.380	o -c -n -hn	1	2.500	180.000	-2.000
cf-ce-ha	49.800	118.220	o -c -n -hn	1	2.000	0.000	1.000
ca-ca-cf	64.500	120.820	h1-c3-n -c	6	0.000	0.000	2.000
cf-c3-hc	46.800	110.590	h1-c3-n -hn	6	0.000	0.000	2.000
c3-cf-ca	62.500	119.240	hc-c3-c3-n	9	1.400	0.000	3.000
ca-ca-ca	66.600	120.020	o -c -N -H	1	2.500	180.000	-2.000
ca-ca-ha	48.200	119.880	o -c -N -H	1	2.000	0.000	1.000
N -c -o	74.200	123.050	O -C -n3-hn	1	2.500	180.000	-2.000
c -N -H	48.300	117.550	O -C -n3-hn	1	2.000	0.000	1.000
c -N -CT	63.400	120.690	O -C -n3-c3	4	10.000	180.000	2.000
N -c -os	74.300	112.820	c3-c -N -H	4	10.000	180.000	2.000
n3-C -O	74.300	112.820	CT-C -n3-hn	4	10.000	180.000	2.000
C -n3-hn	48.300	117.550	n3-c3-c -N	1	1.700	180.000	-1.
C -n3-c3	63.400	120.690	n3-c3-c -N	1	2.000	180.000	2.
CT-C -n3	70.0	116.60	o -c -N-CT	4	10.000	180.000	2.000
c3-c -N	70.0	116.60	c3-c3-c -N	1	0.100	0.0	-4.
**NONBON**			c3-c3-c -N	1	0.07	0.0	2.

n3	1.8240	0.1700	C -n3-c3-c	1	0.850	180.000	-2.
hn	0.6000	0.0157	C -n3-c3-c	1	0.800	0.000	1.
c3	1.9080	0.1094	c3-c3-n3-C	1	0.50	180.0	-4.
n	1.8240	0.1700	c3-c3-n3-C	1	0.15	180.0	-3.
c	1.9080	0.0860	c3-c3-n3-C	1	0.00	0.0	-2.
nh	1.8240	0.1700	c3-c3-n3-C	1	0.53	0.0	1.
hc	1.4870	0.0157	C -n3-c3-h1	1	0.156	0.000	3.000
h1	1.3870	0.0157	X -C -n3-X	4	10.00	180.0	2.
ce	1.9080	0.0860	X -c -N -X	4	10.00	180.0	2.
cf	1.9080	0.0860	X -c -c3-X	6	0.00	0.0	2.
ca	1.9080	0.0860	**IMPROPER**				
ha	1.4590	0.0150	c -c3-n -hn	1.1		180.0	2.0
h4	1.4090	0.0150	c3-n -c -o	10.5		180.0	2.0
o	1.6612	0.2100	ce-h4-ce-nh	1.1		180.0	2.0
			ce-cf-ce-ha	1.1		180.0	2.0
			c3-ca-cf-ce	1.1		180.0	2.0
			ca-ca-ca-cf	1.1		180.0	2.0
			ca-ca-ca-ha	1.1		180.0	2.0

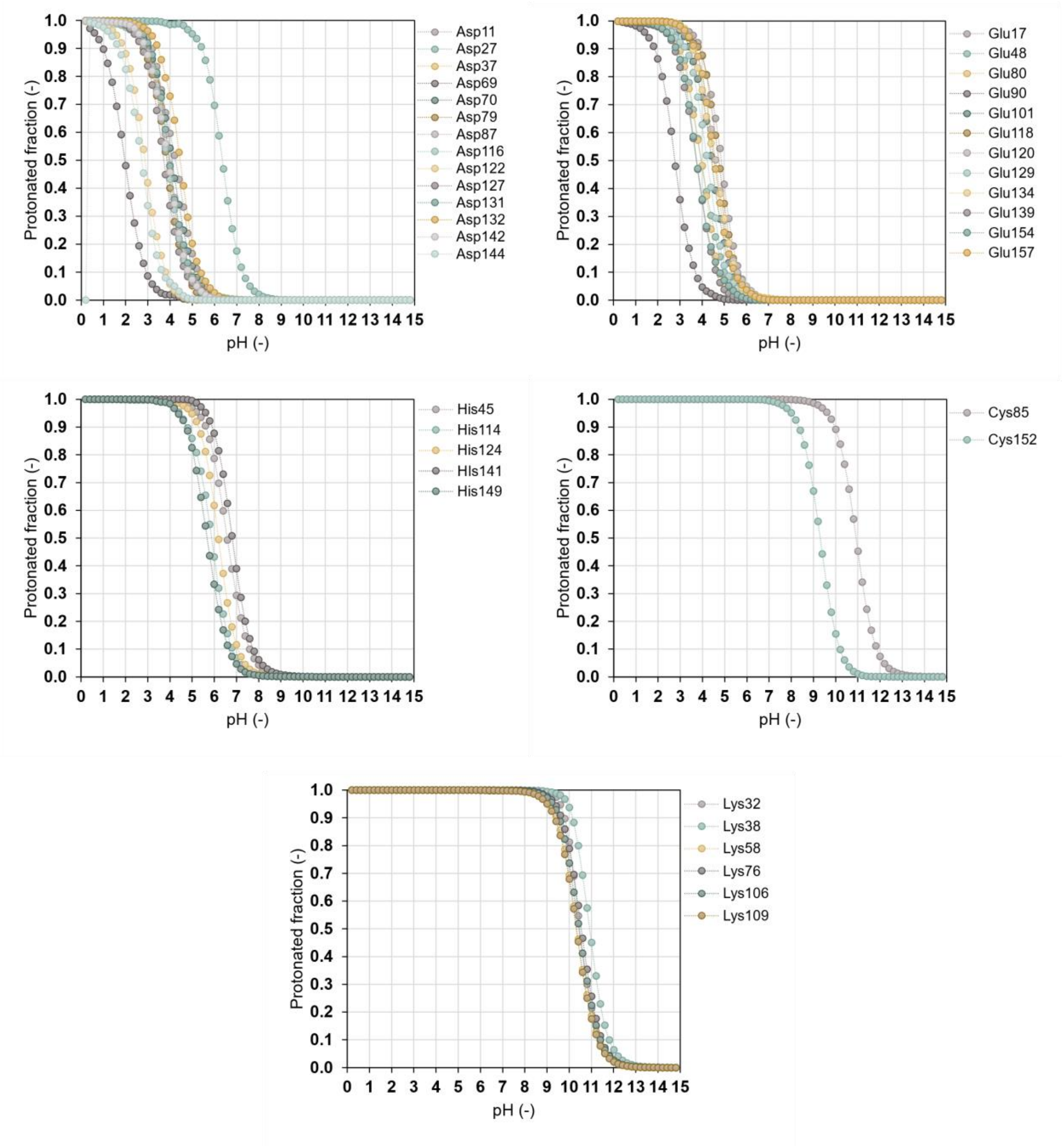

Figure S14. Computed titration curves for Asp, Glu, His, Cys and Lys residues of **DHFR-Ala7-1** using *ne*MD/MC.

Table S12. Assigned pH-dependent protonation state of titratable residues in **DHFR-Ala7-1** according to computed pK_a values.

**Asp**	pK_a $^{(a)}$	pK_a $^{(b)}$	pH							
			4	5	6	7	8	9	10	11
11	3.88	4.210 ± 0.016	+	-	-	-	-	-	-	-
27	6.33	6.350 ± 0.002	+	+	+	-	-	-	-	-
37	3.92	3.935 ± 0.012	-	-	-	-	-	-	-	-
69	1.78	1.768 ± 0.006	-	-	-	-	-	-	-	-
70	4.04	4.079 ± 0.012	+	-	-	-	-	-	-	-
79	3.80	3.798 ± 0.014	-	-	-	-	-	-	-	-
87	3.87	3.945 ± 0.007	-	-	-	-	-	-	-	-
116	3.91	3.853 ± 0.013	-	-	-	-	-	-	-	-
122	2.95	2.829 ± 0.015	-	-	-	-	-	-	-	-
127	3.75	3.737 ± 0.020	-	-	-	-	-	-	-	-
131	3.96	3.985 ± 0.012	-	-	-	-	-	-	-	-
132	4.42	4.405 ± 0.020	+	-	-	-	-	-	-	-
142	3.99	3.873 ± 0.016	-	-	-	-	-	-	-	-
144	2.55	2.492 ± 0.010	-	-	-	-	-	-	-	-

(a) pK_a values predicted using the PropKa program; (b) pK_a values determined based on computed titration curves using the *ne*MD/MC method.

Glu	pK_a $^{(a)}$	pK_a $^{(b)}$	pH							
			4	5	6	7	8	9	10	11
17	4.84	4.838 ± 0.005	+	-	-	-	-	-	-	-
48	4.06	4.020 ± 0.013	+	-	-	-	-	-	-	-
80	3.91	3.891 ± 0.012	-	-	-	-	-	-	-	-
90	2.56	2.565 ± 0.007	-	-	-	-	-	-	-	-
101	4.42	4.409 ± 0.009	+	-	-	-	-	-	-	-
118	4.67	4.732 ± 0.011	+	-	-	-	-	-	-	-
120	4.56	4.613 ± 0.008	+	-	-	-	-	-	-	-
129	4.16	4.210 ± 0.014	+	-	-	-	-	-	-	-
134	4.48	4.572 ± 0.015	+	-	-	-	-	-	-	-
139	3.78	3.692 ± 0.016	-	-	-	-	-	-	-	-
154	3.79	3.778 ± 0.009	-	-	-	-	-	-	-	-
157	4.64	4.569 ± 0.010	+	-	-	-	-	-	-	-

(a) pK_a values predicted using the PropKa program; (b) pK_a values determined based on computed titration curves using the *ne*MD/MC method.

His	pK_a $^{(a)}$	pK_a $^{(b)}$	pH							
			4	5	6	7	8	9	10	11
45	6.91	6.596 ± 0.007	+	+	+	-	-	-	-	-
114	6.16	5.863 ± 0.005	+	+	-	-	-	-	-	-
124	6.48	6.189 ± 0.001	+	+	+	-	-	-	-	-
141	6.88	6.619 ± 0.003	+	+	+	-	-	-	-	-
149	6.03	5.684 ± 0.002	+	+	-	-	-	-	-	-

(a) pK_a values predicted using the PropKa program; (b) pK_a values determined based on computed titration curves using the *ne*MD/MC method.

Cys	pK_a $^{(a)}$	pK_a $^{(b)}$	pH							
			4	5	6	7	8	9	10	11
85	10.86	10.914 ± 0.001	+	+	+	+	+	+	+	-
152	9.35	9.294 ± 0.003	+	+	+	+	+	+	-	-

(a) pK_a values predicted using the PropKa program; (b) pK_a values determined based on computed titration curves using the *ne*MD/MC method.

Lys	pK_a $^{(a)}$	pK_a $^{(b)}$	pH							
			4	5	6	7	8	9	10	11
32	9.91	10.505 ± 0.008	+	+	+	+	+	+	+	-
38	10.53	10.932 ± 0.004	+	+	+	+	+	+	+	-
58	10.3	10.351 ± 0.002	+	+	+	+	+	+	+	-
76	10.36	10.348 ± 0.001	+	+	+	+	+	+	+	-
106	10.51	10.449 ± 0.003	+	+	+	+	+	+	+	-
109	10.28	10.321 ± 0.001	+	+	+	+	+	+	+	-

(a) pK_a values predicted using the PropKa program; (b) pK_a values determined based on computed titration curves using the *ne*MD/MC method.

Molecular dynamic simulations.

For this study, two DHFR models at pH 11.0 were prepared for MD simulations. In these models, the prochiral iminium ion, covalently linked to the residue of **1**, was oriented in two different orientations: in one, the pro-*S* face was directed towards the nicotinamide motif, and in the other, the pro-*R* face was.

After initial energy minimizations, both systems were heated to 277 K with 0.1 K temperature increment and equilibrated during short (500 ps) NPT MD simulations, followed by non-accelerated classical 500 ns NPT MD simulations with AMBER force field,[30] as implemented in NAMD software. A cut-off for non-bonding interactions was set between 14.5 to 16 Å using a smooth switching function. The temperature (277 K) during the simulations was controlled using the Langevin thermostat,[31] and the pressure (1 bar) with the Nosé-Hoover Langevin piston[32] pressure control. In all simulations,

periodic boundary conditions (PBC) were applied. The geometrical analysis of the trajectories generated during the MD simulations was done using CPPTRAJ[33] as implemented in AmberTools.[34] In order to control the position of the substituents of C_β of the substrate, and hence possible selectivity towards formation of the *S*- or *R*-product, the specific dihedral angle (defined by the position of four carbon atoms, i.e., C4 of the nicotinamide ring of NADPH, C_β and C_α of substrate and carbon atom of the methyl group -CH_3 attached to C_β, as illustrated in **Fig. S15**) was used and monitored during MD simulations. The negative value of this dihedral close to 80 degrees promotes the formation of *R*-enantiomer, while its positive value would support the formation of *S*-product.

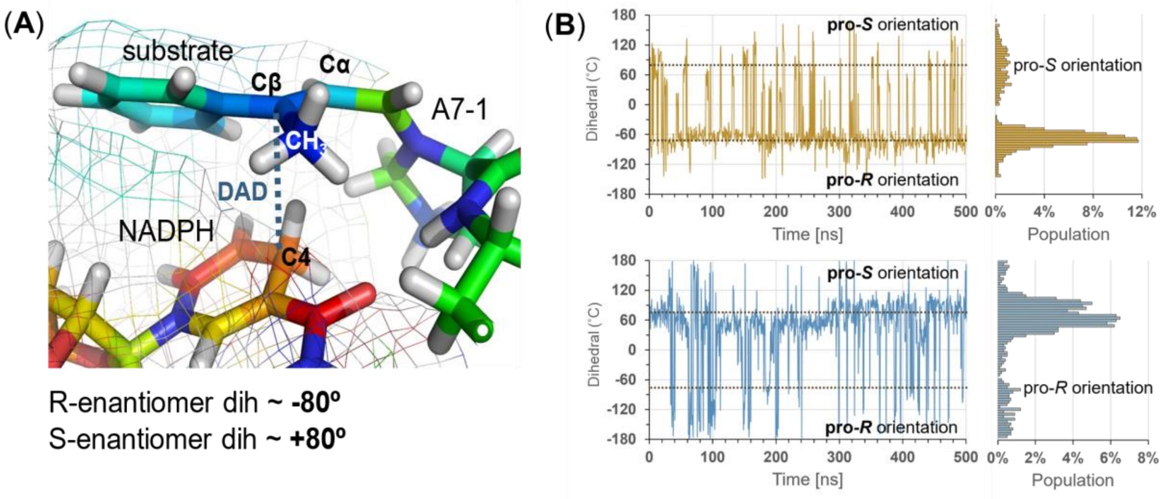

Figure S15. (**A**) The relative position of NADPH and the iminium ion intermediate (in this case facilitating the formation of *S*-enantiomer product) within the active site of **DHFR-Ala7-1**. Positions of atoms defining the key dihedral angle were indicated. (**B**) Evolution of dihedral (C4NADPH-C_β-C_α-CH3) angle during 500 ns of unbiased MM MD simulations and population analysis.

QM/MM study.

In the present work, the standard additive hybrid QM/MM scheme was used to construct the total Hamiltonian, $\hat{H}_{QM/MM}$, where the total energy $E_{QM/MM}$ is obtained as the sum of specific contributions, as presented in the following equation:

$$E_{QM/MM} = \langle \Psi | \widehat{H_o} | \Psi \rangle + \left(\sum \left\langle \Psi \middle| \frac{q_{MM}}{r_{e,MM}} \middle| \Psi \right\rangle + \sum \sum \frac{Z_{QM} q_{MM}}{r_{QM,MM}} \right) + E_{QM/MM}^{vdW} + E_{MM}$$

where $E_{QM/MM}$ is the energy of the MM subsystem term, $E_{QM-MM}^{vdW}$the van der Waals interaction energy between the QM and MM subsystems and E_{QM-MM}^{elect} includes both the Coulombic interaction of the QM nuclei (Z_{QM}) and the electrostatic interaction of the polarized electronic wave function with the charges of the protein (q_{MM}). The region described by quantum mechanics includes the side chain of Ala7-1 residue as well as the full substrate, as shown in **Fig. S16** and **S17**.

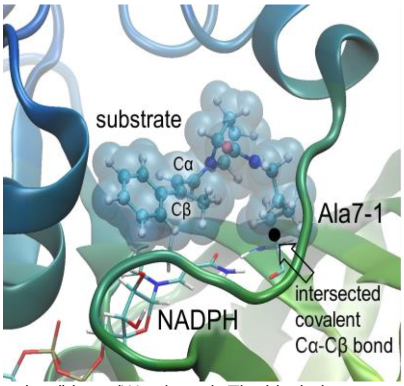

Figure S16. Representation of the QM sub-set region (blue vdW spheres). The black dot represents the position of the link atom between the QM and MM regions.

One link atom was inserted where the QM/MM boundary intersected covalent bonds, placed between the C_α-C_β of Ala7-1. The AM1[35] semiempirical Hamiltonian was used to treat the QM sub-set of atoms corresponding to the substrate as implemented in Mopac.[36] The Amber and TIP3P classical force fields were used to treat the protein and the solvent water molecules, respectively, as implemented in the fDynamo library.[37, 38] The atom positions of all residues presented beyond 20 Å from the substrate were frozen and the same cut-offs as in MD simulations were applied for the nonbonding interactions. A first minimization of the full system was done using a combination of conjugate gradient and L-BFGS-B algorithms implemented in the fDynamo library. Potential Energy Surfaces (PES) were explored by choosing starting value and scanning the dihedral angle ($C4^{NADPH}$-C_β-C_α-CH_3) as internal coordinate assuming its dominant role in the interchange of substrate from pro-*R* to pro-*S* orientation. The value of the dihedral was changed in the range from -140 to 140°. The harmonic constraint of 10 $kJ{\cdot}mol^{-1}{\cdot}deg^{-2}$ was used to maintain the proper angle along the rotation process, and a series of conjugate gradient optimizations and L-BFGS-B optimization algorithms were applied to obtain the final potential energy of the minimized constrained geometry. The dihedral evolution was controlled by applying a small size change of 2°. Free energy surface (FES) was obtained for every step of the reaction using the Umbrella Sampling (US) approach[39, 40] combined with the Weighted Histogram Analysis Method (WHAM).[41] Potential mean force (PMF) calculation is straightforward and requires a series of MD simulations (in this case 141 windows) in which the distinguished reaction coordinate variable, ξ, is constrained around particular values. The values of the variables sampled during the simulations are then pieced together to construct a distribution function from which the PMF is obtained as a function of the distinguished reaction coordinate, W(ξ). Details of this method has been reported in our previous studies.[42] MD simulations were performed adding a constraint for the selected reaction coordinates with an umbrella force constant of 1 $kJ{\cdot}mol^{-1}{\cdot}deg^{-2}$. In every window, QM/MM MD simulations were performed with a total of 5 ps of equilibration and 100 ps of production at 277 K using the Langevin-Verlet algorithm with a time step of 1 fs. Structures obtained in previously computed PESs were used as starting points for the MD simulations in every window.

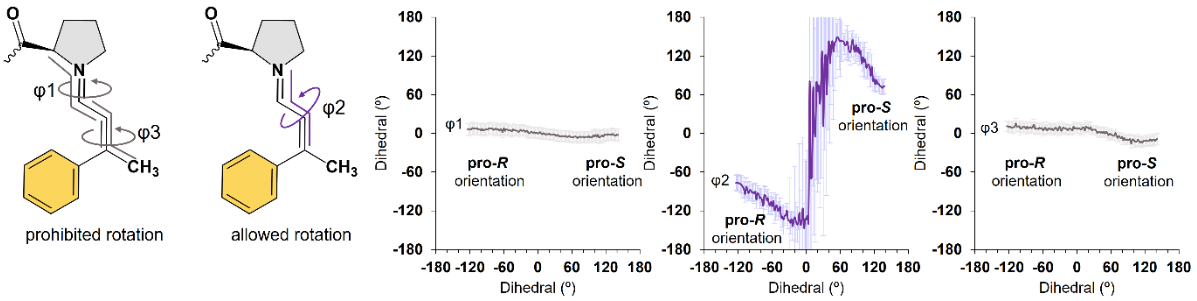

Figure S17. Evolution of the key dihedral angles within the iminium ion intermediate resulting in a change in the pro-*R* to pro-*S* orientation within the active site of **DHFR-Ala7-1** from Umbrella Sampling simulations at AM1/MM level of theory.

17. NMR spectroscopy and Mass spectrometry

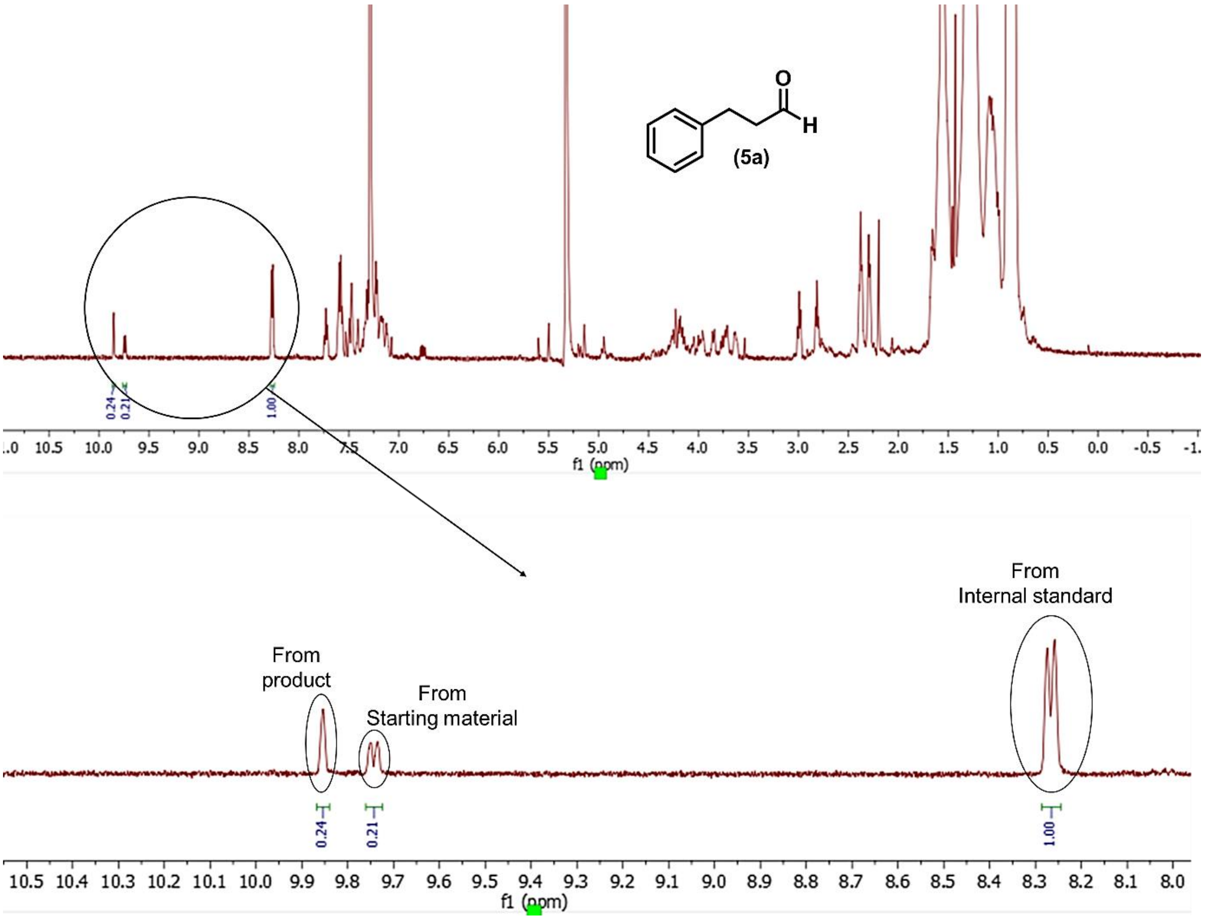

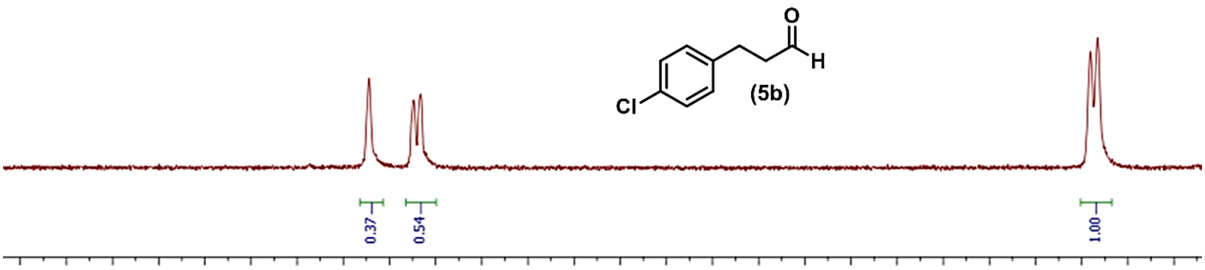

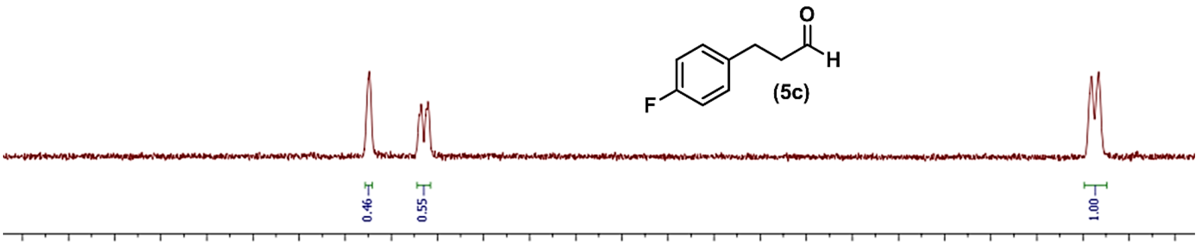

F
O
H
(5c)
0.46
0.55
1.00

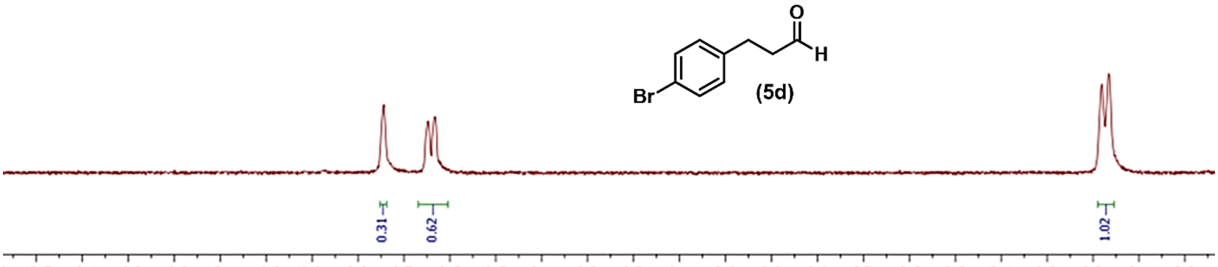

Br
O
H
(5d)
0.31
0.62
1.02

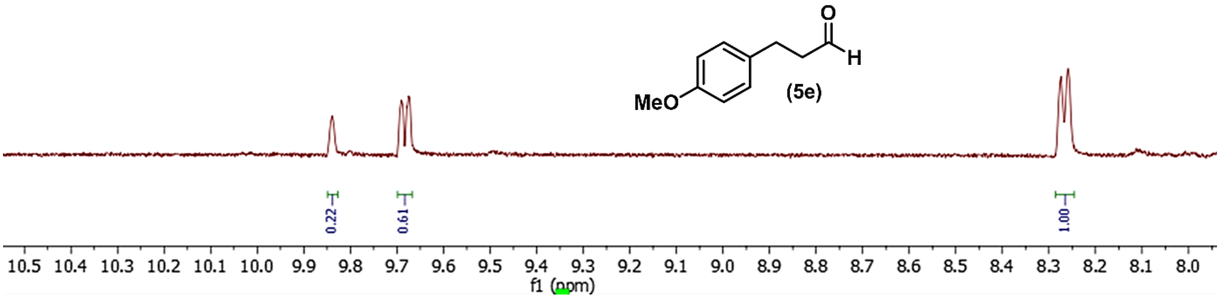

MeO
O
H
(5e)
0.22
0.61
1.00
10.5 10.4 10.3 10.2 10.1 10.0 9.9 9.8 9.7 9.6 9.5 9.4 9.3 9.2 9.1 9.0 8.9 8.8 8.7 8.6 8.5 8.4 8.3 8.2 8.1 8.0
f1 (ppm)

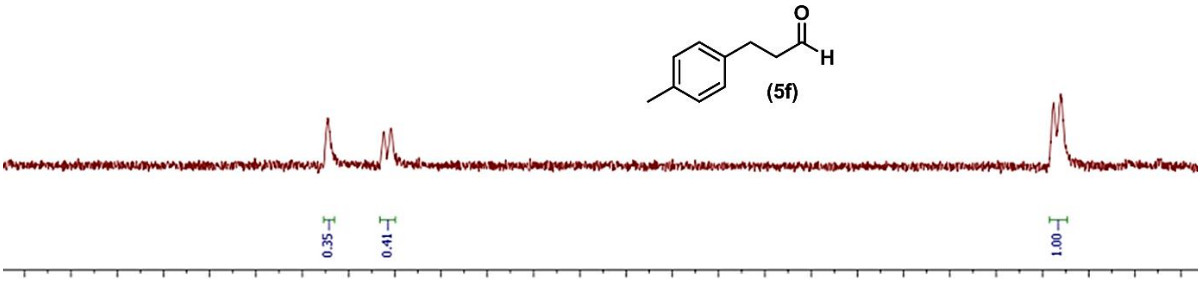

O
H
(5f)
0.35
0.41
1.00

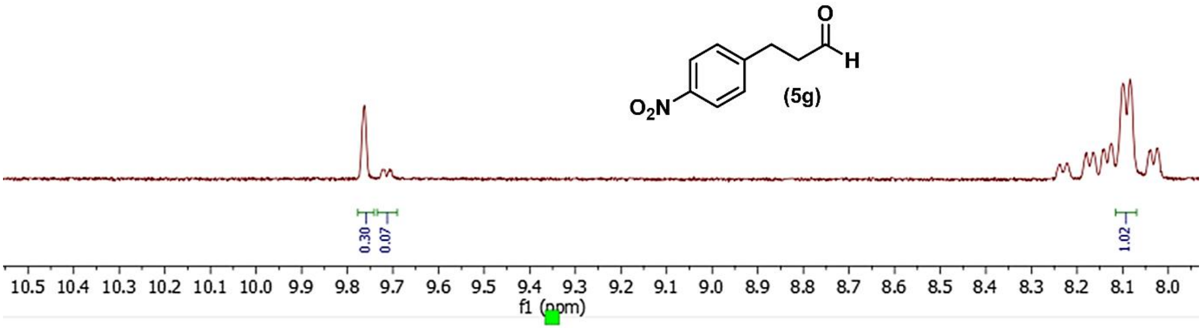

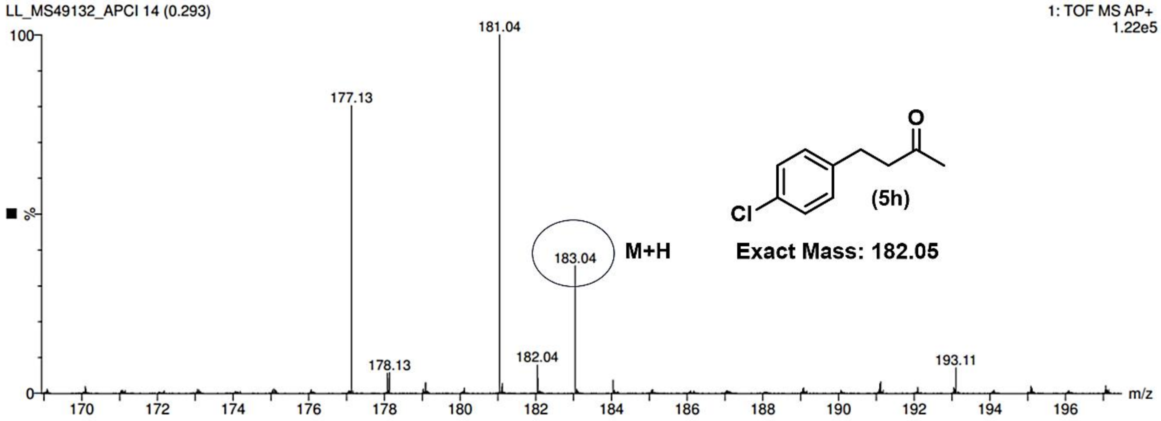

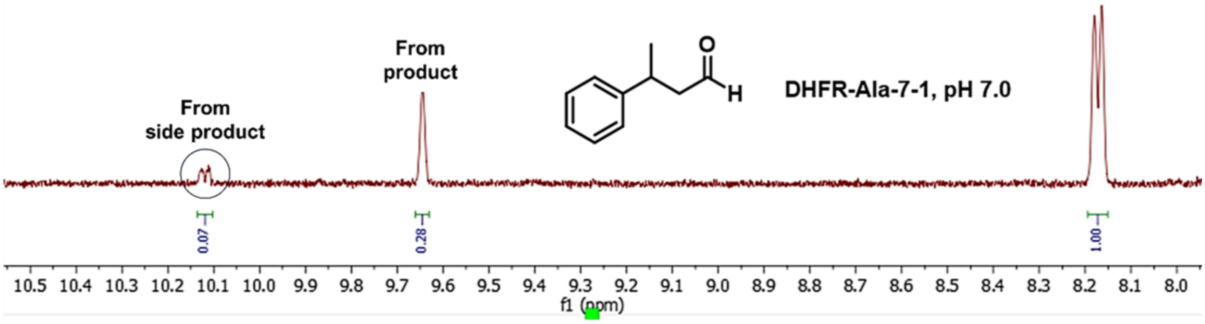

Possible reasons for side-product formation in 5i:

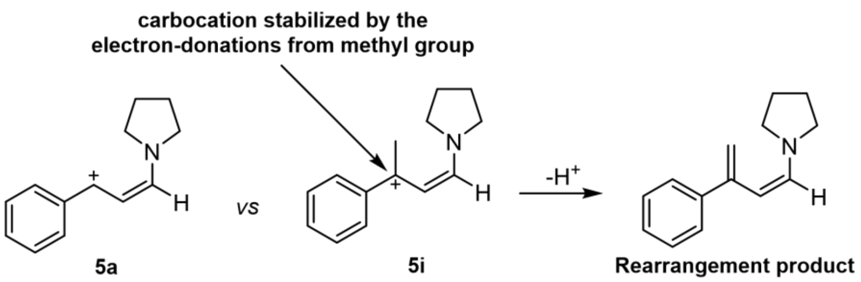

Figure S18. NMR and mass spectra of **5a-i**.

18. References

1. D. Sokolova, K. Tiefenbacher, *RSC Advances* **2021**, *11*, 24607-24612.

2. C. R. Polycarpo, S. Herring, A. Berube, J. L. Wood, D. Soll, A. Ambrogelly, *FEBS Lett.* **2006**, *580*, 6695-6700.

3. D. P. Nguyen, T. Elliott, M. Holt, T. W. Muir, J. W. Chin, *J Am Chem Soc* **2011**, *133*, 11418-11421.

4. M. Ismail, L. Schroeder, M. Frese, T. Kottke, F. Hollmann, C. E. Paul, N. Sewald, *ACS Catal.* **2019**, *9,* 1389-1395.

5. E. J. Loveridge, R. K. Allemann, *ChemBioChem* **2011**, *12*, 1258-1262.

6. K. Wang, A. Sachdeva, D. J. Cox, N. M. Wilf, K. Lang, S. Wallace, R. A. Mehl, J. W. Chin. *Nat. Chem.* **2014**, *6*, 393-403.

7. T. L. Williams, D. J. Iskandar, A. R. Nodling, Y. Tan, L. Y. P. Luk, Y. H. Tsai, *Amino Acids* **2021**, *53*, 89-96.

8. L. Y. P. Luk, J. J. Ruiz-Pernía, W. M. Dawson, M. Roca, E. J. Loveridge, D. R. Glowacki, J. N. Harvey, A. J. Mulholland, I. Tuñón, V. Moliner, R. K. Allemann, *Proc. Natl. Acad. Sci. USA* **2013**, *110*, 16344-16349.

9. R. S. Swanwick, G. Maglia, L.H . Tey, R. K. Allemann, *Biochem. J.* **2006**, *394*, 259-265.

10. S. Cattaneo, S. J. Freakley, D. J. Morgan, M. Sankar, N. Dimitratosa, G.J . Hutchings, *Catal. Sci. Technol.* **2018**, *8*, 1677-1685.

11. D. Sandner, U. Krings, R. G. Berger, *Z. Naturforsch C. J. Biosci*. **2018**, *73*, 67-75.

12. A. R. Nödling, K. Świderek, R. Castillo, J. W. Hall, A. Angelastro, L. C. Morrill, Y. Jin, Y. -H. Tsai, V. Moliner, L. Y. P. Luk, *Angew. Chem. Int. Ed.* **2018**, *57*, 12478–12482.

13. N. Santi, L. C. Morrill, K. Swiderek, V. Moliner, L. Y. P. Luk, *Chem. Commun.* **2021**, *57*, 1919-1922.

14. T. M. Bräuer, Q. Zhang, K. Tiefenbacher, *J. Am. Chem. Soc.* **2017**, *139*, 17500-17507.

15. M. BT, Z. Qi, T. Konrad, *Angew. Chem. Int. Ed.* **2016**, *55*, 7698-7701.

16. S. G. Ouellet, J. B. Tuttle, D. W. MacMillan, *J. Am. Chem. Soc.* **2005**, *127*, 32-33.

17. M. R. Sawaya, J. Kraut, *Biochemistry* **1997**, *36*, 586-603.

18. Y. Duan, C. Wu, S. Chowdhury, M. C. Lee, G. Xiong, W. Zhang, R. Yang, P. Cieplak, R. Luo, T. Lee, J. Caldwell, J. Wang, P. Kollman, *J. Comput. Chem.* **2003**, *24*, 1999-2012.

19. N. Holmberg, U. Ryde, L. Bülow, *Protein Engineering Design & Selection* **1999**, *12*, 851-856.

20. J. Wang, R. M. Wolf, J. W. Caldwell, P. A. Kollman, D. A. Case, *J. Comput. Chem.* **2004**, *25*, 1157-1174.

21. A. Jakalian, D. B. Jack, C. I. Bayly, *J. Comput. Chem.* **2002**, *23*, 1623-1641.

22. J. Wang, W. Wang, P.A. Kollman, D. A. Case, *J. Chem. Inf. Model.* **2006**, *25*, 247-260.

23. C. R. Sondergaard, M. H. Olsson, M. Rostkowski, J. H. Jensen, *J. Chem. Theory Comput.* **2011**, *7*, 2284-2295.

24. M. H. M. Olsson, C. R. Sondergaard, M. Rostkowski, J. H. Jensen, *J. Chem. Theory Comput.* **2011**, *7*, 525-537.

25. J. Mongan, D. A. Case, J. A. McCammon, *J. Comput. Chem.* **2004**, *25*, 2038-2048.

26. J. C. Phillips, R. Braun, W. Wang, J. Gumbart, E. Tajkhorshid, E. Villa, C. Chipot, R. D. Skeel, L. Kalé, K. Schulten, *J. Comput. Chem.* **2005**, *26*, 1781-1802.

27. J. Huang, A. D. MacKerell Jr, *J. Comput. Chem.* **2013**, *34*, 2135-2145.

28. C.E.A. Schafmeister, W.S. Ross, V. Romanovski. "LEAP, University of California, San Francisco, (1995).

29. W. L. Jorgensen, J. Chandrasekhar, J. D. Madura, R. W. Impey, M. L. Klein, *J. Chem. Phys.* **1983**, *79*, 926-935.

30. Y. Duan, C. Wu, S. Chowdhury, M. C. Lee, G. Xiong, W. Zhang, R. Yang, P. Cieplak, R. Luo, T. Lee, J. Caldwell, J. Wang, P. Kollman, *J. Comput. Chem.* **2003**, *24*, 1999-2012.

31. G. S. Grest, K. Kremer, *Physical Review A* **1986**, *33*, 3628-3631.

32. G. J. Martyna, D. J. Tobias, M. L. Klein, *J. Chem. Phys.* **1994**, *101*, 4177-4189.

33. D. R. Roe, T. E. Cheatham III, *J. Chem. Theory Comput.* **2013**, *9*, 3084-3095.

34. D. A. Case*, et al. AMBER, University of California, San Francisco*, (2021).

35. M. J. S. Dewar, E. G. Zoebisch, E. F. Healy, J. J. P. Stewart, *J. Am. Chem. Soc.* **1985**, *107*, 3902-3909.

36. J. Stewart, F. Seiler, QCPE Program No. 455, Quantum Chemistry Program Exchange. University of Indiana Bloomington, (1989).

37. M. J. Field, M. Albe, C. Bret, F. Proust-De Martin, A. Thomas, *J. Comput. Chem.* **2000**, *21*, 1088-1100.

38. A. Krzemińska, P. Paneth, V. Moliner, K. Świderek, *J. Phys. Chem. B* **2015**, *119*, 917-927.

39. B. Roux, *Comput. Phys. Commun.* **1995**, *91*, 275-282.

40. G. M. Torrie, J. P. Valleau, *J. Comput. Physics* **1977**, *23*, 187-199.

41. S. Kumar, J. M. Rosenberg, D. Bouzida, R. H. Swendsen, P. A. Kollman. *J. Comput. Chem.* **1992**, *13*, 1011-1021.

42. A. Krzemińska, V. Moliner, K. Świderek, *J. Am. Chem. Soc.* **2016**, *138*, 16283-16298.
